# A novel approach to data integrity auditing in PCS: Minimising any Trust on Third Parties (DIA-MTTP)

**DOI:** 10.1371/journal.pone.0244731

**Published:** 2021-01-07

**Authors:** Reem Almarwani, Ning Zhang, James Garside

**Affiliations:** 1 College of Computer Science and Engineering (CCSE), Taibah University, Medina, Saudi Arabia; 2 Information Management Research Group, The Department of Computer Science, The University of Manchester, Manchester, United Kingdom; University College of Engineering Tindivanam, INDIA

## Abstract

Data Integrity Auditing (DIA) is a security service for verifying the integrity of outsourced data in Public Cloud Storage (PCS) by users or by Third-Party Auditors (TPAs) on behalf of the users. This paper proposes a novel DIA framework, called DIA-MTTP. The major novelty of the framework lies in that, while providing the DIA service in a PCS environment, it supports the use of third parties, but does not require full trust in the third parties. In achieving this property, a number of ideas also have been embedded in the design. These ideas include the use of multiple third parties and a hierarchical approach to their communication structure making the service more suited to resource-constrained user devices, the provision of two integrity assurance levels to balance the trade-off between security protection levels and the costs incurred, the application of a data deduplication measure to both new data and existing data updates to minimise the number of tags (re-)generated. In supporting the dynamic data and deduplication measure, a distributed data structure, called Multiple Mapping Tables (M2T), is proposed. Security analysis indicates that our framework is secure with the use of untrusted third parties. Performance evaluation indicates that our framework imposes less computational, communication and storage overheads than related works.

## Introduction

One of the commonly used Cloud services is a Public Cloud Storage (PCS) service. PCS maintains and manages data for its customers. Users can access their data anywhere, at any time and with any device. Such services are typically provided over the Internet and charged on a pay-as-you-go basis. The services offer flexibility, and improved data availability and accessibility, so are increasingly popular among consumers. However, owing to the fact that providers are third-party service providers and the services are provided over open networks, the services are vulnerable to a large number of security threats and attacks [1]. The threats and attacks are not just from external entities but also authorised insiders. For example, a dishonest provider may delete data intentionally in violation of contracts to save storage space or may hide any accidental breach or loss of data to save their reputation. A disgruntled employee working for a provider may make unauthorised alterations to users’ data in an attempt to discredit his/her employer. A user may falsely accuse his/her provider of any data integrity or confidentiality breach in an attempt to obtain some financial gains unlawfully, etc. These security concerns are hindering the wide adoption of Cloud services in security sensitive areas, e.g. healthcare [2–4]. Data Integrity Auditing (DIA) is a security service used to ensure the integrity of outsourced data held in PCS.

At the centre of DIA are data integrity verification methods through the use of some proofs (also called tags or authenticators). There are broadly two classes of data integrity verification techniques, Proof of Retrievability (POR) [5] and Provable Data Possession (PDP) [6]. As we are interested in supporting both static as well as dynamic data, and the PDP based DIAs can support dynamic data integrity verifications more efficiently, hereafter we only focus on PDP-based DIAs and when we use DIA, we mean PDP-based DIAs.

### Motivation

Over the past few years, there are a good number of DIA related works published in literature [7–34]. These works can largely be classified into two main groups, the design of a tagging method for the generation and verification of integrity tags [7–21], and the design of a DIA system for ensuring the integrity of outsourced files via the use of a tagging method [14, 22–34]. The state-of-the-art tagging methods can be further classified into two categories, symmetric-key based [7–11, 35], and public-key based [12–18]. The symmetric-key based methods can only support private verifiability, making them unsuited to TPA-based DIA (Public DIA) or in an environment where third parties cannot be trusted unconditionally. The asymmetric-key based methods, such as RSA based [7, 13, 15] and short Boneh-Lynn-Shac-ham (BLS) based [16–20, 36], can support both public and private verifiability, but these methods are costly to the user end (Private DIA), particularly if users have a large number of files in the PCS.

Most of the state-of-the-art DIAs proposed so far either make use of a centralised system architecture [22–31] or assume that the providers and/or TPAs are unconditionally trustworthy [31–34]. The centralised system architecture is vulnerable to performance and reliability bottleneck. The assumption of providers/TPAs being unconditionally trustworthy may not be valid in many cases, as indicated by a recent study that 34% of threats in 2018 are from authorised insiders and it is increasing every year. A few that make use of a distributed system architecture [13, 33, 34] rely on the use of data redundancy to enhance reliability. This approach imposes a high level of computational and communication costs on users. In addition, the existing solutions have not considered how to support dynamic data with confidentiality preservation and tag collision resistance.

Our work reported in this paper is set to address these limitations and weaknesses, for which we have formulated the following research questions: (1) how to minimise trust on the third party service providers, (2) how to provide a DIA service securely and reliably with minimal costs, particularly for the user end, and (3) how to balance the trade-off between costs and security protection levels.

DIA in PCS with Minimising any Trust on Third Parties (DIA-MTTP) framework is designed to address the limitations discussed above. The design has made use of the following ideas: (i) a Multi-PCS-Multi-TPA system model; (ii) two levels of data integrity protections, LoA1 (Level of Assurance 1) supported by using public verifiability and LoA2 supported by using both public and private verifiability; (iii) two levels of data deduplication and at both the data block level during data uploading at both the user end and the service provider end; and (iv) an integrated data updating and data deduplication using a novel data structure. The framework has been analysed and evaluated in terms of security and performance, and the results of the analysis and evaluation have been compared with related work, demonstrating that the framework is more secure and more efficient than the related work.

### Contribution

Our contributions can be summarised as the following:

- Specify a set of requirements for the design of an effective, secure and efficient DIA.
- A critical analysis of the existing DIA against the requirements to identify their strengths and weaknesses.
- Design a novel DIA framework, DIA-MTTP, can satisfy all the requirements.
- Prove that DIA-MTTP can satisfy the security requirements through theoretical analysis.
- Justify the performance of DIA-MTTP through theoretical and experimental analysis and comparisons with the state-of-the-art.

### Organization

The rest of the paper is structured as follows. Requirement Specification Section presents a set of requirements for the design of a DIA framework. Based on the requirements, Related Work Critical Analysis Section critically analyses related DIA solutions published in literature, identifying areas for improvements. Key Features and Ideas Section presents our vision in designing a novel DIA framework, the DIA-MTTP. Our design preliminaries and building blocks are given in Design Preliminaries Section and Building Blocks Section, respectively. DIA-MTTP Functional Blocks Section highlights the architecture and functional blocks of DIA-MTTP, namely, Data Deduplication and Data Uploading (D3U), LoA1 Data Verification (LoA1DV), LoA2 Data Verification (LoA2DV) and Data Updating (DU). These functional blocks are discussed, respectively, in Data Deduplication and Data Uploading (D3U) Section, LoA1 Data Verification (LoA1DV) Section, LoA2 Data Verification (LoA2DV) Section and Data Updating (DU) Section, respectively. The security analysis of the DIA-MTTP framework is given in Correctness and Security of DIA-MTTP Verification Protocols Section and the performance evaluation in Performance Evaluation of the DIA-MTTP Section. Conclusion Section concludes the paper.

## Requirement specification

Based on the threat analysis in [37] and usecase study, we have specified a set of requirements for the design of an effective, secure, reliable and efficient DIA. The requirements can be classified into four groups, functional, security, reliability and performance requirements. Their details have been given in S1 File.

(F) Functional Requirements: (F1) Support Data/Tags Deduplication and (F2) Support Dynamic Data/Tags.
(S) Security Requirements: (S1) Resistance of providers cheating, (S2) Resistance of TPAs cheating and (S3) Resistance of users cheating.
(R) Reliability Requirements: (R1) Data Recovery and (R2) Elasticity.
(P) Performance Requirements: (P1) Minimizing Data Uploading Computational Cost, (P2) Minimizing Data Verification Computational Cost, (P3) Minimizing Data Updating Computational Cost, (P4) Minimizing Data Uploading Communication Cost, (P5) Minimizing Data Updating Communication Cost, (P6) Minimizing Data Verification Communication Cost and (P7) Minimizing Storage Overhead Cost.

## Related work critical analysis

There are a good number of DIA works published in literature, where the first DIA proposed by Ateniese et al. [7]. The DIA uses hashing based tagging method. The user before applying the tag generation algorithm, she/he applies error-correcting codes (ECCs) on the data file for data recovery property [38]. Another DIA was proposed by Chen et al. [8]. The Chen DIA is similar to the Ateniese_1 DIA except in term of using Algebraic signature based tagging method for more efficient DIA. It allows to perform a batch verification.

Above the two DIAs can be vulnerable to provider cheating (i.e. replay attacks). Thus, Sookhak et al. [9] proposed their DIA and use some random numbers, called nonces, in each data verification to resist the attack. The user chooses a different nonce for each selected data block. The provider should use these nonces in generating the proofs. Therefore, the user can be assured of receiving fresh proof each time, without the need to choose a large number of the data blocks.

In the case of the dynamic data, the provider may cheat the verifier using old data and their associated tags that are no longer valid more in the proof generation. Therefore, the existing DIAs use one of data structures to track and authenticate the data update and making the DIA more resistant to replace attacks. The Sookhak DIA used Multiple Index Hash Table (MIHT) [9]. The values of the rows are used in generating tags of the file for preventing the forging attack. It saves computational cost at the user end when using multiple tables (MIHT) instead of one table (IHT). In case of insertion or deletion operations and use one table for all data blocks, it can lead to recompute a high number of tags (i.e. in the worst case, all tags of the file may be recomputed). This is because of the serial numbers of data blocks under the updated rows should be alighted, and the tags that are associated should be recomputed, as a result.

Zhang et al. [10] proposed DIA and used Balanced Update Tree (BUT). In BUT, each node associated with one update request. The size of the BUT is independent of the total data blocks number for the file and grows linearly with the number of dynamic operations, unlike IHT. Therefore, it is more efficient in the case of the file that has a high number of the data blocks and rarely updated. The user and the provider should update their trees after each operation.

Three of DIAs proposed by Luo et al. [35], Xu et al. [11] and Krishra et al. [39]. The Luo DIA is different from the Xu DIA and the Krishra DIA by using the algebraic signature based tagging method and the IHT, while the Xu DIA uses homomorphic MAC (HomMAC) based tagging method and the Krishra DIA uses symmetric encryption algorithm.

The user and TPA, in above DIAs, should be entirely honest (i.e. they are private DIAs). Therefore, to make a DIA to be more secure (e.g. against non-repudiation attacks), Therefore, a number of DIAs have been proposed, where they used asymmetric key based tagging methods to support the public verifiability.

Ateniese et al. [12] proposed another DIA and used an RSA based tagging method. For data confidentiality preservation and data recovery, the user, before generating tags for a file, first encrypts the file, which is then encoded using an ECC method. Erway et al. proposed another DIA. [14]. It is similar to the Ateniese_2 DIA except in terms of using a Rank based Authenticated Skip List (RASL) data structure. As both DIAs use the RSA based tagging methods, thus, they can lead to higher computational and communication costs at the user end and storage cost at the provider end. Therefore, Hanser et al. proposed their DIA and used an ECDSA based tagging method alternatively [15].

For more efficiency, Li et al. [16], Liu et al. [17], Wang et al. [18–20], Tian et al. [40], Yang et al. [21], and Li et al. [41] proposed their DIAs using BLS based tagging methods to further reduce uploading communication and storage overheads. The difference between these DIAs is in a way that is used for preventing the provider cheating (replace attacks), i.e. tag collision prevention in the tagging methods. The Liu_1 DIA and the Yang_1 DIA use a hash of the data block in the tag generation, but in the Li_1 DIA and the Wang DIA use a block index. Furthermore, as using encryption at file-level may make data updating operations are inefficient where the user should retrieve the whole file for each update, therefore, the Liu_1 DIA, the Wang DIA and the Tian DIA use a random masking method. The random masking method is used to disguise the content of the data blocks when they are being released from the PCS upon receipt of an integrity verification request. Consequently, the TPA can verify the correctness of the proofs and cannot derive the user’s data plainly and violate their confidentiality. Additionally, the Li_1 DIA and Yang_1 DIA used IHT, the Liu_1 DIA and the Wang DIA used Merkle Hash Tree (MHT), and the Tian DIA used Dynamic Hash Table (DHT), while the Li_2 DIA uses Large Breaching Tree (LBT).

Using ECCs can work only on a specified number of losses, and, if it goes beyond this number, the original data cannot be recovered. Therefore, Liu et al. [22], Abo-alian et al. [23, 24], and Curtmola et al. [25] use another approach, i.e. The user uploads multiple replicas for his file to the PCS, and the TPA can check the integrity of all these replicas (data replication). Since a dishonest provider can delete one of these replicas without knowing the user, these replicas should, therefore, be distinguished from one another, and different associated tags are computed, as a result. In the Liu_2 DIA, a different random number used in each replica and then tags for each replica are generated. The Abo-alian_1 DIA uses an encryption algorithm alternative to the random numbers. Also, the data confidentiality can be preserved. For supporting dynamic data, the Abo-alian_2 DIA uses RASL.

In the above DIAs, the computational and communication costs of data uploading increase linearly with the number of data replicas at the user end. The more data replicas, more computational and communication costs. Therefore, the Curtmola DIA has been purposed to allows the user to generate one version of tags that can authenticate all data replicas by extracting a random number from a replica before it can verify its integrity. However, in the case of dishonest TPA, it may collude with the provider and share these random numbers.

Data deduplication can bring benefits, which it can release a user from sending a whole data file, so saving bandwidth at the user and minimising the storage overheads at the PCS. Therefore, Yuan et al. [26], Li et al. [27], Ma et al. [28] and He et al. [29] proposed their DIAs and integrated them with data deduplication. The user should provide evidence by sending the hash value of the file or random data blocks from the file. Once the user succeeds, then he can generate his tags and upload them to the PCS without the data file.

As the file confidentiality can be vulnerable to compromise when applying the data deduplication among multiple users in the above DIA; thus, the encryption should be used. Unfortunately, using conventional encryption with different signing keys results in different ciphertexts being generated and will outright incapacitate data deduplication as a result. Therefore, the Li_3 DIA, the Ma DIA and the He DIA applied other types of encryption algorithms to preserve data confidentiality. The Li_3 DIA and the Ma DIA use a convergent encryption [42]. The convergent encryption maybe not secure, whereby attackers may access the hash value by guessing, i.e. it is vulnerable to brute-force attacks. Therefore, a proxy encryption [43] is used in the He DIA, where each user can use his/her key. Then, the proxy can re-encrypt the data to allow deduplication to be applied, where this new entity should be trustworthy for a good security level. However, the Yune DIA, Li_2 DIA and He DIA incur a cost in term of computational. When the duplication is detected, the user still should generate his tags. Therefore, Liu et al. [22] proposed for their DIA to use re-signature proxy BLS based. The user, who is the first user who uploads a file, only processes the file, generates their associated tags and uploads them to the PCS. Thus, when another user has passed the ownership verification, then he can share his key for the tag generation with the provider for generating the user’s tags.

Using a single TPA in DIA can make the DIA vulnerable to a bottleneck problem and cause a delay in response when a high number of verification requests is received. Thus, using multiple TPAs could be a solution to avoid this issue. Abbdal et al. [30] proposed a DIA using an ECDSA based tagging method and two TPAs types, i.e. main TPA and multiple secondary TPAs. In the Abbdal DIA, when the main TPA is busy, the verification request is forwarded to the secondary TPA. Saxena et al. [31] releases the user from tag generation and multiple TPAs. In each data integrity verification, multiple TPAs collaborate in the data verification. Jin et al. [32] proposed another DIA using multiple TPAs, one TPA is for performing the data verification and another TPA is for dynamic data, where it stores a data structure. The Jin DIA used an index switcher for supporting a dynamic data.

Using multiple PCSes is in order to enhance the data availability, therefore, Yang et al. [21], Ni et al. [13], Zhu et al. [33], and Liu et al. [34] proposed DIAs for the decentralised PCS. The Yang_2 DIA can incur communication costs at the user and TPA. The user uploads a file replica to each PCS, i.e. the more data replicas, the higher the communication cost incurred. Furthermore, it incurs high computational and communication costs at TPA, where it should communicate with each provider for verification. Consequently, the Ni DIA was proposed using a hierarchical approach. In the hierarchical approach, one of the providers is used as a leader between other providers; thus, the user only communicates with the leader to upload his data and their associated tags. The Zhu DIA proposed and used the same approach to avoid the communication cost at TPA, too. The TPA only communicates with the leader when checking the integrity of a file at multiple PCSes. In the Zhu DIA, the provider may defraud the TPA and colludes with other providers. To detect such an attack by the TPA, different replicas and their associated tags at each PCS can be used as a solution, but this can incur high computational overhead costs at the user end. Therefore, Liu_4 DIA uses another approach, i.e. extra data blocks and their associated tags are used rather than using multiple replicas. Each PCS has different extra data blocks and their associated tags, and they should be included in its proof. The providers do not know which the data blocks are being uploaded on its PCS, and they are used for identification.

## Key features and ideas

The DIA-MTTP has four novel features. This section gives theses features along with ideas used to achieve feature.

### The Multi-PCS_Multi-TPAs system model

The DIA-MTTP implements the idea of using entity redundancy to make the service more reliable and secure. It uses multiple PCSes that are each managed by an independent provider for data storage. There are multiple TPAs for data integrity verification on behalf of the users. Multiple PCSes can help to address data recovery and data availability. A data replication among multiple PCSes can overcome the limitations of the other techniques that are used in the existing works such as either encoding methods (i.e. ECCs) or data replication in a single PCS. The data can be recovered using one of the copies on other PCSes. This may resist complete data loss or modification and an outage service. In the event of a service outage or security attack in a single PCS such as a distributed denial-of-service (DDoS) attack, the users are not able to access the service. However, data replication among multiple PCSes can help the users to access their data by using another PCS.

For the same reasons as described above, we have also used multiple TPAs (i.e. the multi-TPA approach) in the DIA-MTTP design. Using multiple TPAs can help to reduce the risk of creating a performance bottleneck which can occur in the case of using one TPA to verify the data in a distributed provider system. The approach of using multiple TPAs and pairing each TPA with a separate PCS distributes data verification tasks as it allows each TPA to only communicate with one PCS during a data verification process. This can reduce data verification delays, speeding up the response times to users. In addition, this approach introduces TPA service redundancies for public verification, as each such verification involves the participation of multiple TPAs and their verification results are collated to produce the final result delivered to the user, thus protecting against collusions among the PCS providers, collusions among TPAs/providers and frame attacks by TPAs.

While the Multi-PCS_Multi-TPAs approach can provide benefits related to service reliability and security, it may increase the communication cost. To reduce this cost as much as possible, we have adopted a hierarchical approach to entity connections. We have classified multiple PCSes into one leader PCS and multiple non leader PCSes. We apply the same approach to multiple TPAs. In other words, there is a leader provider and a leader TPA. These leaders act as a gateway to their respective non leader entities. These leader entities operate during the data uploading and integrity checking options. The leader entities mainly play the role of managing and coordinating the users to ensure that they only communicate with leader entities, i.e. the leader provider and leader TPA. There is no direct communication between the users and the non leader providers in the TPAs.

### Two-level data integrity protection

To better balance the trade-off between security protection levels and the costs incurred by providing the protections, thus optimising performance, DIA-MTTP implements two Levels of data integrity Assurance (LoA), namely LoA1 (level 1) and LoA2 (level 2). LoA1 supports the use of public verification and it is intended for users with non-critical/low-sensitive data or users who have more trust in their service providers. LoA2 supports the use of both public and private verification (dual verification) and it is intended for users with critical/highly-sensitive data or users who have less trust in their service providers. By supporting dual verification, the users can also verify the integrity of their data directly, thus detecting any misbehaviours through either the providers or the TPAs. This private verification can be performed at any time and at any frequency.

### Two-level data deduplication

Data deduplication refers to the process of eliminating any redundant data. Eliminating redundant data can help to reduce computational, storage and communication costs. This is because tag generations and data encryption are only applied to non-duplicated data. As a result, the number of tags that should be generated and the number of data blocks that should be encrypted can be reduced, thus lowering the computational cost imposed on users and the storage cost imposed on the PCS. In addition, the amount of data poured onto the channel when the data are being uploaded can also be reduced, thus reducing the bandwidth and/or communication cost.

Data deduplication can be applied at both the block-level and the file-level [44]. Block-level data deduplication refers to the identification and elimination of redundant data blocks in one or more files. File-level data deduplication refers to the identification and elimination of completely redundant file(s). File-level data deduplication can only be applied to files that are 100% identical. Both deduplication operations are applied to files owned by the same user. Due to brute-force attacks and confidentiality concerns, the deduplication operations are not applied to files owned by different users [44, 45].

Data deduplication operations may be performed by a user, i.e. user-end data deduplication, and/or by a provider, i.e. PCS-end data deduplication [44]. User-end data deduplication is applied to data blocks/files that are to be uploaded by a user, whereas PCS-end data deduplication is applied to all of the data blocks/files that are being managed by the PCS for the user. To maximise cost reduction, DIA-MTTP employs block-level data deduplication and both user-end and PCS-end. DIA-MTTP supports two data deduplication levels, namely Data DeDuplication Level 1 (D3L1) and Data DeDuplication Level 2 (D3L2). D3L1 is a form of user-end data deduplication, while D3L2 is a form of PCS-end data deduplication. With this two-level data deduplication support, users may not need to compute any tags for the data if the data to be uploaded is duplicated.

### An integrated data updating and data deduplication

Implementing the idea of integration of the data updating and data deduplication makes DIA more efficient. As the outsourced data cannot only be static data files, users need to update their data at any time. Therefore, they should keep the data that are non-duplicated after each update. Furthermore, it can lessen the computational costs of the user, which can release him/her from computing a tag for the updated data block in a case where it is duplicated. Using the one data structure approach, i.e. a single data structure for tracking update all data files that are owned by one user, can help to implement this idea more efficiently than using a data structure for each file, i.e. file dependent data structure. The first approach allows for the provision of mapping information between one or more data files. Furthermore, it allows for the implementation of batch update operations, i.e. updating one or more data blocks in one or more files efficiently. In the latter approach, for batch updating, it should update each data structure that is associated with a file separately. This can consume more communication and computational costs on part of both the user and the provider.

To lessen the computational and communication costs during the data update and verification, each entity in the DIA-MTTP system has its own respective data structure, i.e. User-DS, TPA-DS, PCS-DS (i.e. the distributed data structure). It allows for the tracking of the data update and the mapping of the information of the data deduplication by all system entities, unlike using a centralised data structure, i.e. where only the entity that has the data structure can track the data update and deduplication. With a distributed data structure, the user or the verifier can be released from verifying or re-signing a root of a tree in the data updating or verification process. On the other hand, the provider can be released from sending auxiliary information during the data update or verification. To lessen the computational and communication costs during the data update and verification, each entity in the DIA-MTTP system has its own respective data structure, i.e. User-DS, TPA-DS, PCS-DS (i.e. the distributed data structure). It allows for the tracking of the data update and the mapping of the information of the data deduplication by all system entities, unlike using a centralised data structure, i.e. where only the entity that has the data structure can track the data update and deduplication. With a distributed data structure, the user or the verifier can be released from verifying or re-signing a root of a tree in the data updating or verification process. On the other hand, the provider can be released from sending auxiliary information during the data update or verification.

In addition to providing the properties described above, DIA-MTTP also preserves data confidentiality and resists replay attacks. Data confidentiality preservation is achieved using a block level encryption method that has been implemented in the TOD method. The block level encryption method relieves a provider from applying another method, i.e. random masking, to prevent the TPAs from accessing the content of the data that are to be verified. As mentioned in the existing works, the masking operation needs to be carried out by the provider every time a file integrity verification request is received. This imposes an additional run-time overhead on the provider. In addition, this approach does not protect the data confidentiality present against the providers. Replay resistance is provided using nonces. A nonce is generated and used in the verification request initiated by a verifier. The responder, a provider, should include this nonce in the reply (as proof of data integrity preservation). In this way, the verifier can be assured that the proof received from the provider is fresh.

## Design preliminaries

This section gives the design preliminaries, i.e. system architecture, assumptions and notations, under which the DIA-MTTP framework is designed and described.

### System architecture

**Fig 1:**
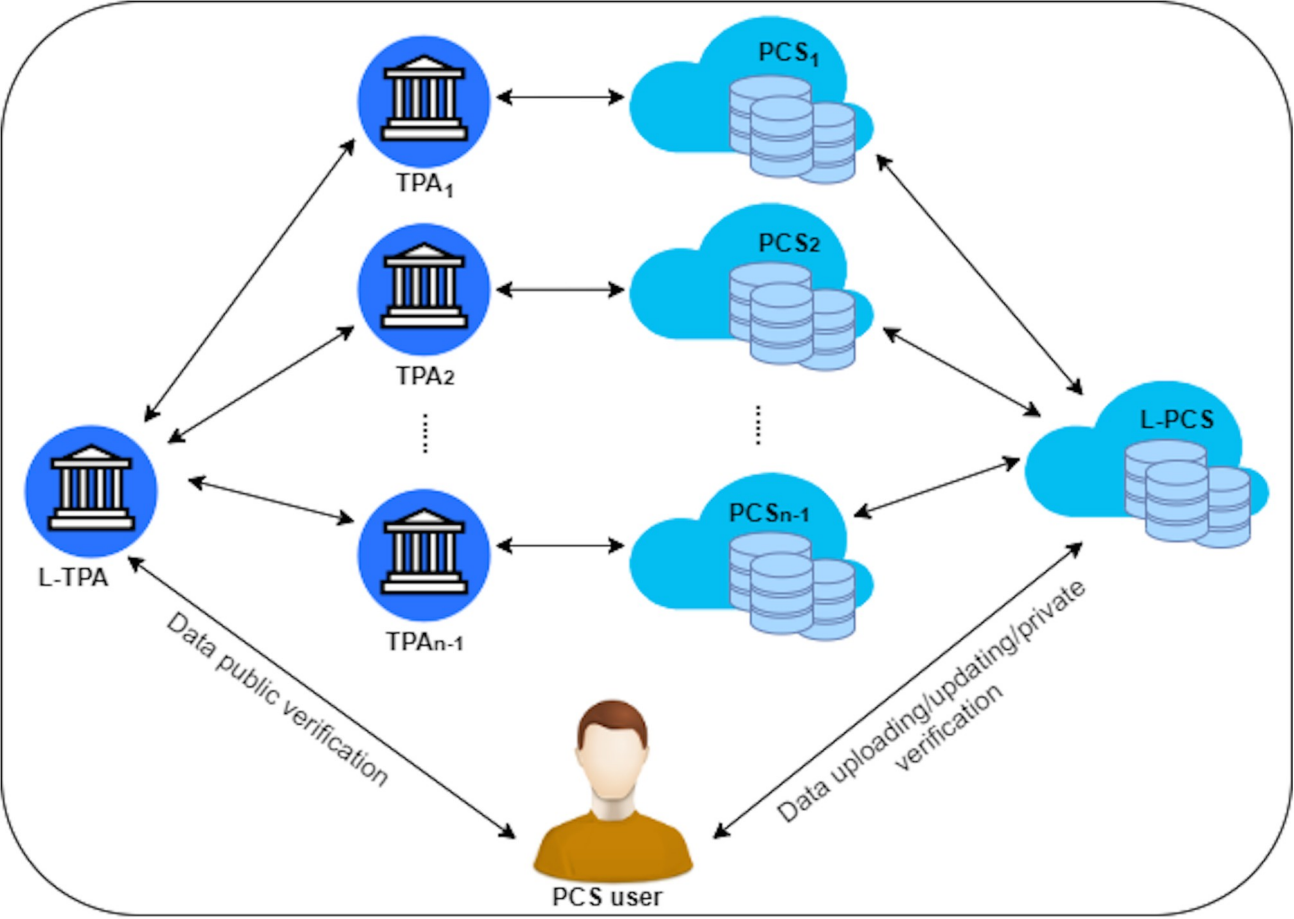
DIA-MTTP high-level architecture.

DIA-MTTP supports the use of *n* providers and *n* TPAs. Each PCS is associated with a separate TPA. The PCSes and TPAs are respectively layered at two levels, a leader level and a non-leader level. One of the providers is designated as a leader provider. The leader provider is responsible for managing the storage service provided by all the *n* providers and co-ordinating with PCS users and other (*n*-1) non leader providers during their data uploading/updating operations. Similarly, one of the TPAs is designated as a leader TPA (L-TPA). L-TPA is responsible for managing data integrity verifications collaboratively performed by the *n* TPAs for PCS users and co-ordinating with the PCS users and other (*n*-1) non leader TPAs during the verification operations. PCS users only need to communicate with the leader provider during their data uploading/updating operations and only need to communicate with the L-TPA during data verification operations. Fig 1 shows the top-level architecture of the DIA-MTTP framework.

### Assumption

To scope the work, the following assumptions are used for the design of the DIA-MTTP.

**(A1)** The focus of this work is on tackling insider threats in relation to data integrity. Some of the external attacks, such as impersonation, are outside the scope of this work. In other words, communication channels linking the DIA-MTTP entities are assumed to be authenticated using off-the-shelf technologies, e.g. Secure Socket Layer (SSL) [46].
**(A2)** The provider and the TPA may misbehave, committing fraud and forgeries in relation to data integrity, but they follow protocol specifications correctly.
**(A3)** The cryptographic algorithms and pseudo-random number generator used in the design are secure.
**(A4)** Cryptographic keys are securely generated, distributed and stored. All the public keys are certified and trusted by Certification Authorities (i.e., CAs).

### Notations

The notations used in the remaining part of this paper is summarised in Table 1.

**Table 1:** Notations used in the design of DIA-MTTP.

*DF*	Data file.
*DB*_*i*_	*i*^*th*^ data block in a data file.
{*DB*_*i*_}	Set of the data blocks.
*d*1	Number of data blocks of the file using D3L1.
*d*2	Number of data blocks of the file using D3L2.
*sk*	User’s LiSHE secret key.
*ppk*_*En*_	User’s Paillier public key.
*x*	User’s BLS private key.
*ppk*	User’s BLS public key.
*User*_*ID*_	ID of the owner of the file.
*RN*	Random number generated using a secure pseudorandom number generator.
*n*	Number of TPAs/PCSes in DIA-MTTP.
PCS provider_*i*_	Provider manages PCS_*i*_.
*TPA*_*i*_	*i*^*th*^ TPA in the non leader group.
DT	Number of rows that should be aligned in the IHT when updating a data block.
NP	Number of nodes in a path for a data block (DB) in the tree (i.e. MHT or LBT).

## Building blocks

The design of DIA-MTTP has made use of two main building blocks, a novel tagging method, the TOD method [47], and a data structure, Mapping Multiple Tables (M2T). TOD is used for tag generation and tag verification for outsourced data. M2T is used for handling dynamic data and data deduplication. The following first describes TOD and then M2T.

### TOD method

TOD has been described, analysed and evaluated in detail in [37]. As TOD is used as a building block in DIA-MTTP, we here provide a summary of the TOD method.

TOD is built on a number of cryptographic primitives, namely, the Li Symmetric homomorphic encryption (LiSHE) scheme [48], the Paillier Asymmetric homomorphic encryption scheme [49], algebraic signature [50] and BLS short signature [51]. It supports both public and private verifiability on the same platform with less overhead at the user end. This property is achieved by using four types of tags. The four types of tags are, respectively, an identifier tag (*IDTag*), a data tag (*DataTag*), a data block tag (*DBTag*), and a *DBTag* tag (*DBTagTag*). Table 2 shows how these tags can be generated. Furthermore, TOD uses block-level encryption to protect data confidentiality.

**Table 2:** Math equations for tag generation.

$\begin{array}{c} IDTag_{i}=AS(User_{ID}||RN_{i})\;(1) \end{array}$
$\begin{array}{c} DBTag_{i}=IDTag_{i}+DataTag_{i}\;(2) \end{array}$
$\begin{array}{c} DataTag_{i}=AS\left(En\_DB_{i}\right)\;(3) \end{array}$
$\begin{array}{c} DBTagTag_{i}={\left[H\left(En\_IDTag_{i}\right)\times \upsilon^{DBTagMapValue_{i})}\right]}^{x}\;(4) \end{array}$
$\begin{array}{c} AggEn\_DB=\sum_{i=0}^{C-1}En\_DB_{i}\;(5) \end{array}$
$\begin{array}{c} AggDBTag=\sum_{i=0}^{C-1}DBTag_{i}\;(6) \end{array}$
$\begin{array}{c} AggIDTag=\sum_{i=0}^{C-1}IDTag_{i}\;(7) \end{array}$
$\begin{array}{c} AggEn\_DBTag=AS(AggEn\_DB)\;(8) \end{array}$
$\begin{array}{c} AggDBTag=AggIDTag+AggEn\_DBTag\;(9) \end{array}$

### Multiple Mapping Tables (M2T)

The M2T is used for tracking data file updates and facilitating data deduplication. When data is uploaded by the user, the provider uses the M2T that is associated with a user to identify if there is any data that has been duplicated. If there is no duplication, then the data will be stored and the respective entries in the tables created. Otherwise, if any duplication is detected, then the respective entries will be updated and there will be no new data or tag insertions. The M2T is a tag-independent data structure. Unlike hashing-based tables (e.g. IHT), the M2T information (i.e. sequential values) is not to be used in tag generation. This means that updating a data block and its tag does not lead to updating unrelated tags.

**Fig 2:**
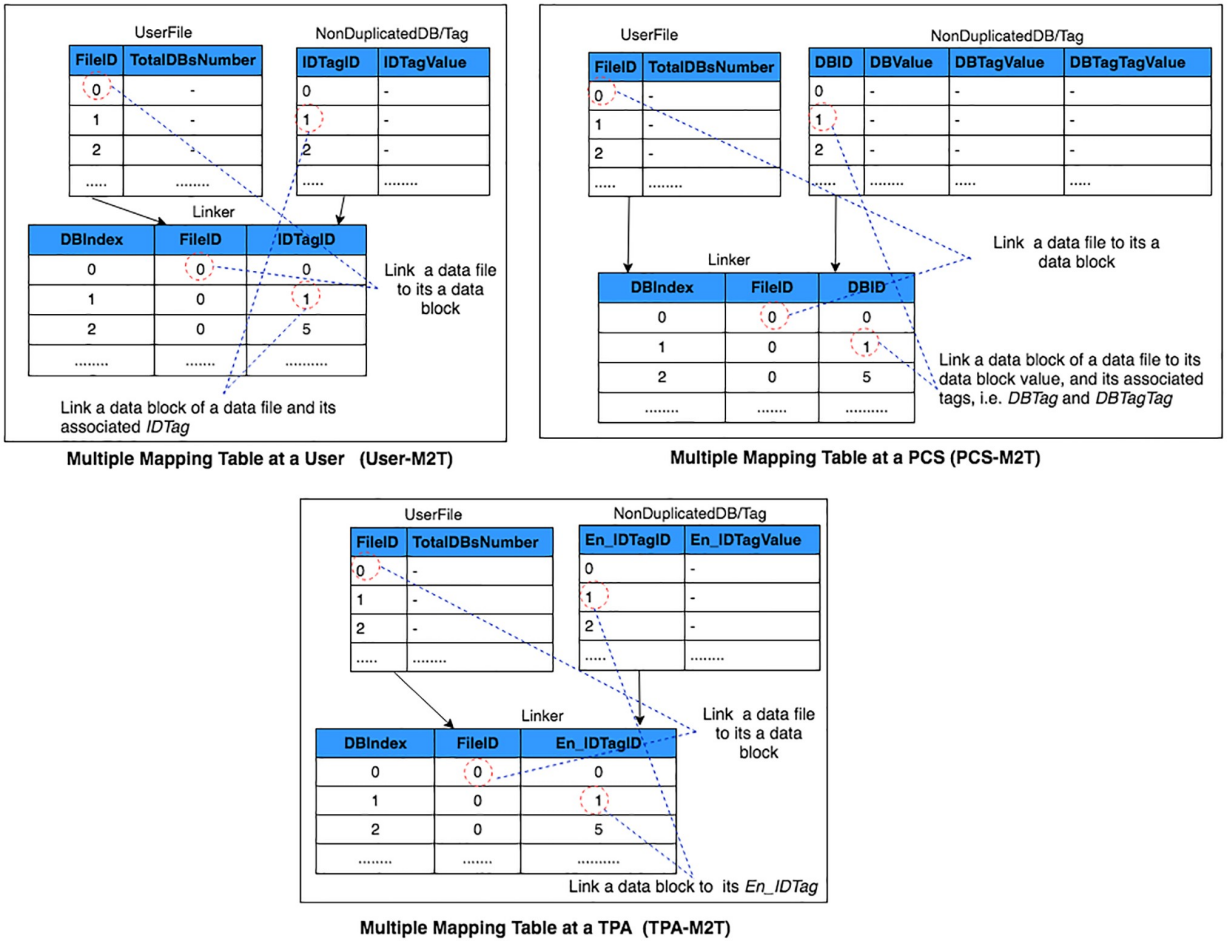
Distributed M2T data structure.

Furthermore, the M2T can support the distributed data structure. There are three types of M2Ts: User-M2T, PCS-M2T and TPA-M2T. User-M2T is used by the user to manage the updating of his/her data files in PCS and to provision their mapping information when data deduplication is performed. PCS-M2T is used by a provider to manage the updating of a user’s data files and to facilitate the L2 data deduplication, i.e. D3L2. TPA-M2T is used by a TPA to manage the updating of the user’s data files and the provision of their mapping information. The provider manages one M2T for each user. Similarity, the TPA manages one M2T for each user. There are three M2Ts associated with a user; one with the user, one with the provider and one with the TPA. Fig 2 shows the three M2T types that are associated with one user. As the DIA-MTTP supports the multiple PCSes, then there is a PCS-M2T associated with the user in each PCS.

To update the data, there are three operation types, i.e. a data block insertion, a data block modification and a data block deletion. Their details have been given in S2 File.

## DIA-MTTP functional blocks

**Fig 3:**
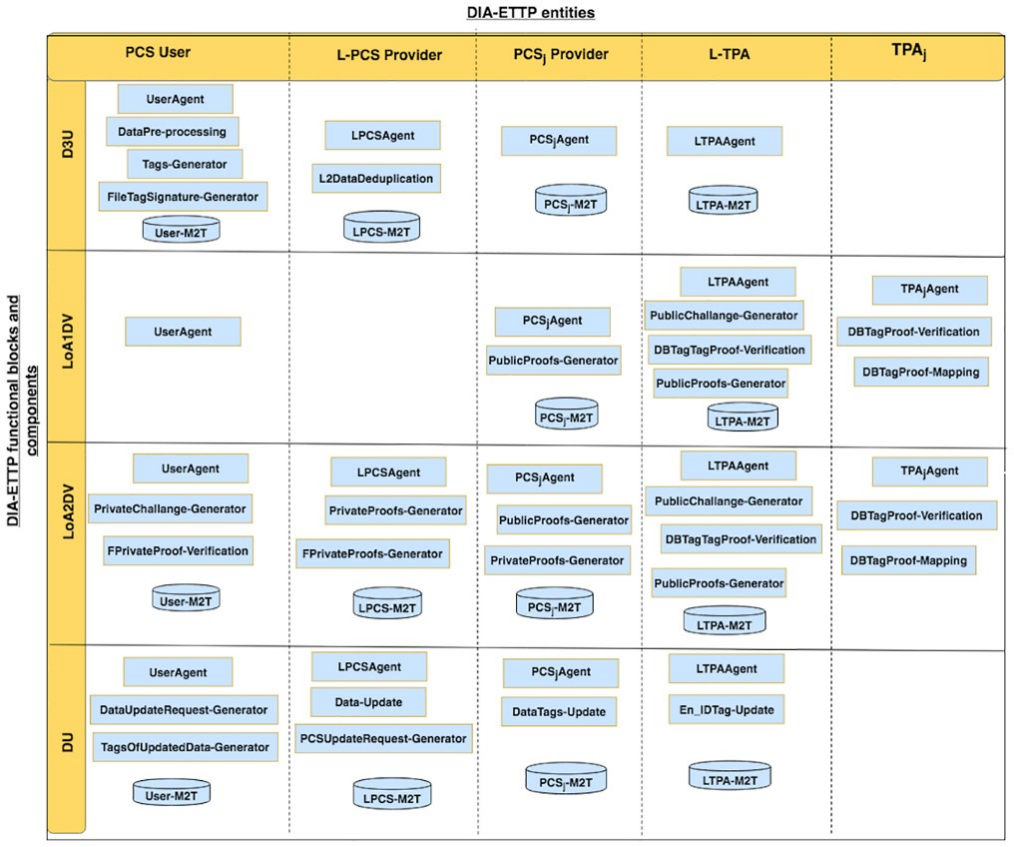
DIA-MTTP: Functional blocks, components and entities.

The DIA-MTTP consists of four major functional blocks, namely Data Deduplication and Data Uploading (D3U), LoA1 Data Verification (LoA1DV), LoA2 Data Verification (LoA2DV) and Data Updating (DU). The D3U is used for uploading data and the associated tags to multiple PCSes while applying two-levels of data deduplication. The LoA1DV is used for performing public verification (i.e. LoA1) concerning the data that have been outsourced to PCSes. The LoA2DV is used for performing both private and public verification (i.e. LoA2) related to the outsourced data files in PCSes, and the DU is used for updating the data and their associated tags in PCSes. Each block has a group of components which work collaboratively to achieve their duties. Each subgroup of components is managed by one of the entities. The major functional blocks, i.e. their entities and their associated components, have been outlined in Fig 3. The details of the DIA-MTTP functional blocks, i.e. D3U, LoA1DV, LoA2DV and DU, have been detailed in the following sections.

## Data deduplication and Data Uploading (D3U)

**Fig 4:**
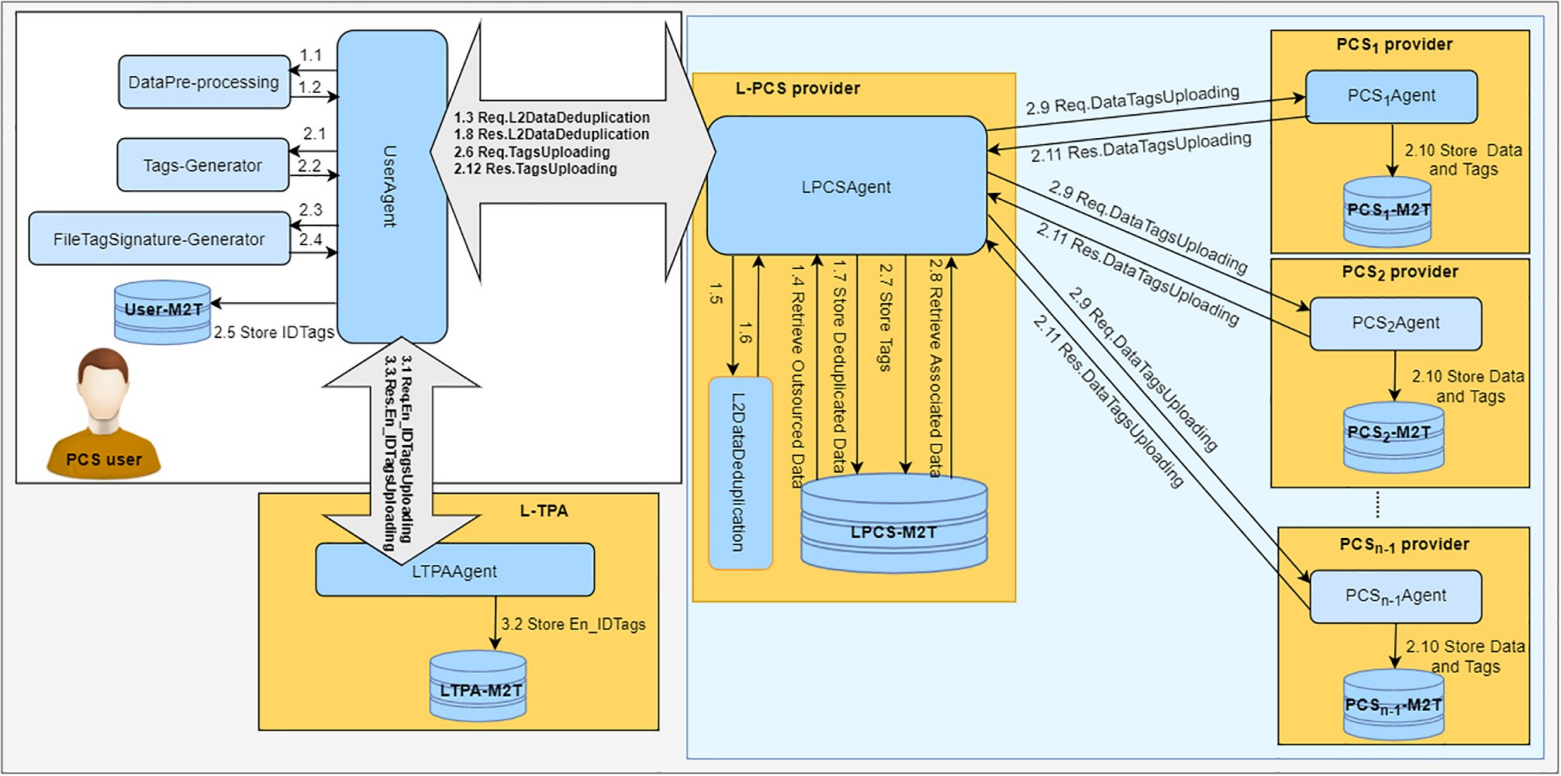
The D3U architecture.

The architecture of D3U is as shown in Fig 4. It a group of components and four types of entities, i.e. the user, the leader provider, the non leader provider and the L-TPA. Each entity manages its associated components in order to perform its duties optimally.

A detailed description of the four algorithms that are used for implementing the operations of the D3U components are given below.

**FileSetUp algorithm**: The FileSetUp algorithm is used for implementing the operations of the DataPre-processing functional component. The algorithm takes the data file (*DF*) and the user’s encryption key, *sk*, and outputs a set called the D3L1 Result (*D*3*L*1*R*). The user uses *sk* to encrypt all of the outsourced data files. The *D*3*L*1*R* consists of *K* items, i.e. *K* is a total blocks number of the data file. Each item in the *D*3*L*1*R* can be either *En*_*DB* value or an ID of the data block. The ID of the data block is one of the identifiers (indexes) of data blocks in the data file. It is associated with a data block that is identical to the given data block. In other words, if a data block in the *D*3*L*1*R*_*i*_ (*DB*_*i*_) is identical to another data block in the file (*DB*_*j*_), then the value of *D*3*L*1*R*_*i*_ is the ID of *DB*_*j*_ rather than the *DB*_*i*_ value. Thus, *D*3*L*1*R*_*i*_ contains non-duplicated data blocks or the associated links of the duplicated data blocks. The details of this algorithm are given in Algorithm 1 (see the DIA-MTTP algorithms in S3 File).

**L2DataDedup algorithm**: The L2DataDedup algorithm is used for implementing the operations of the L2DataDeduplication component. The duplicated data blocks are eliminated and only the non-duplicated data blocks are kept. The algorithm takes two sets, *D*3*L*1*R*, and the outsourced data blocks hash values set (*ODBH*) as the inputs and outputs three sets: the D3L2 Result (*D*3*L*2*R*), the non-duplicated data blocks set (*NDB*) and the non-duplicated data blocks hash values set (*NDBH*). The *D*3*L*2*R* is a set in which its items number is equal to the items number in *D*3*L*1*R*, i.e. *K*. Each item in *D*3*L*2*R* is associated with one data block. It can refer to either an empty value or ID of one of the non-duplicated data blocks (in NonDuplicatedDB/Tag table). When its value is empty, this means that its associated data block is a non-duplicated block. Otherwise, the data block is a duplicated block and its value is an ID of the data block that it is identical to the data block in the LPCS-M2T. The details of L2DataDedup algorithm are given in Algorithm 2.

**BlockTagGen algorithm**: The BlockTagGen algorithm is used for implementing the operations of the Tags-Generator component. It uses the TagGen algorithm of the TOD method for generating tags for the non-duplicated data blocks that are indicated in the *D*3*L*2*R*. The algorithm takes the data blocks, {*En*_*DB*_*i*_}, the ID of the user (*User*_*ID*_), a BLS private key, *x*, a random number, *υ*, and Paillier public key, *ppk*_*En*_, *D*3*L*1*R* and *D*3*L*2*R* and outputs five sets, i.e. {*IDTag*_*i*_}, {*En*_*IDTag*_*i*_}, {*DBTag*_*i*_}, {*DBTagTag*_*i*_} using Eq(1), Eq(2), Eq(3) and Eq(4) in Table 4, and *D*3*L*1*R*′. The user uses *x*, *υ*, and *ppk*_*En*_, to generate tags of all of the outsourced data files. *D*3*L*1*R*′ is another version of *D*3*L*1*R* but with the elimination of the data block values. The details of this algorithm are given in Algorithm 3.

**FileTagSigGen algorithm**: The FileTagSigGen algorithm is used to implement the operations of the FileTagSignature-Generator component. It is used for generating a signature for the file tag (*FileTag*) of the data file, i.e. *FileTagSig*. The algorithm takes the total number of the data blocks of the data file, *K*, the ID of the file, *FileID*, and a private key (*Pkey*) for signing, as the inputs. Its output is *FileTagSig* using Eq(10) in Table 2. It is used to authenticate the identifier of the data file and its total number of data blocks. The details of the FileTagSigGen algorithm have been given in Algorithm 4.

**Fig 5:**
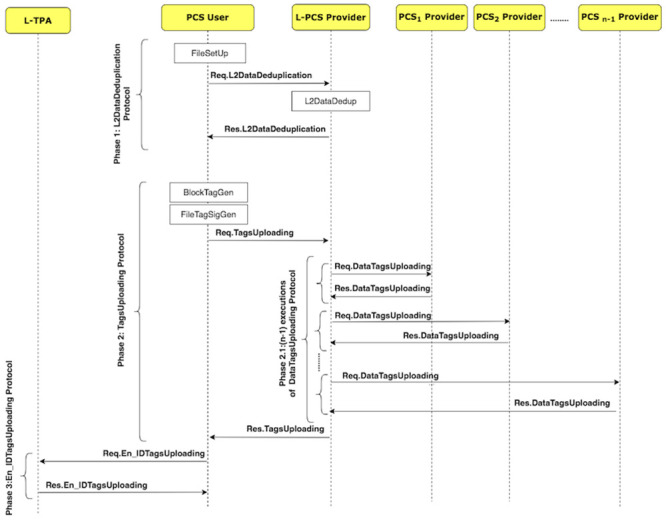
The D3U protocol suite.

**Fig 6:**
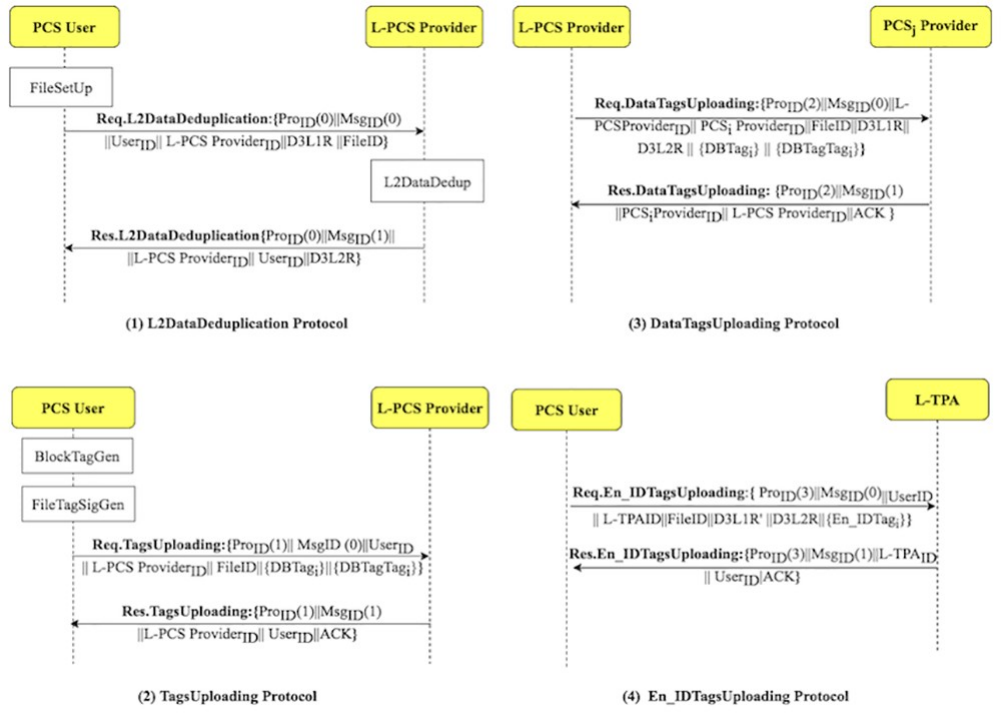
D3U protocol: Sub-protocols and their messages.

Using the D3U protocol, the user uploads the non-duplicated data with its associated tags to multiple PCSes. Fig 5 shows how the D3U protocol is executed by illustrating its sub-protocols. The protocol consists of four sub-protocols, namely, L2DataDeduplication, TagsUploading, DataTagsUploading and En_IDTagsUploading. The sub-protocols and their messages are shown in Fig 6.

## LoA1 Data Verification (LoA1DV)

**Fig 7:**
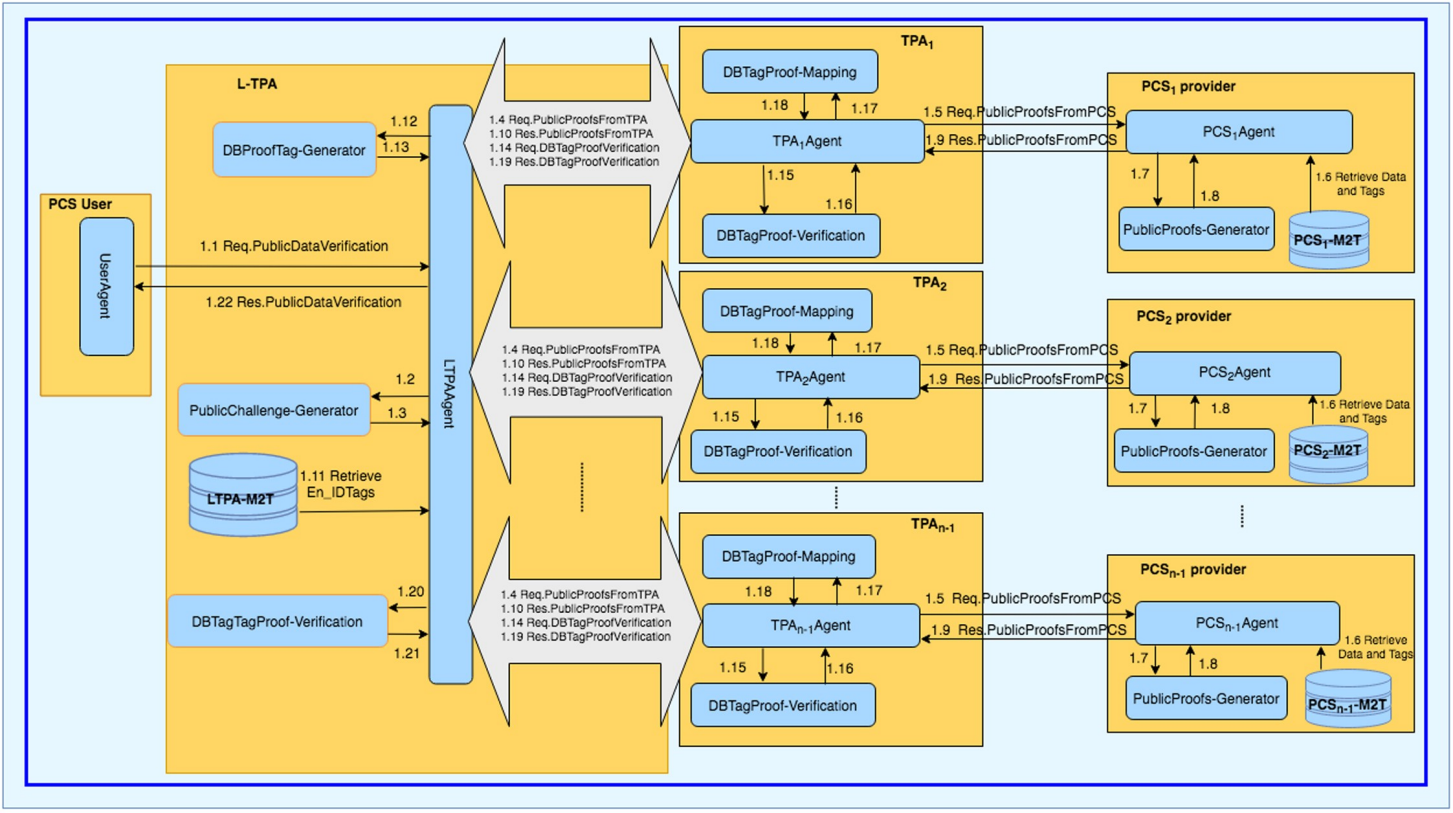
The LoA1DV architecture.

The LoA1DV architecture shown in Fig 7 includes a group of components and the following four types of entities, i.e. the user, the L-TPA, the non leader TPA and the non leader provider. The user, in LoA1DV, is only responsible for delegating the L-TPA to perform the public verification.

A detail the algorithms that are used to implement the components of the LoA1DV are given below.

**PubChalGen algorithm**: The PubChalGen algorithm is used for implementing the operations of the PublicChallange-Generator component. It is used for generating public verification challenges. Before generating the public verification challenges, the signature of *FileTag* should be verified to authenticate a file ID and its total number of data blocks. Thus the algorithm takes file tag signature, i.e. *FileTagSig*, and a public key used for decrypting *FileTagSig*, *Pbkey*, as the inputs, and it outputs public challenges, {*PubChall*_*j*_} (which each TPA has one PubChall), and a tag of an aggregated value of nonces (i.e. {*ProofNonce*_*i*_}), *AggProofNonceTag* using Eq(28) and AS (Table 3 presents all equations are used in LoA1DV). The *PubChall*_*j*_ consists of two sets, i.e., a set of indexes of *C* data blocks, {*I*_*i*_}, 0 ≤ i < *C*, and a set of the nonces ({*ProofNonce*_*i*_}), and the following three items: a nonce value (*PCSNonce*_*j*_), its tag (*PCSNonceTag*_*j*_) is computed using AS, and the ID of File (*FileID*). The {*I*_*i*_} values are used to indicate the positions of the chosen data blocks among the file blocks. For each *I*_*i*_, *ProofNonce*_*i*_ is chosen and for each PCS, *PCSNonce*_*j*_, is chosen. The two sets of nonces, {*ProofNonce*_*i*_} and {*PCSNonce*_*j*_}, are used to help to the replay attack prevention and the frame attack detection. The details of the PubChalGen algorithm have been given in Algorithm 5.

**Table 3:** Math equations for public verification.

$\begin{array}{c} FileTagSig=E_{Pkey}\left(FileTag||FileTagH\right)\text{,}\;\text{where}\;FileTagH\;\text{is}\;\text{hash}\;\text{value}\;\text{of}\;FileTag,\text{E}\;\text{is}\;\text{an}\;\text{encryption}\;\text{algorithm}\;(10) \end{array}$
$\begin{array}{c} PubDBProof_{j}=\sum_{i=0}^{C-1}\left(En\text{-}DB_{i}+ProofNonce_{i}\right)\;(11) \end{array}$
$\begin{array}{c} PubDBTagProof_{j}=\left\{PubDBTagProof_{ji}\right\},0\leq i<C,\text{,}\;\text{where}\;PubDBTagProof_{ji}=\;DBTag_{i}\;\text{+}\;PCSNonceTag_{j}\;(12) \end{array}$
$\begin{array}{c} AggDBTagProof=\sum_{i=0}^{C-1}\left(PubDBTagProof_{ji}\right)+AggProofNonceTag\;(13) \end{array}$
$\begin{array}{c} PubDBTagTagProof_{j}={\left(\prod_{i=0}^{C-1}DBTagTag_{i}^{ProofNonce_{i}}\right)}^{PCSNonce_{j}}\;(14) \end{array}$
$\begin{array}{c} DBProofTag^{\prime }=DBProofTag_{j}+\left(C\times PCSNonceTag_{j}\right)\;(15) \end{array}$
$\begin{array}{c} En\_DBProofTag=En(DBProofTag^{\prime })\;(16) \end{array}$
$\begin{array}{c} En\_AggDBTagProof^{\prime }=AggEn\_IDTag\times En\_DBProofTag\;(17) \end{array}$
$\begin{array}{c} En\_AggDBTagProof==En\_AggDBTagProof^{\prime }\;(18) \end{array}$
$\begin{array}{c} DBTagProofMapValue_{j}=\sum_{i=0}^{C-1}DBTagMapValue_{ji}^{ProofNonce_{i}}\;(19) \end{array}$
$\begin{array}{c} AggPCSNonce=\sum_{j=0}^{n-2}PCSNonce_{j}\;(20) \end{array}$
$\begin{array}{c} AggDBTagTagProof={\left(\prod_{j=0}^{n-2}PubDBTagTagProof_{j}\right)}^{1/AggPCSNonce}\;(21) \end{array}$
$\begin{array}{c} e\left(AggDBTagTagProof,g_{2}\right)==e\left(\prod_{i=0}^{C-1}H{\left(En\_IDTag_{i}\right)}^{ProofNonce_{i}}\times \upsilon^{DBTagProofMapValue},ppk\right)\;(22) \end{array}$

**PubProofsGen algorithm**: The PubProofsGen algorithm is used for implementing the operations of the PublicProofs-Generator component. It is used for generating public proofs. The algorithm takes *PubChall*_*j*_ and three sets of items, a set of the chosen data blocks, {*En*_*DB*_*i*_} and their associated tags, {*DBTag*_*i*_} and {*DBTagTag*_*i*_}, as the inputs. It outputs public proofs, i.e. *PubDBProof*_*j*_, *PubDBTagProof*_*j*_, and *DBTagTagProof*_*j*_, using Eq(11), Eq(12) and Eq(14), respectively. The details of PubProofsGen algorithm are given in Algorithm 6.

**DBProofTagGen algorithm**: The DBProofTagGen algorithm is used for implementing the operations of the DBProofTag-Generator component. It is used for generating a tag of *PubDBProof*. Thus, the algorithm takes a set of *PubDBProofs*, {*PubDBProof*_*j*_}, where *PubDBProof*_*j*_ is generated by *PCS*_*j*_ provider, and a set of *En*_*IDTags*, {*En*_*IDTag*_*i*_}, that are associated with the chosen data blocks in the *PubChall* as the inputs. Its outputs are the tag of *PubDBProof*, *BDProofTag*, using AS and an aggregated value of {*En*_*IDTag*_*i*_}, *AggEn*_*IDTag*, using Eq(8). The details of the DBProofTagGen algorithm are given in Algorithm 7.

**DBTagProofVer algorithm**: The DBTagProofVer algorithm is used for implementing the operations of the DBTagProof-Verification component. It is for verifying the *PubDBTagProof*. The algorithm takes *DBProofTag*, *PubDBTagProof*_*j*_, *AggEn*_*IDTag*, *PCSNonceTag*_*j*_, *AggProofNonceTag*, and the Paillier encryption key (*ppk*_*En*_), as the inputs. It outputs *DBTagProofVerResult*_*j*_ (0/1), the *DBTagProof* verification result, using Eq(15), Eq(16), Eq(17) and Eq(18). The details of the DBTagProofVer algorithm have been given in Algorithm 8.

**DBTagProofMap algorithm**: The DBTagProofMap algorithm is used for implementing the operations of the DBTagProof-Mapping component. It is used for computing a map value of *DBTagProof*_*j*_, *DBTagProofMapValue*_*j*_. The algorithm takes the following: *PubDBTagProof*_*j*_, *MappingSecertkey*, *PCSNonceTag*_*j*_ and {*ProofNonce*_*i*_} as the inputs. It outputs *DBTagProofMapValue*_*j*_ using Eq(19). The details of the DBTagProofMap algorithm have been given in Algorithm 9.

**DBTagTagProofVer algorithm**: The DBTagTagProofVer algorithm is used for implementing the operations of the DBTagTagProof-Verification component. The algorithm takes the five sets of items: {*DBTagProofVerResult*_*j*_}, {*PCSNonce*_*j*_}, {*En*_*IDTag*_*i*_}, {*ProofNonce*_*i*_}, and {*DBTagProofMapValue*_*j*_}, as the inputs. The algorithm outputs the *DBTagTagProof* verification result, *DBTagTagProofVerResult*_*j*_, using Eq(20), Eq(21) and Eq(22). The algorithm has been detailed in Algorithm 10.

**Fig 8:**
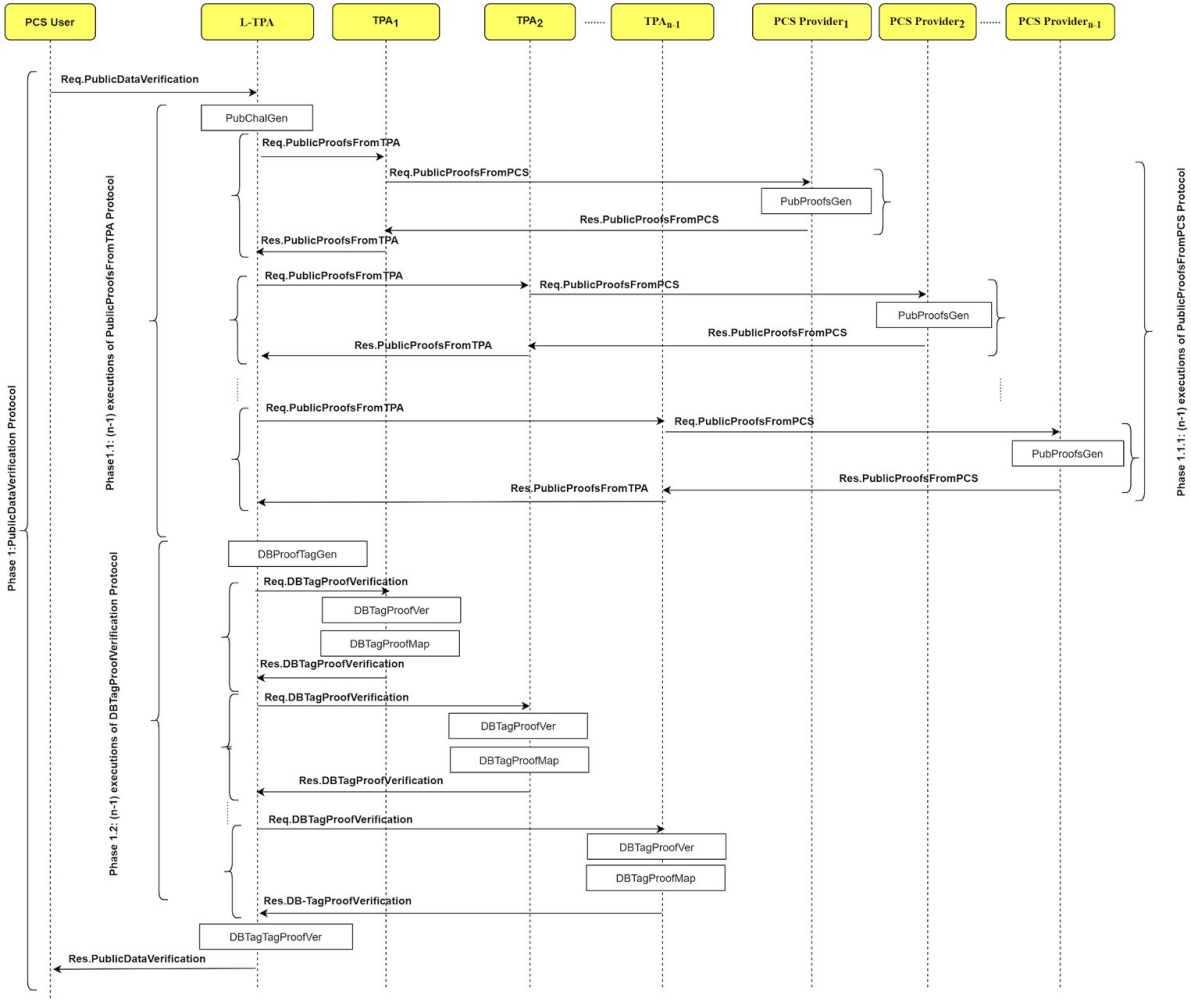
The LoA1DV protocol suite.

**Fig 9:**
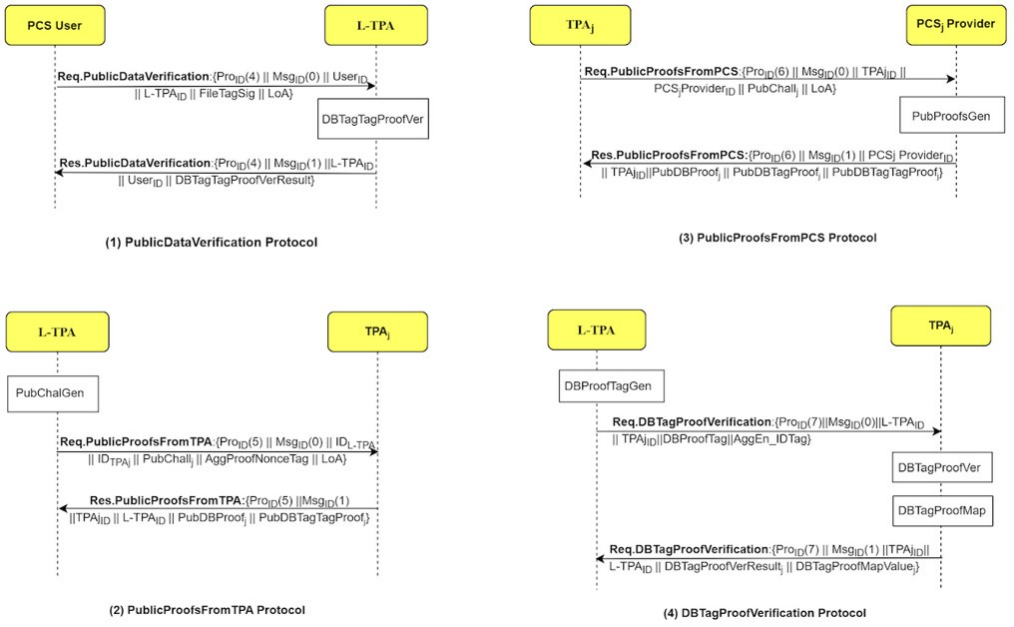
LoA1DV protocol: Sub-protocols and their messages.

The LoA1DV protocol is executed to allow the user to delegate the L-TPA to perform, control and manage the public verification among multiple non leader TPAs. The L-TPA forwards the result to the user. Fig 8 shows how the LoA1DV protocol is executed by illustrating its sub-protocols. The protocol consists of four sub-protocols, namely, PublicDataVerification, PublicProofsFromTPA, PublicProofsFromPCS, DBTagProofVerification. The sub-protocols and their messages are presented in Fig 9.

## LoA2 Data Verification (LoA2DV)

**Fig 10:**
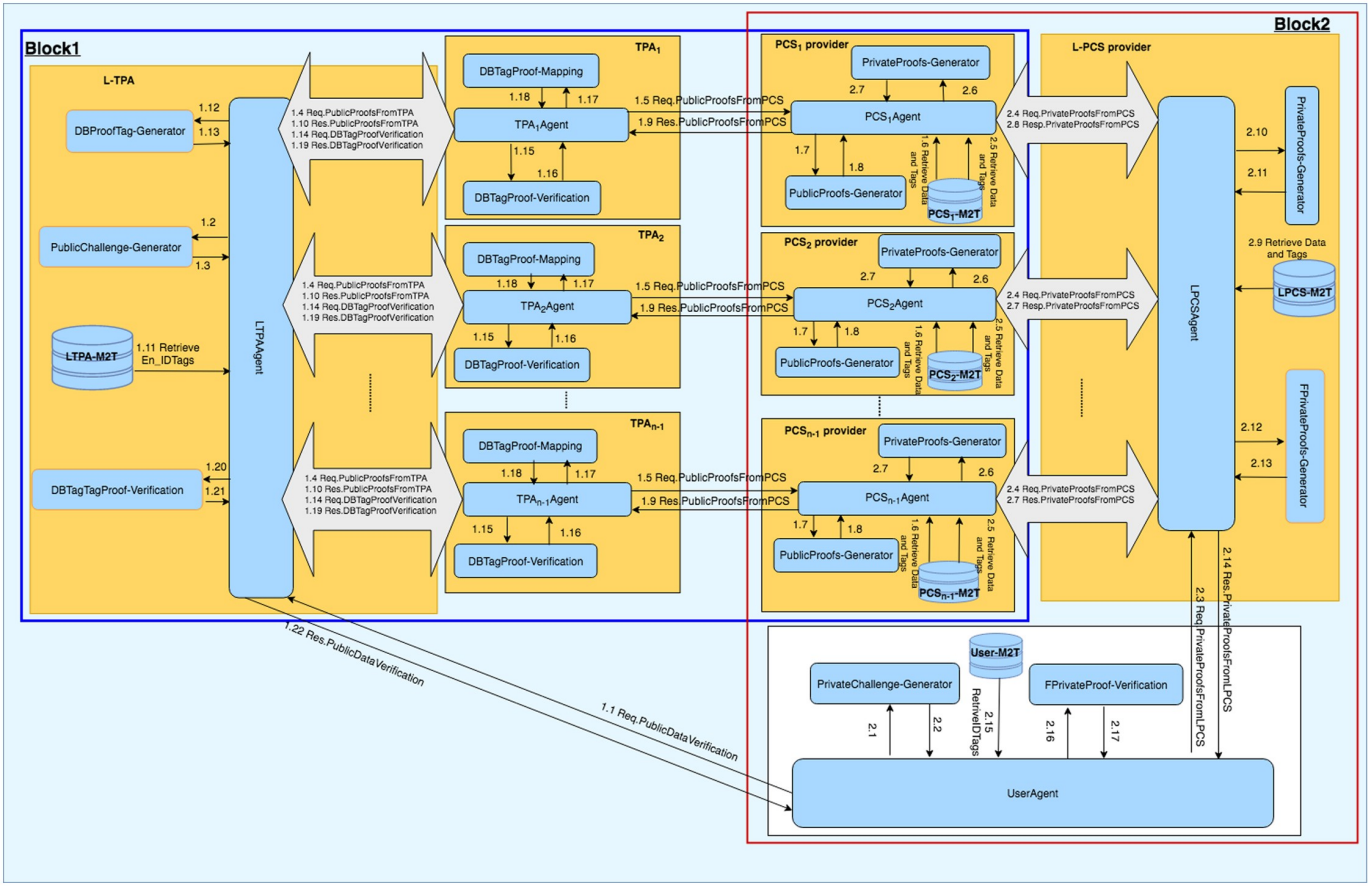
The LoA2DV architecture.

The LoA2DV is used for performing both verification types, public and private. The LoA2DV architecture, as shown in Fig 10 consists of two sub-blocks. The first one is identical to the LoA1DV architecture which contains the same entities and components used for performing public verification. The second block is used for performing private verification. The second sub-block involves three types of entities; the user, the leader provider and the non leader provider.

A detail of the algorithms used in the LoA2DV protocol. There are ten algorithms. The first six algorithms are identical to the ones of the LoA1DV, while the details of the latter four algorithms have been given below.

**PriChalGen algorithm**: The PriChalGen algorithm is used for implementing the operations of PrivateChallenge-Generator. It is used for the purpose of generating a challenge message for private verification. To generate the private proofs, it should determine which data blocks and their associated tags are used for their computing. The user and all of the TPAs ask the providers about the same data blocks. Thus the algorithm takes the indexes of the data blocks and their associated nonces that are in *PubChall*, {*I*_*i*_, *ProofNonce*_*i*_}, as the inputs. It outputs a private challenge (*PriChall*) and a random value is used as a nonce for the leader provider (*LPCSNonce*). The *PriChall* consists of the following items: (1) {*I*_*i*_}, (2) {*ProofNonce*_*i*_}, and (3) a tag of *LPCSNonce*, i.e. *LPCSNonceTag*, using Eq(23) (Tables 3 and 4 present all equations that are used in the LoA2DV). The details of the algorithm have been given in Algorithm 11.

**Table 4:** Math equations for private verification.

$\begin{array}{c} LPCSNonceTag=AS(LPCSNonce)\;(23) \end{array}$
$\begin{array}{c} PriDBProof_{j}=\sum_{i=0}^{C-1}\left(En\text{-}DB_{i}+ProofNonce_{i}\right)\;(24) \end{array}$
$\begin{array}{c} PriDBTagProof_{j}=\sum_{i=0}^{C-1}\left(DBTag_{i}+PCSNonceTag_{j}\right)\;(25) \end{array}$
$\begin{array}{c} FPriDBProof=\sum_{j=0}^{n-2}PriDBProof_{j}+PriDBProof_{L}\;(26) \end{array}$
$\begin{array}{c} FPriDBTagProof=\sum_{j=0}^{n-2}PriDBTagProof_{j}+PriDBTagProof_{L}\;(27) \end{array}$
$\begin{array}{c} AggProofNonce=\sum_{i=0}^{C-1}ProofNonce_{i}\;(28) \end{array}$
$\begin{array}{c} FPriDBTagProof_{1}=n\times AggIDTag+AS\left(FPriDBProof\right)+C\times \left(AggPCSNonceTag+LPCSNonceTag\right)\;(29) \end{array}$
$\begin{array}{c} FPriDBTagProof_{2}=FPriDBTagProof+n\times AggProofNonceTag\;(30) \end{array}$
$\begin{array}{c} FPriDBTagProof_{1}==FPriDBTagProof_{2}\;(31) \end{array}$

**PriProofsGen algorithm**: The PriProofsGen algorithm is used for implementing the operations of the PrivateProofs-Generator. It is used for generating private proofs. The algorithm takes two sets from the private challenge ({*I*_*i*_} and {*ProofNonce*_*i*_}), the requested data blocks, {*En*-*DB*_*i*_}, their associated tags ({*DBTag*_*i*_}) and *PCSNonceTag*_*j*_, as the inputs. It outputs the private proofs, *PriDBProof*_*j*_ and *PriDBTagProof*_*j*_, using Eq(24) and Eq(25), receptively. The algorithm has been detailed in Algorithm 12.

**FPriProofsGen algorithm**: The FPriProofsGen algorithm is used for implementing the operations of the FinalisedPrivateProofs-Generator component. It is used for generating the final private proofs. The algorithm takes the private proofs from all the non leader providers ({*PriDBProof*_*j*_}, and {*PriDBTagProof*_*j*_}, 0 ≤ *j* < *n* − 1), the private proofs from the leader provider (*PriDBProof*_*L*_ and *PriDBTagProof*_*L*_), as the inputs. The outputs are the final private proofs, *FPriDBProof* and *FPriDBTagProof*, using Eq(26) and Eq(27), respectively. The details of the algorithm have been given in Algorithm 13.

**FPriProofsVer algorithm**: The FPriProofsVer algorithm is used for implementing the operations of the FPriProof verification. It is used for verifying the correctness of the final private proofs. The algorithm takes five values: *FPriDBProof*, *FPriDBTagProof*, *LPCSNonceTag*, *AggPCSNonceTag* and *DBTagTagProofVerResult*, in addition to the set of *IDTags*, i.e. {*IDTag*_*i*_}, as the inputs. It outputs the final verification result, *FVerResult*, using Eq(28), Eq(29), Eq(30) and Eq31. The *FVerResult* is either positive or true (denoted as 1) which means that the integrity of the file is said to be assured. Otherwise, it is negative or false (0). This means that the integrity of the file is said to be unassured. The details of the FPriProofsVer algorithm have been given in Algorithm 14.

**Fig 11:**
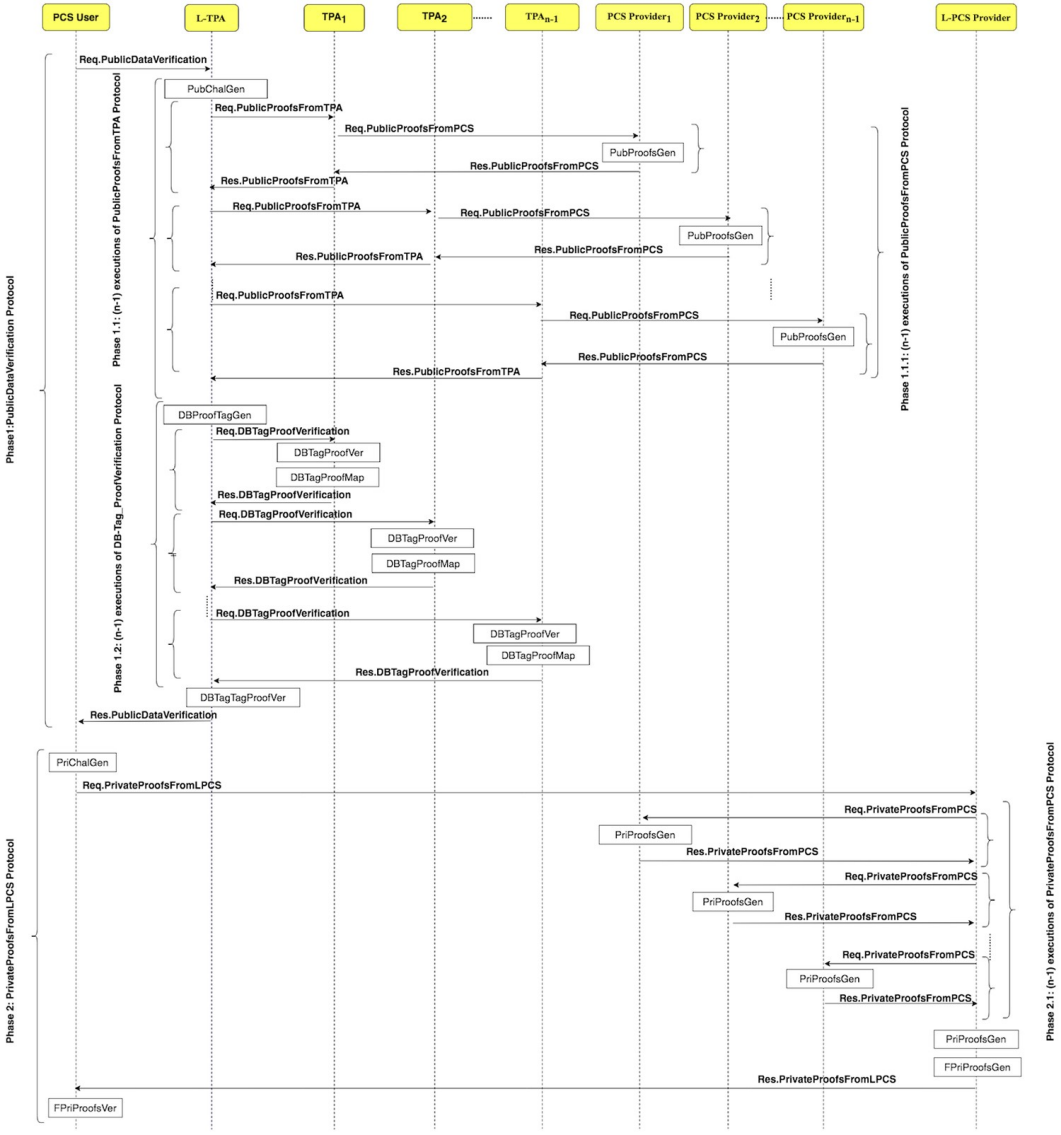
The LoA2DV protocol suite.

**Fig 12:**
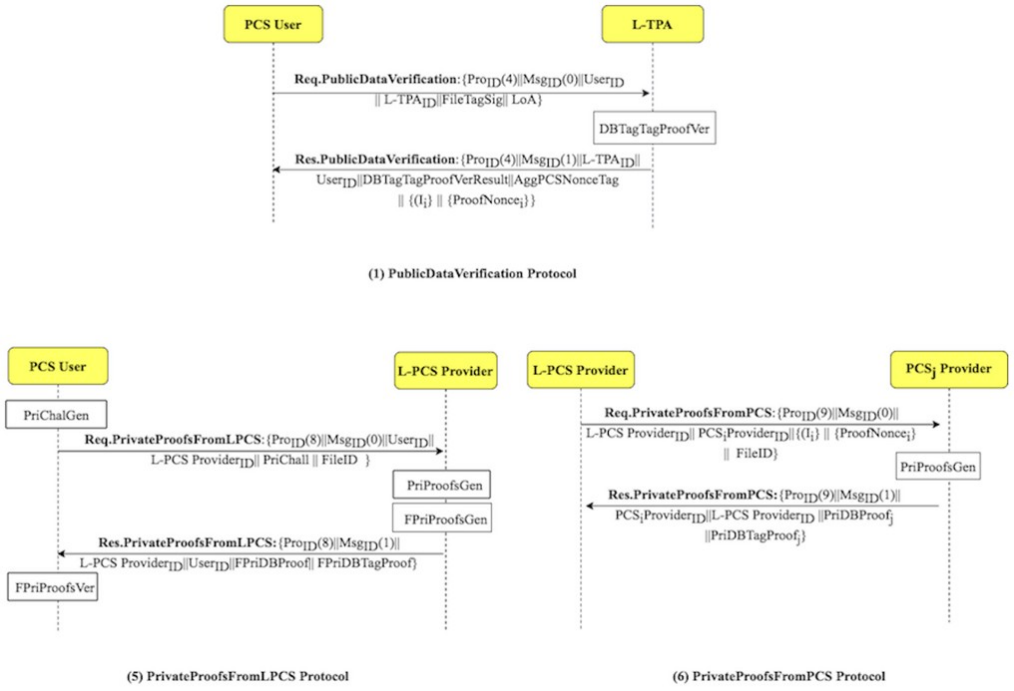
LoA2DV protocol: Sub-protocols and their messages.

The integrity checking of the data on PCSes is done using one of two integrity protection levels. The user can choose the integrity protection level, i.e. LoA1 or LoA2. In the LoA2DV, the LoA2 is chosen. The execution of the LoA2DV suite is performed in two phases. The first phase (Phase 1) is similar to the one in the LoA1DV protocol. It is used for performing the public verification. Meanwhile, in the second phase (Phase 2) is for the private verification. Fig 11 shows how the LoA2DV protocol is executed by illustrating its sub-protocols. Phase 2 consists of two sub-protocols, namely, PrivateProofsFromLPCS, and PrivateProofsFromPCS. The sub-protocols and their messages are shown in Fig 12.

## Data Updating (DU)

**Fig 13:**
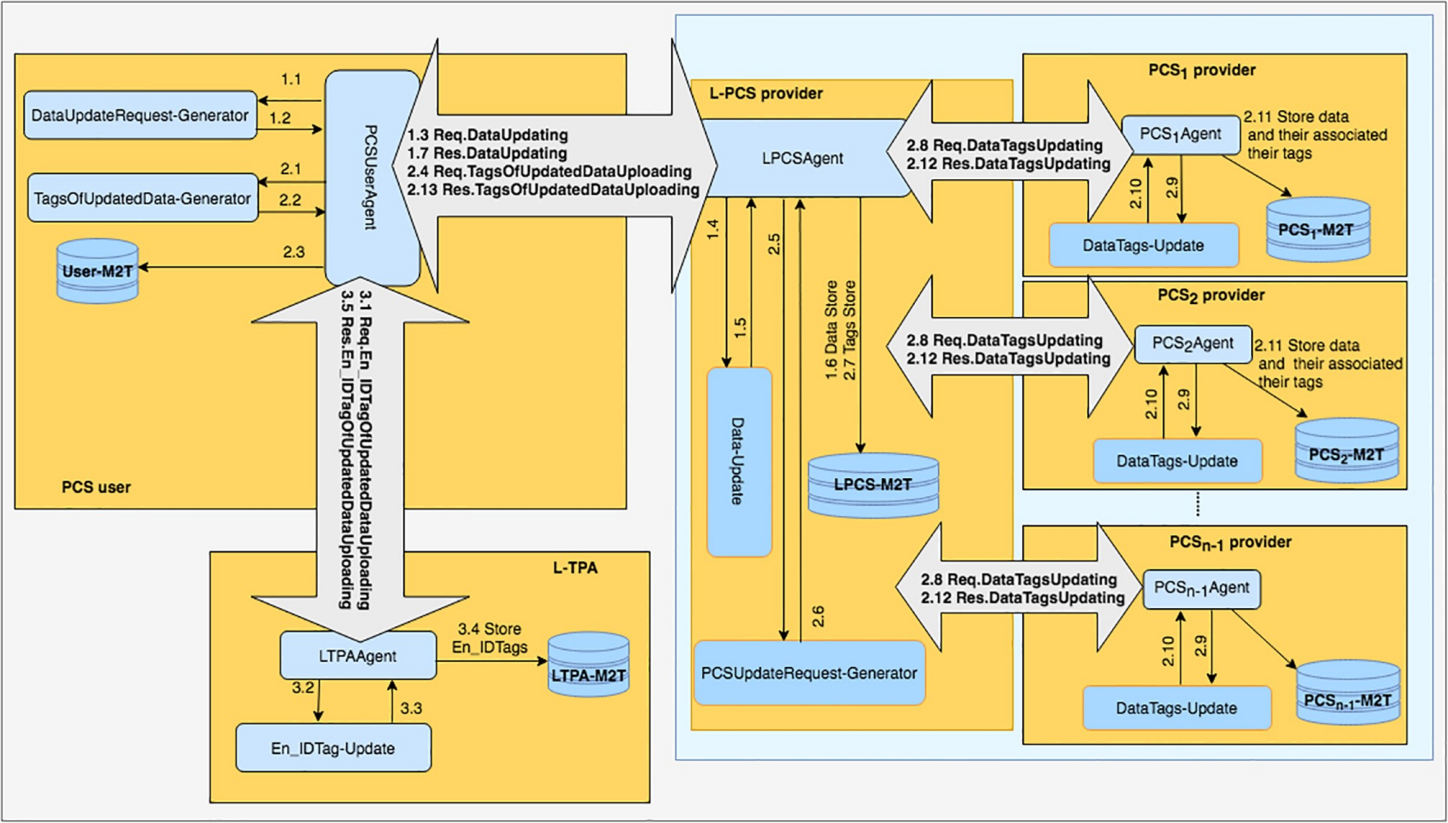
The DU architecture.

The architecture of the DU, as shown in Fig 13, includes four types of entities, the user, the leader provider, the non leader provider and the L-TPA. With the DU protocol, the user can update his/her outsourced data with associated tags on multiple PCSes.

A detail o the algorithms that are used in the DU protocol. There are six algorithms and their details are given below.

**DataUpdateReqGen**: The DataUpdateReqGen algorithm is used for implementing the operations of the DataUpdateRequestGenerator. It is used for generating an update request. The update request, *DataUpdateReq*, involves some of the information that providers needs for updating the outsourced data in the PCSes, e.g. the type of update operation, insert, modify or delete, a data block, a position of the data block among the data file, etc. Thus, the algorithm takes a data file identifier (*FileID*), a secret user key that is used for encryption and decryption of the data block (*sk*), a data block (*DB*), its position (Index), and operation type, *OpType*, (modify = 0, insert = 1, and delete = 2), as the inputs. It outputs *DataUpdateReq*. In the case of modification operation, *DB* is the old version of the data block that has position Index = *i* in the data file, i.e. *En*_*DB*_*i*_. This data block should first be decrypted using *sk*, modified to the new version and then encrypted using the same key, $En\_DB_{i}^{\prime }$. For the insertion operation, *DB* is a new data block that should be inserted after position, *i*, which is encrypted using *sk* to have *En*_*DB*_*i*+ 1_. Regarding the deletion case, *DB* is an empty value. To delete *En*_*DB*_*i*_, there are no encryption or decryption operations that need to be performed. The algorithm has been detailed in Algorithm 15.

**DataUpdate**: The DataUpdate is used for implementing the operations of the DataUpdate. It is used for executing an update request that has been received from the user. First, it checks to see if the updated data block is duplicated and/or linked to other existing blocks. Following this, it can update the M2T data structure. In the case where the request is a modification, it checks to see if the old version of the data block is linked to the files or not and if the new version data block is duplicated or not. Then one of the modification cases (Mod-Case_1, Mod-Case_2, Mod-Case_3, and Mod-Case_4) mentioned in Building Blocks Section can be performed. This is true of both the insertion and deletion requests. Based on the duplication or linking result of the new or deleted data block, one of the insertion or deletion cases (i.e. Ins-Case_1, Ins-Case_2, Del-Case_1, Del-Case_2) is performed for the process of updating. Thus the algorithm takes *DataUpdateReq*, as the input and outputs *UpdateResult*. The *UpdateResult* involves the duplication result and its associated ID in the case where its updated data block is duplicated (in the case of modification or insertion). The algorithm has been detailed in Algorithm 16.

**TagsOfUpdatedDataGen**: The TagsOfUpdatedDataGen is used for implementing the operations of the TagsOfUpdatedDataGenerator. It is used for generating tags for the updated data blocks, in the case of modification or insertion. Based on the *UpdateResult*, tags for the updated data block can be generated as well as updating User-M2T (by performing one of insert, modify and delete cases) and generating an update request for updating LTPA-M2T. Thus the algorithm takes *DataUpdateReq*, *UpdateResult*, the user ID (*User*_*ID*_), the user BLS private key (*x*), the random number (*υ*), the Paillier encryption key (*ppk*_*ID*_), and the total number of data blocks in the data file (*K*), as the inputs. It outputs *UpdatedTags*, *En*_*IDTagUpdateReq*. The *UpdatedTags* contains the *DBTag*_*i*_ and *DBTagTag*_*i*_ of the updated data block or a value 1 in the case of having the duplicated data block, while the *En*_*IDTagUpdateReq* involves the *En*_*IDTag*_*i*_ for updated data block, the operation type, the ID of *En*_*IDTag*_*i*_ in the case where the updated data block is duplicated and whether one of the existing *En*_*IDTags* should be deleted or not. The algorithm has been detailed in Algorithm 17.

**PCSUpdateReqGen**: The PCSUpdateReqGen is used for implementing the operations of the PCSUpdateRequest-Generator. It is used for generating an update request that is sent to the non leader providers to update their M2Ts. The algorithm takes *DataUpdateReq*, *UpdateResult* and *UpdatedTags* as the inputs, and it outputs *PCSUpdateReq*. The *PCSUpdateReq* involves information, e.g., the operation type, the ID of *DB* in the case of the updated data block is duplicated, or the data block value and its associated tags, in the case of the updated data block is non-duplicated. The algorithm has been detailed in Algorithm 18.

**DataTagsUpdate**: The DataTagsUpdate is used for implementing the operations of the in DataTags-Update. It is used for updating the non leader provider’s M2T. The algorithm takes *PCSUpdateReq* as the input, and it outputs *ACK*. Based on the information in *PCSUpdateReq*, one of the modification, insertion and deletion cases is performed. The algorithm has been detailed in Algorithm 19.

**En_IDTagUpdate**: The En_IDTagUpdate is used for implementing the operations of the En_IDTag-Update. It is used for updating the LTPA’s M2T. The algorithm takes *En*_*IDTagUpdateReq* as the input, and it outputs *ACK*. Based on the information in *En*_*IDTagUpdateReq*, one case of the modification, insertion and deletion cases is performed. The algorithm has been detailed in Algorithm 20.

**Fig 14:**
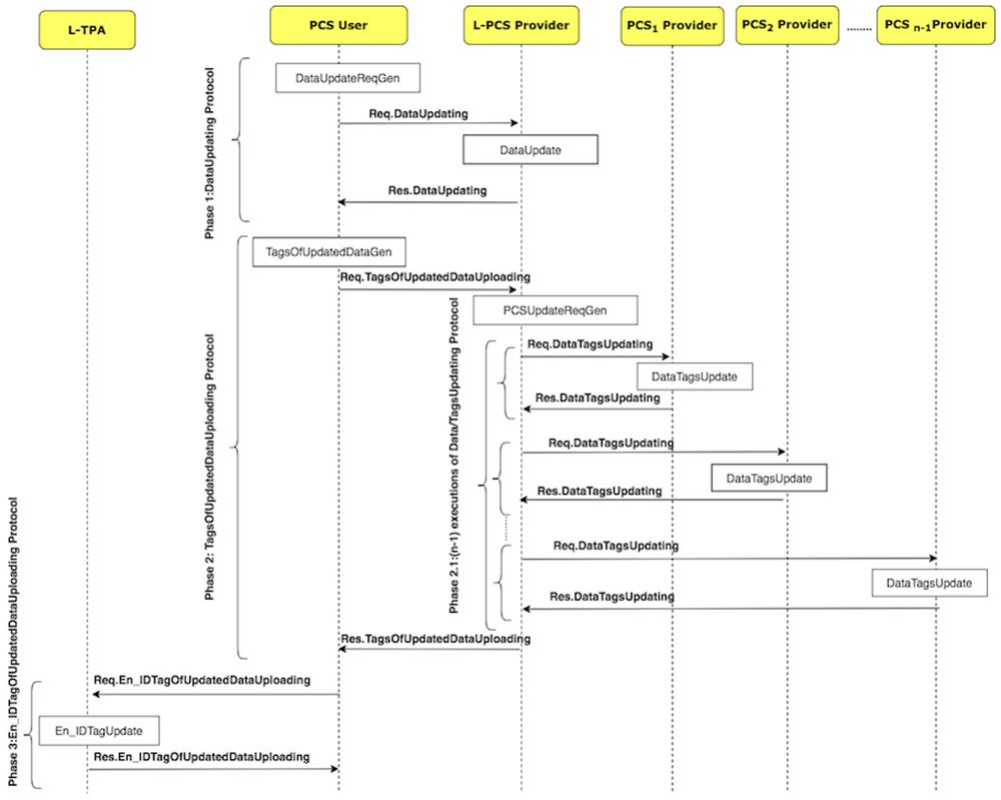
The DU protocol suite.

**Fig 15:**
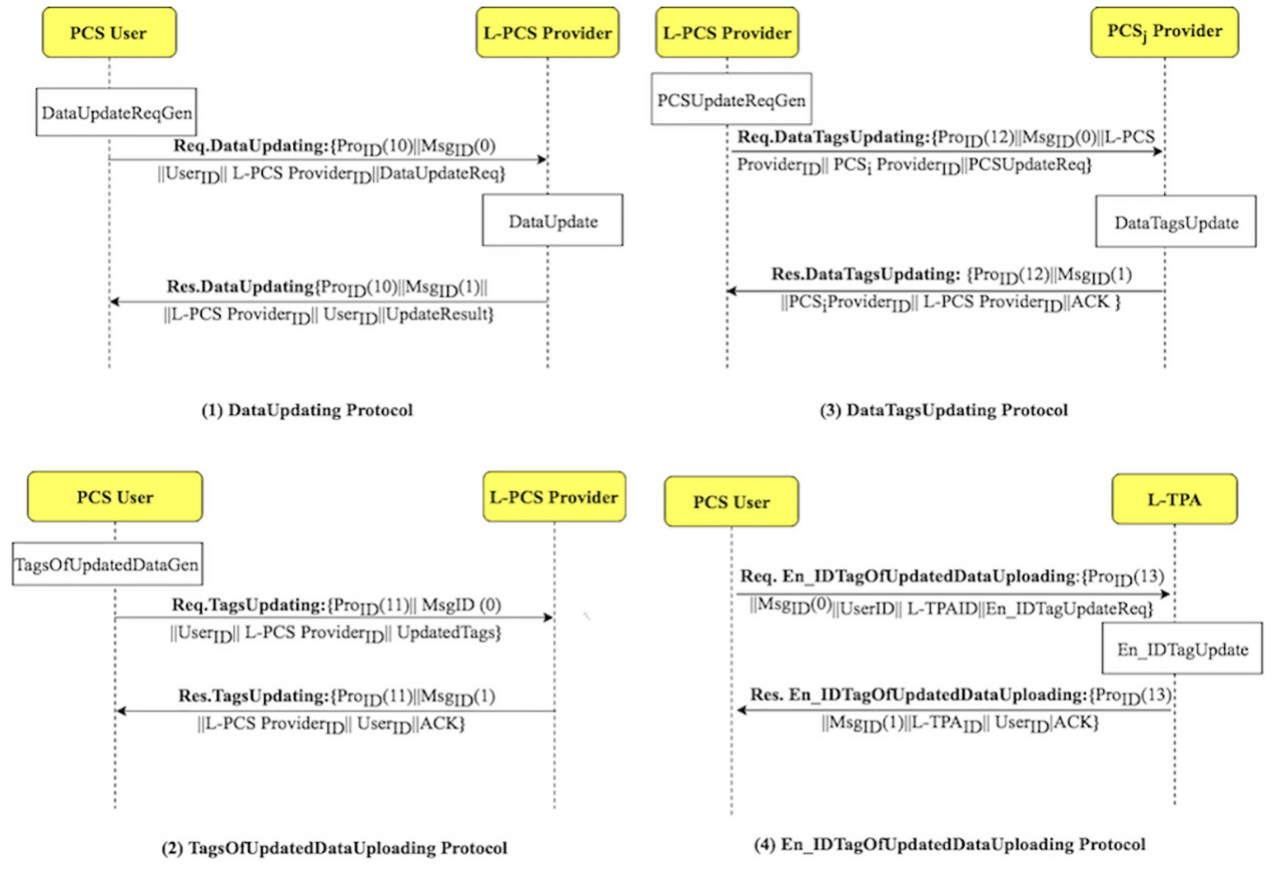
DU protocol: Sub-protocols and their messages.

Fig 14 shows how the DU protocol is executed by illustrating its sub-protocols. The protocol consists of four sub-protocols, namely, DataUpdating, TagsOfUpdatedDataUploading, DataTagsUpdating, En_IDTagOfUpdatedDataUploading. The sub-protocols and their messages are shown in Fig 15.

## Correctness and security of the DIA-MTTP verification protocols

In this section, we analyse the correctness and the security of DIA-MTTP verification protocols, i.e. LoA1DV and LoA2DV. The security analysis makes use of the security requirements specified in Requirement Specification Section.

### Correctness of LoA1DV and LoA2DV

**Theorem 1**: Given the proofs (i.e. DBProof, DBTagProof (public and private) and DBTagTagProof), the verifier (i.e. the user or the TPAs) can verify the integrity of the data file using LoA1DV or LoA2DV protocol.

**Proof**: Proving the correctness of our protocols, i.e. LoA1DV and LoA2DV, is equivalent to proving the correctness of following equations, Eq(18), Eq(22) and Eq(31). Based on the property of algebraic signature [50], the homomorphic addition property in Paillier algorithm [49] and the bilinear property [51], all the three equations hold (see S4 File).

$$\begin{array}{c} AS\left(DB_{1}\right)+AS\left(DB_{2}\right)=AS\left(DB_{1}+DB_{2}\right) \end{array}$$ (32)

$$\begin{array}{c} E\left(DB_{1}+DB_{2}\right)=E\left(DB_{1}\right)\times E\left(DB_{2}\right) \end{array}$$ (33)

$$\begin{array}{c} e\left(W^{a},R\right)=e\left(W,R^{a}\right)\;\text{for}\;W\in G_{1}\text{,}\;R\in G_{2}\;\text{and}\;a\in Z_{p} \end{array}$$ (34)

### Data confidentially preservation

In DIA-MTTP, the providers and TPAs are authorised to manage and verify the integrity of the data. However, they should not have the privilege to access the content of the data. Taking into account that: (1) the use of the TOD method, (2) the assumption that the cryptographic keys are securely generated and stored (i.e. A4), and (3) the assumption that the communication channels that connect the entities in the DIA-MTTP are secure and authenticated (i.e. A1). Our two protocols, LoA1DV and LoA2DV, can provide data confidentiality preservation. The data blocks in a data file are encrypted with a symmetric key, and this key is only known to the user (the data owner) before being sent to the PCSes. Without this key, any other entities, including the providers or TPAs, are not able to access the plain-text data. Additionally, the encrypted data are used in tags generation. When verifying the integrity of the data file, the provider and the verifier (TPA) do not have access to the plain-text of the data file, or any of its data blocks.

### Resistance to cheating attacks by PCS providers

The providers can launch attacks, forgery, replace and replay in order to cheat a verifier (a user or TPA). This is to hide the fact that the data integrity was compromised to allow it to pass verification. We need to prove that our verification protocols are secure against these attacks, where providers cannot generate proofs without querying the real data or where they cannot modify the data and the associated tags without detection. In other words, the providers can compute proofs related to passing the data integrity verification only if all of the requested data and their associated tags are correctly stored in PCSes. In the following, we discuss these attacks and how our protocols can satisfy the requirement (S1).

In a forgery attack, the providers try to forge proofs by forging the tags used by and that have been generated by a user, who in this case is the data owner. Taking into account that: (1) use the TOD method, and (2) assuming that the cryptographic keys are securely generated and stored (A4), therefore, our two protocols, LoA1DV and LoA2DV, can resist the forgery attack. The TOD method can generate forgery resistant tags, private tags ({*DBTag*_*i*_}) and public tags ({*DBTagTag*_*i*_}) [47].

For the data verification, a provider can use data blocks and their associated tags when computing public or private proofs that are different from those that user or L-TPA has determined in a challenge message or use an old version of the data blocks and their associated tags. Thus, they launch the replace attack. Taking into account that: (1) use the TOD method, and (2) the same data blocks are requested from multiple PCSes in each verification time, (3) use the distributed data structures, i.e. M2Ts. Our two protocols, LoA1DV and LoA2DV, can provide resistance against replace attacks.

In this attack, the provider can exploit the collisions between the outsourced tags. The TOD method can generate collision resistant tags that are either private, {*DBTag*_*i*_}, or public, {*DBTagTag*_*i*_} [47]. The identifier of the tags, i.e. *IDTags* or their encrypted forms, i.e. *En*_*IDTags* for each data block, are used for collision resistance. This is where each data block has a unique *IDTags* and *En*_*IDTags*. Furthermore, in both protocols, all TPAs request the same data blocks from all providers each verification time. This means that they should be identical. The L-TPA compares all of these values when generating a tag for the *PubDBProofs*, that have been received from the TPAs. If the data blocks are different in the *PubDBProofs* then consequently, different *PubDBProofs* are received.

In the DIA-MTTP, each entity has its data structure (User-M2T, PCS-M2T and LTPA-M2T). In each update, these data structures should be updated. As mentioned in Section, the linkages between the data blocks of the data files and their associated *IDTags* or *En*_*IDTags* are updated, in the case of duplicated data blocks. On the other hand, if updated data blocks are not duplicated, new tags should be generated. Each entity, that of the user, the L-TPA and the provider, should update its associated M2T by storing the tags in the NonDuplicatedDB/Tag table and linking the data blocks of the data file in the Linker table with these tags. All entities can track the update operation, not only the provider. This can help to detect if the provider is cheating.

A replay attack can be launched by sending previously generated proofs. In other words, if the TPA re-requests the same data, then the provider can send the old proofs that have been generated in any previous challenge, without real access to the data and their associated tags. Taking account of using: (1) the random sample strategy, and (2) nonces (i.e. {*ProofNonce*_*i*_} and {*PCSNonce*_*j*_}). This is so then the attack can be detected in the process of the data verification. Our two protocols, LoA1DV and LoA2DV, can be secured against the replay attack. The L-TPA chooses the data blocks randomly each data integrity verification time. Consequently, there is a high number of combinations that can be selected before re-choosing the same data blocks. On the other hand, in the case where the L-TPA re-requests the same data blocks, the use of {*ProofNonce*_*i*_} and {*PCSNonce*_*j*_} can help to prevent the attack. The nonces are different for each data verification time. The L-TPA chooses these nonces and all providers should use refreshed ones and include them in the proof generation as shown in Eq(11), Eq(12), Eq(14), Eq(24) and Eq(25).

### Resistance to cheating attacks by TPAs

TPAs can launch some types of attacks, i.e. collusion attacks and frame attacks to cheat a user. The TPAs generate an unreliable verification result. In the following, we need to prove that our protocols are secure against these attacks to satisfy the requirement S2.

In a collusion attack, the TPA works with its associated provider to commit fraud. Even if the data integrity that is comprised, the TPA lies and sends the data is correctly stored in the PCS without any integrity violations. Thus, the data in the PCS has been unauthorised altered and lost without detection.

Taking into account that: (1) the use of the collaborative verification approach, (2) the use of nonces ({*ProofNonce*_*i*_}) in the verification, (3) the use of dual verification, public and private, and (4) the assumption of the keys are securely shared (A2), consequently, our two protocols (LoA1DV and LoA2DV) can be secured against collusion attacks.

In the LoA1DV protocol, the process of checking the correctness of the proof are distributed between multiple TPAs (the non leader TPAs) and the L-TPA. In other words, the data verification result is not approved by one entity (the L-TPA or TPAs). Public verification consists of two sub-verifications; *PubDBTagProof* verification and *PubDBTagTagProof* verification. Each TPA verifies its associated *PubDBTagProof*, while the L-TPA verifies all of the *PubDBTagTagProofs*. The TPA cannot verify *PubDBTagProof* without having the *DBProofTag*. This value only can be generated by the L-TPA. The L-TPA computes the *DBProofTag* using the algebraic signature that its parameters system only shared with the L-TPA. The same is true when verifying the *PubDBTagTagProof*. The L-TPA cannot verify it without having the *DBTagProofMapValue*. This value only can be generated using a key-based hash function, DBTagProofMap algorithm. The key is used in the algorithm and it can only be shared with the TPA, thus the L-TPA cannot generate this value.

The TPA may try to defraud the L-TPA by sending a positive result for the *DBTagProof* verification regardless of whether the verification holds or not or an old generated *DBTagProofMapValue*. However, the TPA should send the *DBTagProofMapValue*, along with the result verification of *PubDBTagProof* to the L-TPA. Furthermore, the TPA should use {*ProofNonce*_*i*_} when generating the *DBTagProofMapValue* where they are different {*ProofNonce*_*i*_} each verification time. This can help the L-TPA to detect replay attacks from the TPA.

The LoA2DV protocol can be more resistant to a collusion attack compared to the LoA1DV protocol. In addition to the above mentioned remarks, the user performs the private verification along with public verification using the TPAs in the LoA2DV. In the private verification, the user requests the same data blocks and their associated tags from the providers that are used in computing the public proofs.

The frame attack may be launched by the TPA to destroy the provider’s reputation even if the data integrity at the PCS is preserved. The data at the PCS has not been changed, but the TPA lies and sends the proof of the provider failing the verification as in Eq(18) or Eq(22).

Taking account of using: (1) the collaborative verification approach, (2) nonces ({*PCSNonce*_*j*_}), and (3) the dual verification, the LoA1DV and LoA2DV protocols can be secured against frame attacks.

In the LoA1DV protocol, as mentioned above, the data verification is approved using multiple entities (L-TPA and TPAs). Therefore, it can help to have a low probability of an attack occurring. In the LoA2DV protocol, in addition to the collaborative verification approach, the dual verification and nonces ({*PCSNonce*_*j*_}) are used. These can help the user to detect a frame attack and they can make the LoA2DV protocol more frame attack resistant. {*PCSNonce*_*j*_} are generated by the L-TPA, and each TPA should send *PCSNonce*_*j*_ to its associated provider, which are different for each separate verification time. The user does not send the nonces, and the providers should include the receiving ones in their proofs (both private and public). Furthermore, the user has a tag of an aggregated value of the nonces to prevent their forgery. These nonces can thus be used as evidence that the TPAs have communicated with their associated providers and sent fresh nonces.

### Resistance to cheating attacks by PCS users

Dishonest users may repudiate the generation of tags or updating the data at PCSes and they can also refuse a data verification result that has been received from the L-TPA in an attempt to maximise their benefits, such as by trying to discredit the providers and/or TPAs to seek financial advantages.

Taking into account that use: (1) the TOD method, and (2) the distributed data structures, the LoA1DV and LoA2DV protocols can be secured against the cheating attacks conducted by a user. The TOD method uses a BLS signature in the tag generation, thus, it can provide a non-repudiation property. If this verification holds when using the user’s public key as in Eq(22), then the user cannot deny that he does not sign the *DBTagTags* further. Furthermore, as {*DBTag*_*i*_} is used when generating and verifying *DBTagTags*, thus, the user cannot falsely deny that he has generated the tags, i.e. {*DBTag*_*i*_}, too.

In a dynamic data case, the user may cheat by refusing to update the data. However, each entity in the DIA-MTTP system has its M2T for tracking the update operations. Furthermore, for each updated item of data, the user generates tags for the data using the TOD method. Therefore, the user cannot repudiate the updated data.

## Performance evaluation of the DIA-MTTP

This section first analyses detection probability and then evaluates the overhead cost of the DIA-MTTP. The overhead cost evaluation for the DIA-MTTP was performed by measuring introduced costs in terms of computation and communication according to each functional block (i.e., D3U, LoA1DV, LoA2DV and DU) and the storage cost of the entities of the DIA-MTTP.

We have also carried out experiments to investigate the performance costs of DIA-MTTP, the computational costs. The experiments were implemented using a desktop computer running a system with Intel Core i5 at 2.4 GHz and 4GB RAM (a single-machine set-up). Furthermore, the experiment was based on a single user data request to verify one data file. The software used to implement the DIA-MTTP was Java Platform, Standard Edition (Java SE) [52]. To implement the cryptographic primitives required in the DIA-MTTP such as, a secure random number generator, a hash function (e.g. SHA256), and digital signatures (e.g. RSA and BLS), Java Cryptography Extension (JCE) [53] and Java Pairing-Based Cryptography (JPBC) [54] were used. MySQL [55] was used to implement the M2T data structure.

We conducted two experiments. In the first experiment (Exp1), we measured the computational cost by calculating the time of the execution of the cryptographic operations, e.g. multiplication in *G*_1_, hashing, etc. Table 5 lists the basic operations: their symbols and timing measurement. In the second experiment (Exp2), we measured the computational cost by calculating the time that it took each entity from receiving a request message up until before a response was sent.

**Table 5:** Cryptographic operations and their computational time (in seconds).

Notation	Description	Timing measurements
$Mult_{G_{1}}$	Multiplication in *G*_1_	6.2 × 10^−4^
$EXP_{G_{1}}$	Exponentiation in *G*_1_	4.7 × 10^−3^
$Pair_{G_{1},G_{2}}$	Bilinear pairing e(x, y), x ∈ *G*_1_, y ∈ *G*_2_	1.2 × 10^−2^
*H*1	Cryptographic hashing, i.e. *H*1()	2.5 × 10^−4^
$H_{G_{1}}$	Hashing to *G*_1_, i.e. *H*()	2.9 × 10^−3^
*Add*_*Zp*_	Addition in Zp	2.1 × 10^−5^
*Mult*_*Zp*_	Multiplication in Zp	3.8 × 10^−5^
*Exp*_*Zp*_	Exponentiation in Zp	6.9 × 10^−4^
$Mult_{Z_{n^{2}}}$	Multiplication in *Z*_*n*^2^_	2.3 × 10^−5^
$Exp_{Z_{n^{2}}}$	Exponentiation in *Z*_*n*^2^_	1 × 10^−2^
*Add*_*AS*_	Addition in *GF*(2^*m*^)	2 × 10^−6^
*AS*-*G*	Cost of *GF*(2^*m*^)	6.8 × 10^−5^

### Detection probability

Both of the data verification protocols, LoA1DV and LoA2DV, have been constructed as a random sampling strategy from efficient and security perspectives. The random sampling strategy is used to reduce the workload of the provider in terms of a proof generation and communication cost, in addition to preventing the provider from cheating the verifier, i.e. the user or TPAs, using replay attacks. In the random sampling technique, the data file is divided into multiple data blocks (*K*) and a number of blocks (*C*) are chosen randomly by the verifier to perform data verification. We analyse the probability of misbehaviour in the detection of our protocols, LoA1DV and LoA2DV data verification, based on the blocks sampling.

Suppose the provider tries to hide the modification of *MD* blocks out of the *K* outsourced blocks. The probability of corrupted blocks at *PCS*_*j*_ is equal to $P_{MD_{PCS_{j}}}=MD/K$. Let *V* indicate the number of blocks chosen by the verifier matching the blocks modified by the provider. According to [9], we computed the probability that at least one of the blocks picked by the verifier matches one of the modified blocks at *PCS*_*j*_, which is $P_{V_{PCS_{j}}}\left(V\geq 1\right)$, as follows:$P_{V_{PCS_{j}}}\left(V\geq 1\right)=1-{\left(1-P_{MD_{PCS_{j}}}\right)}^{C}$. The probability of misbehaviour detection at *n* PCSes, *P*_*DIA*−*MTTP*_, can be calculated as follows: $P_{DIA-MTTP}=\prod_{j=0}^{n-1}P_{V_{PCS_{j}}}=1-\sum_{j=0}^{n-1}{\left(1-P_{MD_{PCS_{j}}}\right)}^{C}$.

**Fig 16:**
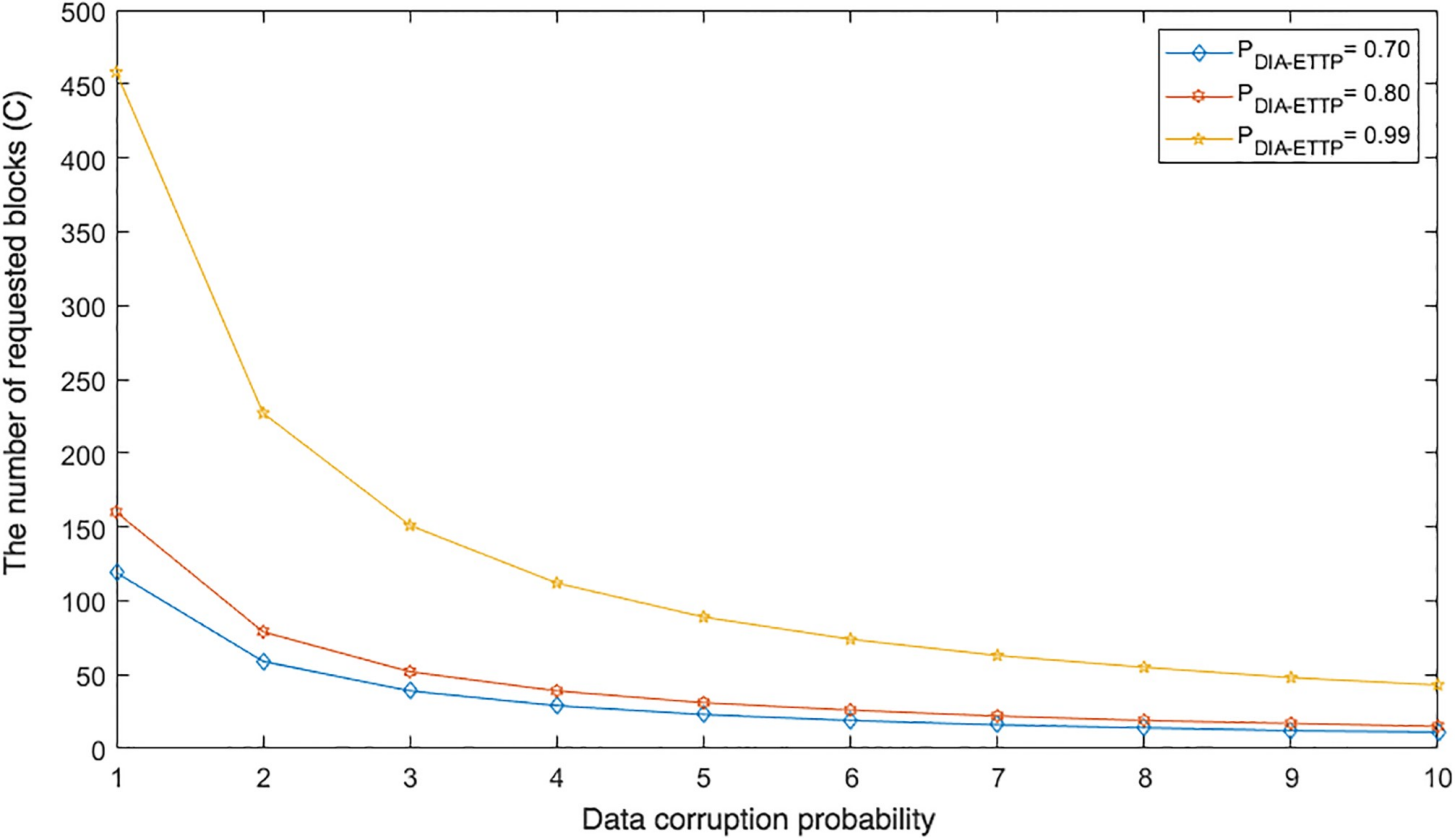
Data corruption probability vs the number of requested blocks under different detection probabilities.

Suppose the verifier divides the data file into 1000 blocks and outsources them to 10 PCSes. Fig 16 shows the required number of picked data blocks for verification, *C*, that are used to detect different numbers of modified blocks (*MD*) when the probability of misbehaviour detection for one provider is selected from a set of *P*_*DIA*−*MTTP*_ = {0.70, 0.80, 0.99}. For example, if each provider modifies 1% of the outsourced blocks, then the verifier needs to randomly select 458 blocks from each provider as a challenge to achieve a probability of at least 0.99. By increasing the number of modified blocks, the lowest number of challenge blocks possible is required to achieve such a probability of detection.

### Introduced cost by the D3U

In the DIA-MTTP evaluation, we assume that there is a data file (DF) with *K* data blocks. After cutting out the redundant data blocks (we only kept one copy of each data block), the number of data blocks in a data file can be reduced to *d*1 (*d*1 ≤ *K*) by applying D3L1 among the data blocks of the file itself, and to *d*2 (*d*2 ≤ *d*1) by D3L2 applied to the data blocks of all of the outsourced files in the PCSes. In the following, the computational and communication costs of the D3U that incurred at each entity are presented in details.

The computational cost incurred by the user in the D3U comes from executing two sub protocols, i.e. L2DataDeduplication and TagsUploading. In the L2DataDeduplication protocol, the user pre-possesses his data and generates *d*1 non-duplicated and encrypted data blocks (DFProCost). In the TagsUploading protocol, the user computes *d*2 tags for the non-duplicated data blocks (DFTagGenCost).

In prepossessing the data file, the highest incurred cost is due to the encryption. The user divides the data file into multiple data blocks (*K*) and then encrypts them using the LiSHE scheme. The encryption cost can be reduced, by applying the D3L1 among the data blocks, from *K* to *d*1. Instead of encrypting *K* data blocks, it can only encrypt *d*1 data blocks. In other words, the cost of the data block encryption can be reduced by (*K* − *d*1).

Upon receiving the duplication result of D3L2 from the leader provider, the user can generate the tags for the non-duplicated data blocks. Using the TOD method, each data block is associated with a set of four tags, *IDTag*, *DBTag*, *DBTagTag* and *En*_*IDTag*. The number of tags (i.e. sets) can be reduced by applying the D3L2 to *d*2. It is clear that the DFTagGenCost is affected by the two levels of data deduplication (D3L1 and D3L2). This is where the tag number reduced by ((*K*—*d*1) + (*d*1—*d*2)).

Table 6 compares the number of encrypted data blocks and their associated tags with/without applying deduplication. It shows that using block-level deduplication can lead to reducing the DFProCost as the number of identical data blocks increases by reducing the total number of data blocks in the file. In the file-level deduplication based and non-deduplication based works, the DFProCost remains constant regardless of the number of identical data blocks. For example, in the case of uploading one data file that has 1000 data blocks where the redundant data blocks percentage is 50%, the user only needs to encrypt 501 data blocks using DIA-MTTP instead of 1000 data blocks using the other works, i.e. file-level deduplication based and non-deduplication based works. The table emphasises that the DFProCost complexity is based on the redundant data block percentages (D3L1), O(*d*1). On the other hand, the DFTagGenCost complexity is based on redundant data blocks percentages in D3L2, O(*d*2). For example, for uploading one data file and the redundant data blocks parentage 20% in both levels of data deduplication (i.e. D3L1 and D3L2), the user only needs to generate tags for 641 data blocks instead of 1000 data blocks. When the number of the excluded data blocks increased under the two levels of deduplication, the number of tags decreased, and consequently, the tag generation cost can be reduced.

**Table 6:** Number of encrypted data blocks and their associated tags: With/without the data deduplication approach.

3D3L1	Encrypted DBs Number			D3L2	Tags Number		
	DIA-MTTP	DIA work*	DIA work**		DIA-MTTP	DIA work*	DIA work**
0%	1000	1000	1000	0%	1000	1000	1000
20%	801	1000	1000	20%	641	1000	1000
50%	501	1000	1000	50%	250	1000	1000
70%	301	1000	1000	70%	90	1000	1000

(* DIAs do not support data deduplication, ** DIAs support file-level deduplication)

For each block size, from 2KB to 1024KB, we firstly fragmented the data file into multiple blocks and generated their tags. We then calculated the time taken for the tag generation of the data file by multiplying the total number of blocks by the time taken for the generation of one tag for one data block. As illustrated in Table 8, the computational cost of the tag generation for the same data file decreases almost linearly with the increasing block size. The number of data blocks decreases accordingly. We tested the DFTagGenCost of 1 × 10^6^ KB and 1 × 10^7^ KB data files. We found that, under the same block size, the cost of a 1 × 10^6^ KB data file is nearly ten times the cost of a 1 × 10^7^ KB data file. This also demonstrates that the number of data blocks dominates the computational cost of the tag generation. A small fragmentation refers to a large number of data blocks. Therefore, it is better to choose a big block size for data fragmentation to an outsourced largesize data file. The details of complexities of computational and communication cost in DIA-MTTP are given in S5 File.

**Fig 17:**
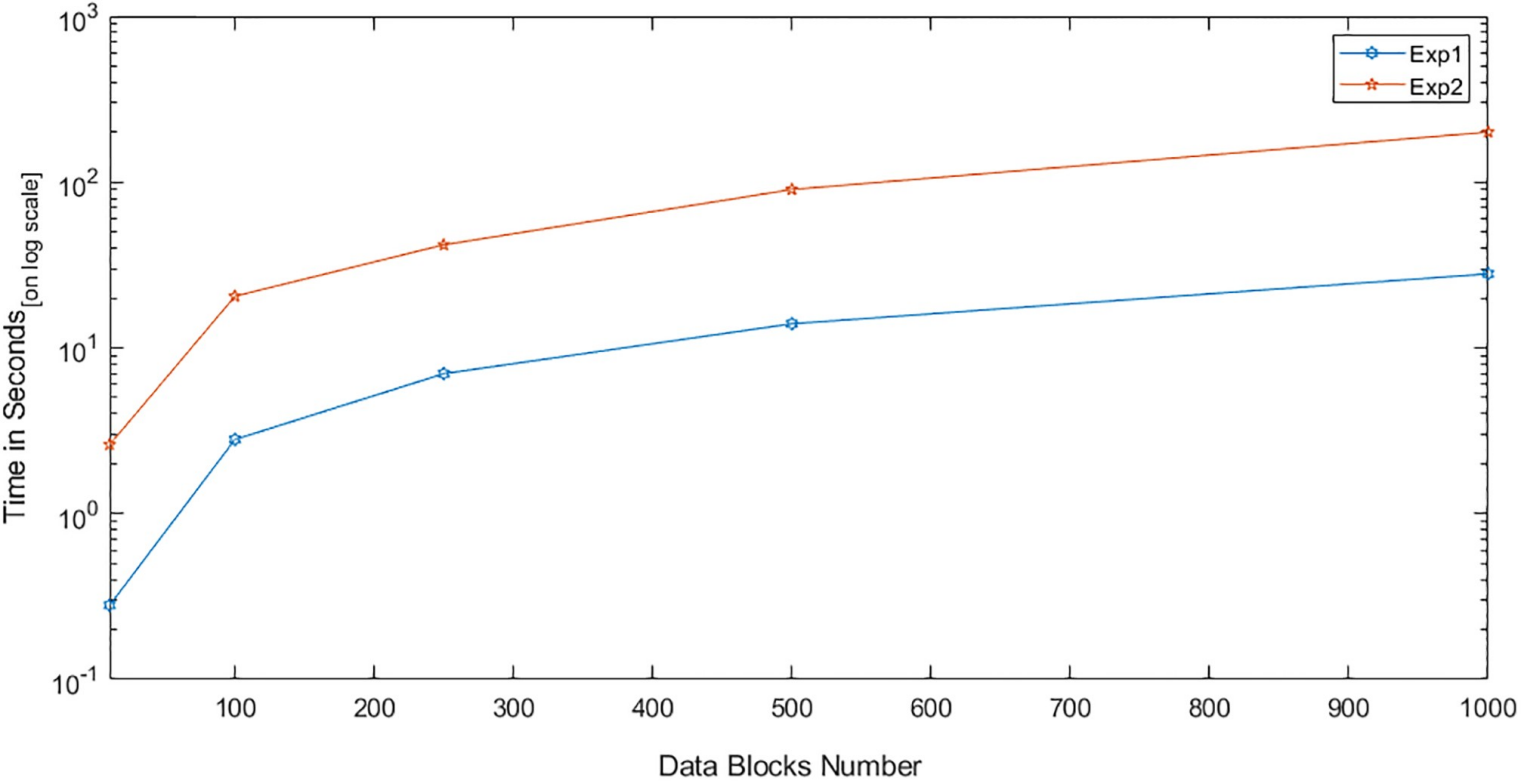
Computational cost of the user in the D3U vs the number of data blocks.

**Fig 18:**
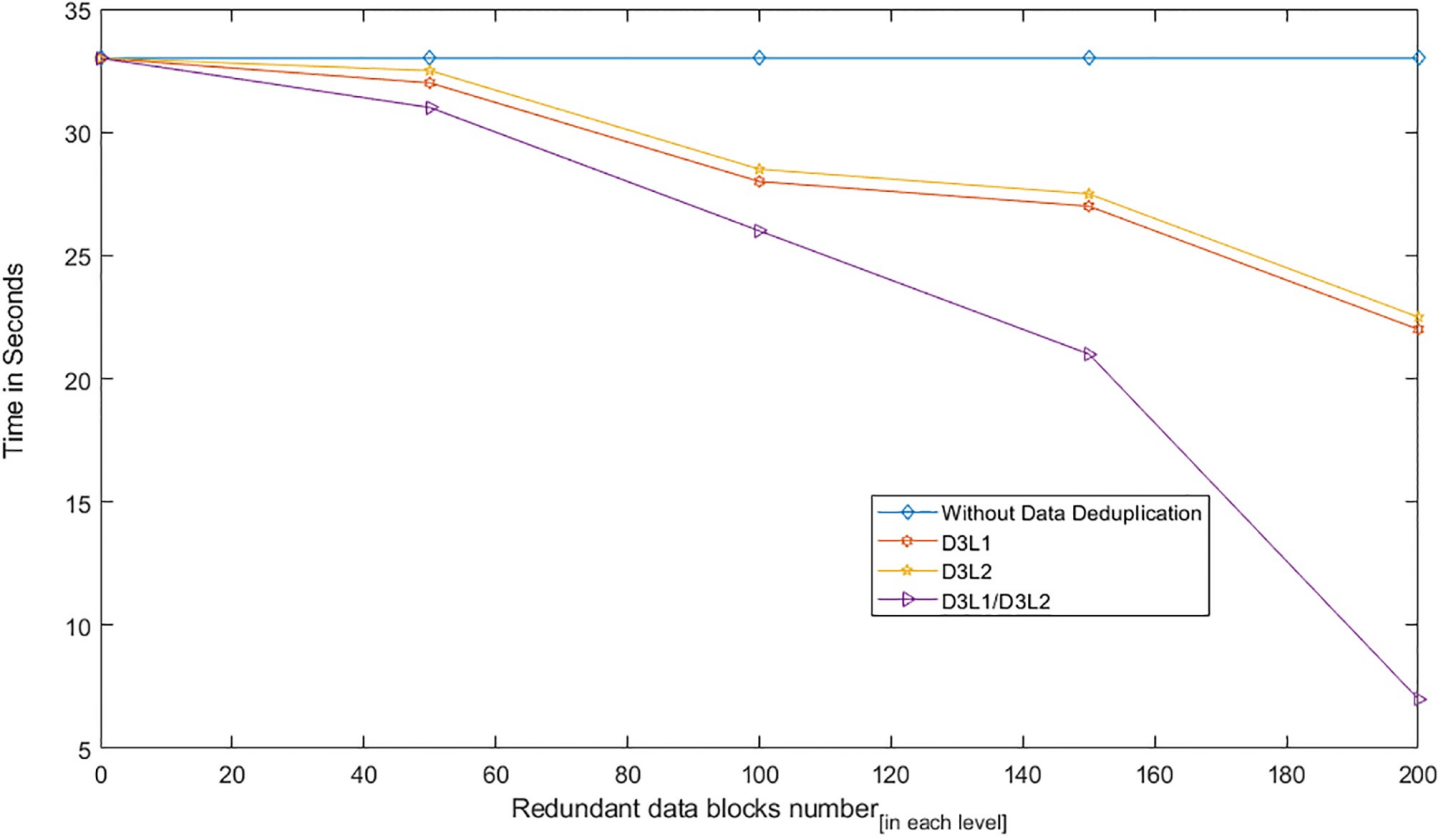
Computational cost of the user in D3U vs the number of redundant data blocks (K = 1000, using Exp2).

Fig 17 compares the computational cost of the user in the D3U against the data block number using Exp1 and Exp2. It shows how the costs increase as the number of data blocks increases. Fig 18 compares the computational cost in D3U with/without the data deduplication approaches. The figure shows that applying two levels in the deduplication approach (D3L1/D3L2) can make the cost more efficient when compared with other approaches. With the increasing redundant data rate, in both levels, the time of execution of D3U for the user decreases as a result.

With DIA-MTTP, there can be multiple PCSes. Each one manages a copy of the user’s data and the associated tags. In the case of using one TPA and a hierarchical approach, the user may need to generate distinctive replicas of the data blocks and their associated tags for making DIA more secure against the collusion attack between the providers. This is where each provider has a distinctive replica, data and tags. Thus, the cost = *n* × (*DFProCost*+ *DFTagGenCost*), where *n* is the number of providers. However, using multiple TPAs in DIA-MTTP, it can help to reduce the cost. As one TPA communicates with one provider, therefore, the user only needs to generate one replica for data blocks and their associated tags. Thus, the cost is constant, i.e. DFProCost + DFTagGenCost, regardless of the number of PCSes.

In D3U, the computational cost that is introduced by the leader provider comes from the L2DataDeduplication protocol execution. The leader provider performs D3L2 to eliminate any identical data blocks among the uploading data blocks. In order to check the duplication, the leader provider first generates a hash value for each of the data blocks and then compares the values with the hash values of the outsourced data. Therefore, the overhead cost comes from the hashing.

**Fig 19:**
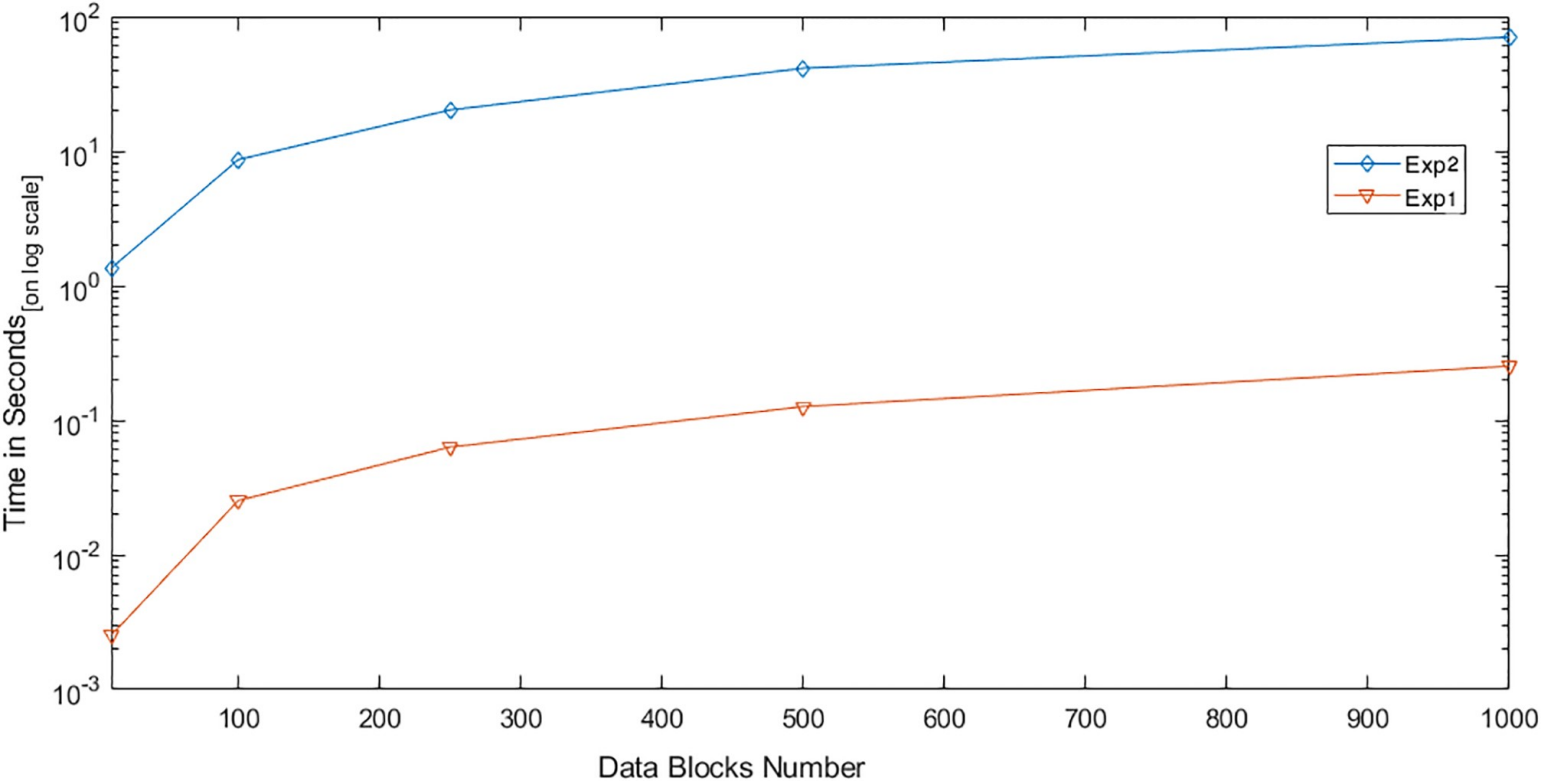
Computational cost of the leader provider in the D3U vs the total number of data blocks in the file.

**Fig 20:**
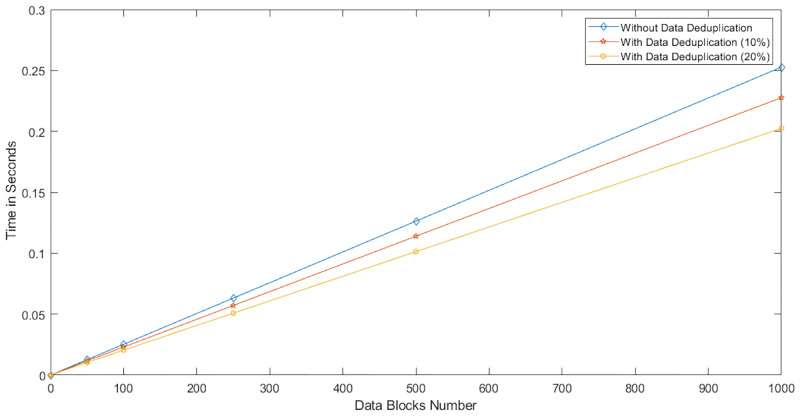
Computational cost of the leader provider in D3U: With/without the data deduplication approach.

Fig 19 compares the computational cost of the leader provider in the D3U against the data block number using two experiments, Exp1 and Exp2. The figure shows that the costs increase as the number of data blocks increases. In the DIA-MTTP, the user applies D3L1 before sending the data blocks to the leader provider. This can help to reduce the cost by (*K* − *d*1). Fig 20 shows how applying data deduplication can help to lessen the computational cost of the leader provider. Under the settings with 500 data blocks and where the data redundancy in D3L1 is 10% and 20%, the cost with deduplication can be reduced by about 15% and 23%, respectively, compared with non-deduplication. The more data redundancy there is under the same or more data blocks or the more data blocks there are under the same the data redundancy, the more there is a reduction in cost.

**Fig 21:**
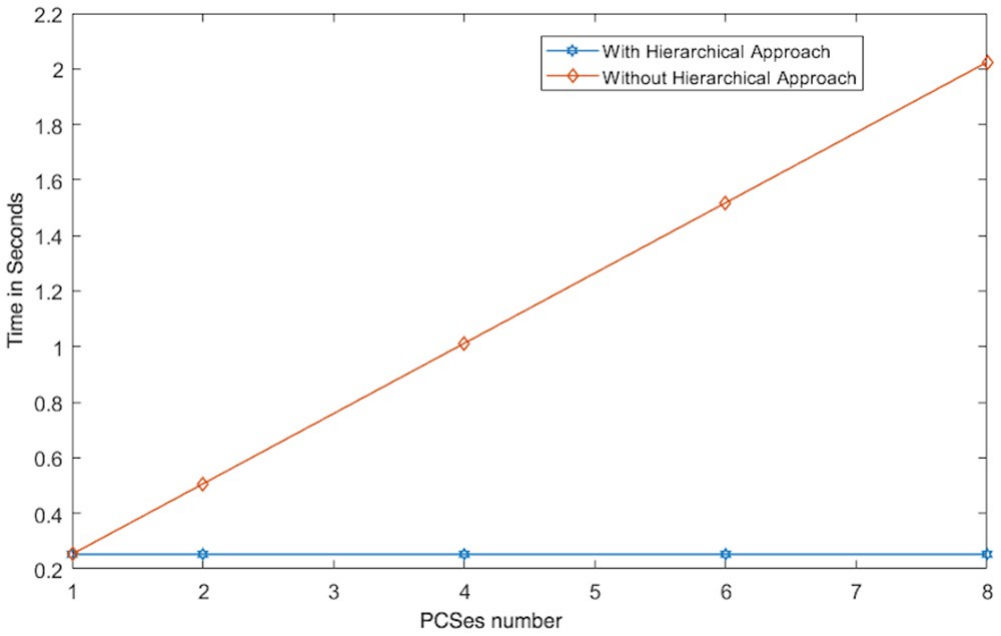
Computational cost of the providers (Leader and non leaders) in D3U: With/without the hierarchical approach. (K = 1000 data blocks).

With regard to the computational cost incurred at other providers (non leaders), each provider in the D3U protocol only receives data and their associated tags from the leader provider and stores in its storage. Fig 21 compares the computational cost of all providers in D3U with and without applying the hierarchical approach using Exp1. The figure shows that use this approach can help in terms of saving the cost incurred by the providers end, where it is constant regardless of the number of PCSes.

In the D3U protocol, only the L-TPA is involved. The L-TPA receives the *En*_*IDTags* from the user and it stores them. Therefore, the L-TPA has not incurred any costly operations.

We measured the communication cost of each entity in the D3U. This was done by accounting how many kilobytes (KB) are sent from this entity.

The user communicates with the leader provider and the L-TPA in the D3U protocol, where three messages are sent, Req.L2DataDeduplication in the L2DataDeduplication protocol, Req.TagsUploading in the TagsUploading protocol, and Req.En_IDTagsUploading in the En_IDTagsUploading protocol. The data blocks of the data file are sent, {*En*_*DB*_*i*_}, in the Req.L2DataDeduplication. The associated tags, *DBTags* and *DBTagTags*, in Req.TagsUploading are sent to the leader provider. The user sends *En*_*IDTags* in Req.En_IDTagsUploading to the L-TPA. The cost is therefore the sum of the following three messages: Req.L2DataDeduplication, Req.TagsUploading, Req.En_IDTagsUploading. The size of all three messages is based on the number and size of the items involved, which in this case is the data blocks and their tags. Each data block is encrypted using the LiSHA algorithm. *DBTag* is computed using the algebraic signature and *DBTagTag* is computed using the BLS signature and *En*_*IDTag* is an encrypted form of *IDTag* using the Paillier scheme.

Using data deduplication as well as the hierarchical approaches can help to save the communication cost of the user. The number of data blocks and their tags can be reduced as the redundant data rate increases using D3L1 and D3L2, respectively. Furthermore, the DIA-MTTP system involves multiple PCSes. The user sends the data blocks and their tags only to the leader provider who is responsible for distributing the copies to each provider.

**Fig 22:**
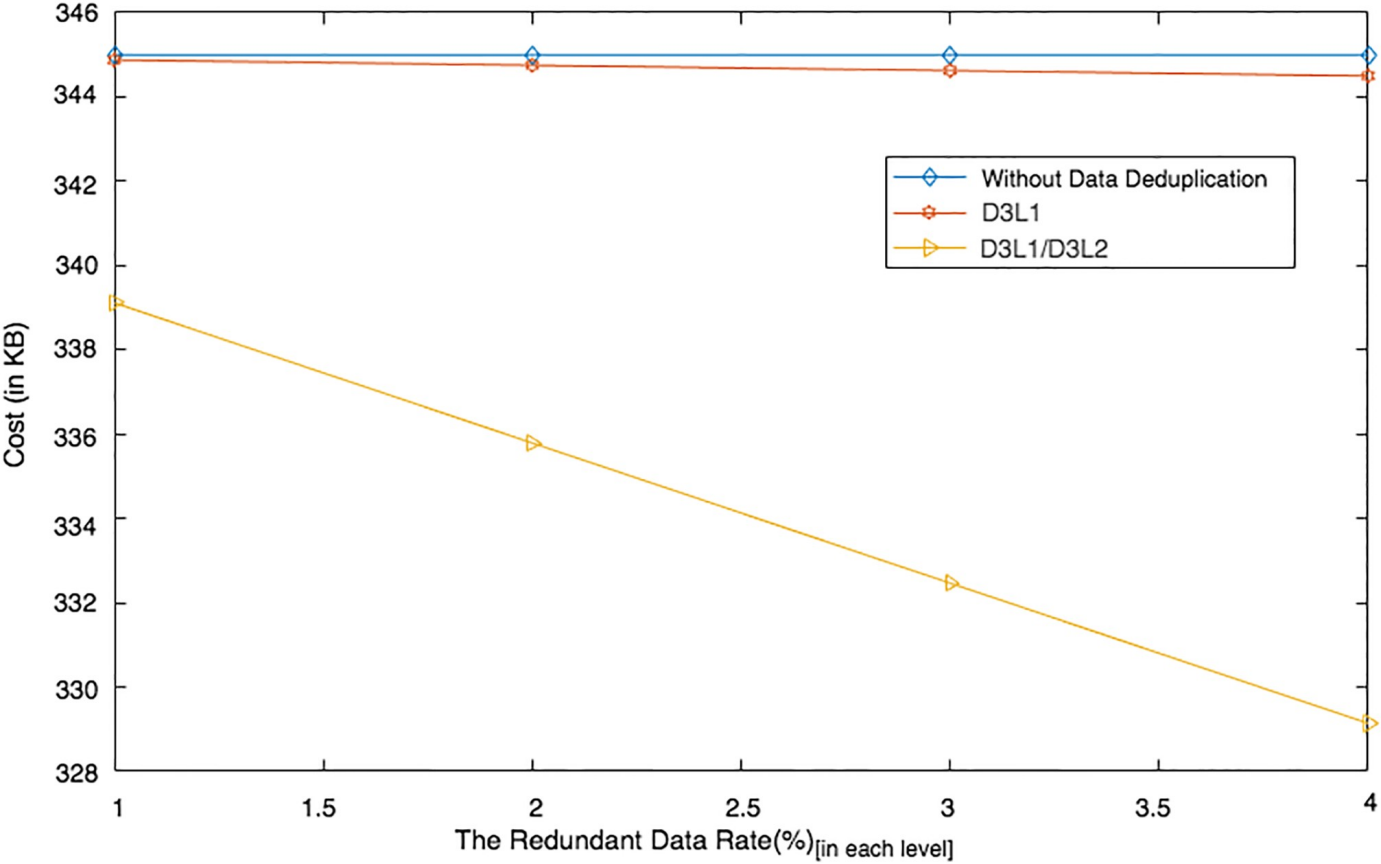
Communication cost of the user in D3U: With/without data deduplication.

**Fig 23:**
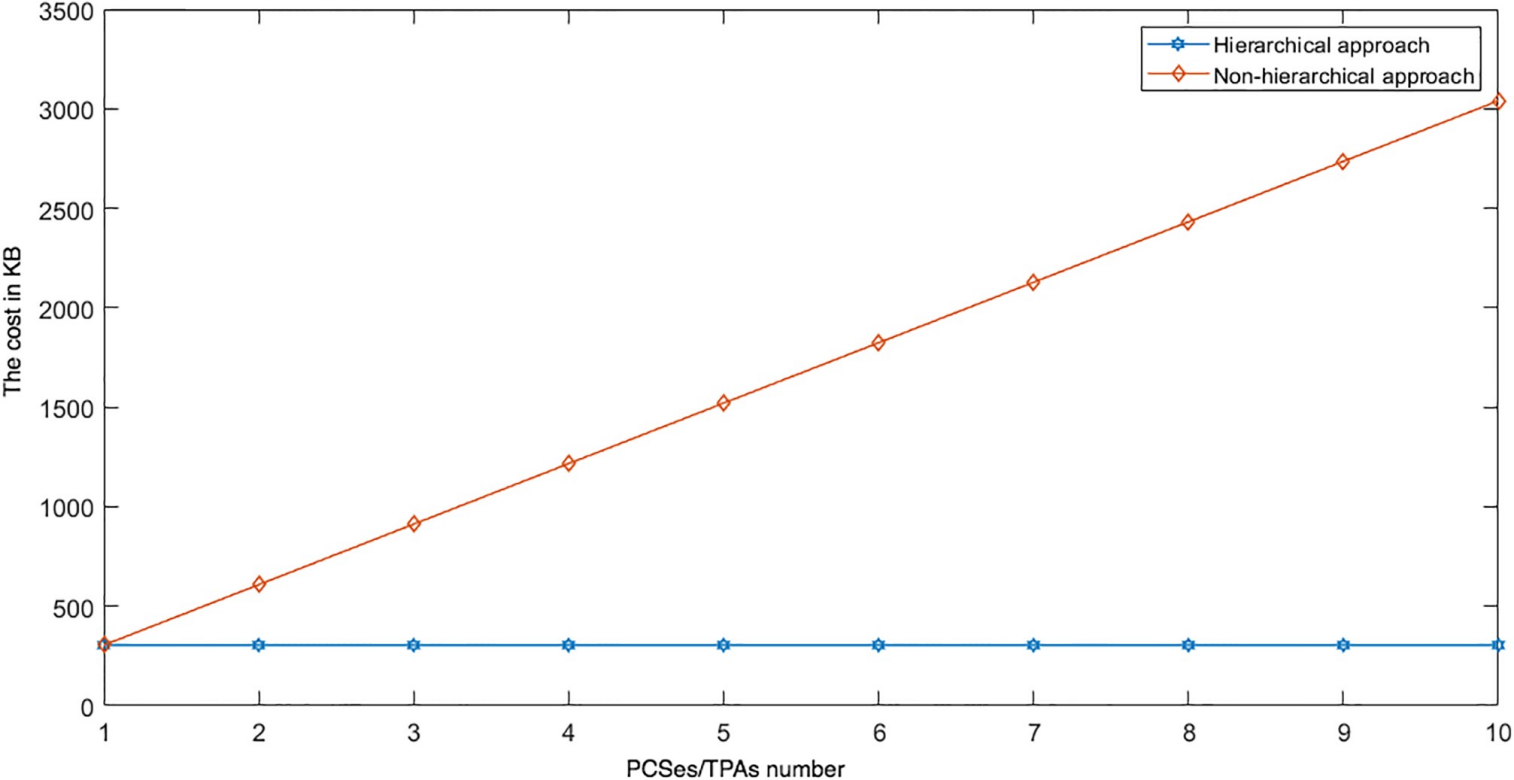
Communication cost of the user in D3U: Hierarchical approach vs non-hierarchical approach. (K = 1000).

Fig 22, by considering the number and bit-length of the items that are involved, compares the communication cost of the user in D3U with and without data deduplication using *p* = 200 bits, *m* = 256 bits, *G*_1_ = 256 bits, and *n*^2^ = 2048 bits. Without using data deduplication, the cost is constant regardless of the redundant data rate. In contrast, with data deduplication, the cost decreases as the redundant data rate increases. Furthermore, the figure shows how the cost can be reduced more when using the two levels of the data deduplication (D3L1 and D3L2) compared to only using D3L1. For example, with a 2% redundant data rate, the cost was reduced by 0.15% using D3L1, while it was reduced by 2.46% using both D3L1 and D3L2. The higher the redundant data rate, the more that the cost can be reduced. Fig 23 compares the communication cost of the user in D3U using the hierarchical and non-hierarchical approaches. The figure shows that the cost increases linearly according to the number of PCSes/TPAs involved when using the non-hierarchical approach. On the other hand, when using the hierarchical approach, the cost is constant regardless of the number of PCSes and TPAs.

In the D3U protocol, the leader provider receives the data blocks and their associated tags from the user in Req.L2DataDeduplication and Req.TagsUploading, receptively. Upon receiving Req.L2DataDeduplication, the leader provider first responds to the user by sending Res.L2DataDeduplication. Then, it can send Req.DataTagsUploading to each non leader provider when it has the tags. Finally, it sends Res.TagsUploading to confirm that the data blocks and their associated tags are distributed and stored in all PCSes correctly. Res.L2DataDeduplication includes the duplication result. As shown in algorithm 2, in the case where the data block is duplicated, the non leader provider sends the ID of a data block in M2T that is identical to the received data block. Otherwise, it sends the empty value to indicate that the data block is non-duplicated. In the PCS environment, the number of data blocks can be huge. We can use 64 bits or more to represent the IDs of the data blocks in M2T for each user. The size of Res.L2DataDeduplication is based on the data redundancy rate. Res.TagsUploading includes the acknowledgement that is used to indicate that the data are stored in the PCS correctly. It can be represented using one bit, i.e. 1. Regarding the Req.DataTagsUploading message, it includes three sets of *En*_*DBs*, *DBTags* and *DBTagTags* or the IDs of the data blocks in the case where they are duplicated. Upon receiving DataTagsUploading, each non leader provider stores the data blocks, {*En*_*DB*_*i*_}, and their associated tags, {*DBTag*_*i*_} and {*DBTagTag*_*i*_} in its M2Ts. The provider then sends Res.DataTagsUploading as a response. Res.DataTagsUploading is similar to the Res.TagsUploading sent by the leader provider. Therefore, the non leader provider has incurred a constant communication cost regardless of the number of data blocks and their tags.

**Fig 24:**
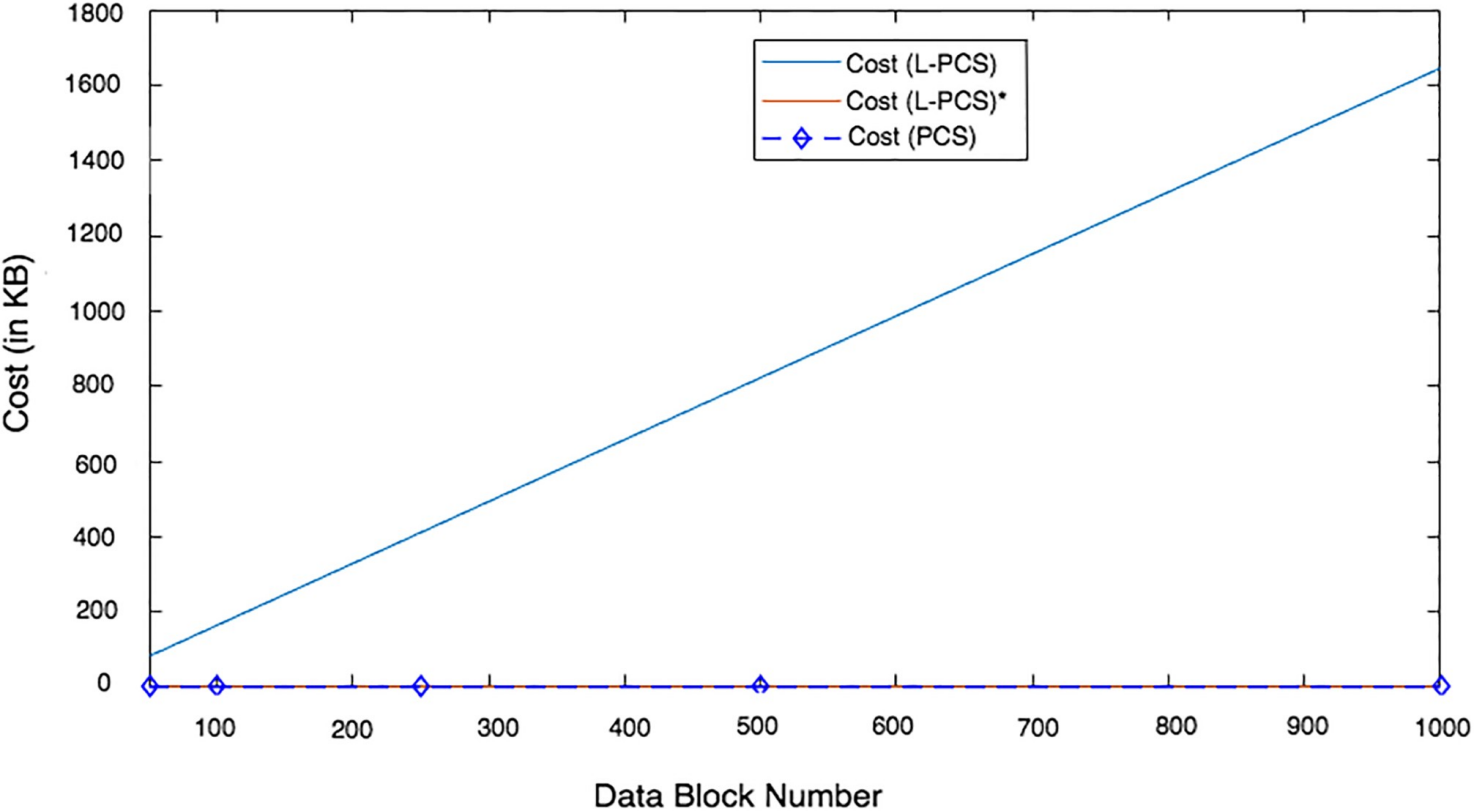
Communication costs for the providers vs the number of data blocks: With/without a hierarchical approach. (n = 6 PCSes, * is Non-Hierarchical approach).

**Fig 25:**
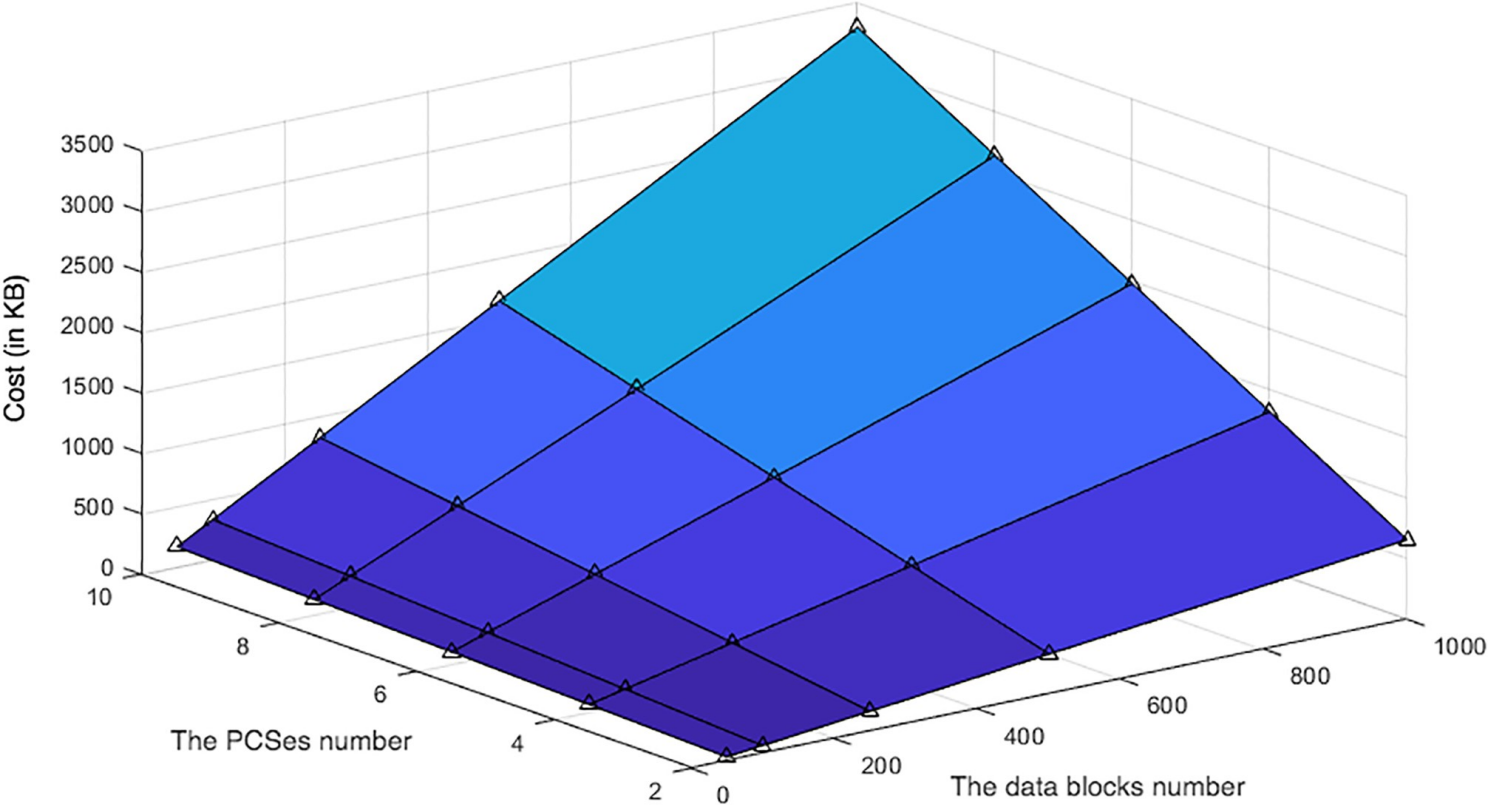
Communication cost incurred by the leader provider regarding the number of data blocks and PCSes.

Fig 24 compares the communication cost incurred by the leader provider and the individual non leader provider in the D3U using two approaches: Hierarchical and Non-Hierarchical (without data deduplication). The figure shows that the cost increases as the data block number increases using the hierarchical approach. It is a constant using the Non-Hierarchical approach and it is similar to the non leader provider. Fig 25 shows that the communication cost incurred by the leader provider increases as the number of data blocks and the number of PCSes increase. However, this lessens the communication cost for the user.

In the D3U protocol, only the L-TPA communicates with the user. The L-TPA receives the set of *En*_*IDTags*, {*En*_*IDTag*_*i*_} in Req.En_IDTagsUploading. Then, it responds by sending the Res.En_IDTagsUploading message. The Res.En_IDTagsUploading includes an acknowledgement that the {*En*_*IDTag*_*i*_} are stored in the L-TPA correctly. As mentioned above, the acknowledgement can be represented using one bit, 1. This means that L-TPA incurred a negligible and constant communication cost in the D3U protocol regardless of the number of the uploaded data blocks.

### Introduced cost by LoA1DV

In the following section, the computational and communication costs for each entity in LoA1DV have been presented in detail.

In LoA1DV, as mentioned in LoA1DV Section, only public verification is performed. This means that the user is not involved and so s/he has not incurred any cost in LoA1DV. The user delegates the L-TPA and the non leader TPAs to perform the verification and send the verification result to him/her.

**Fig 26:**
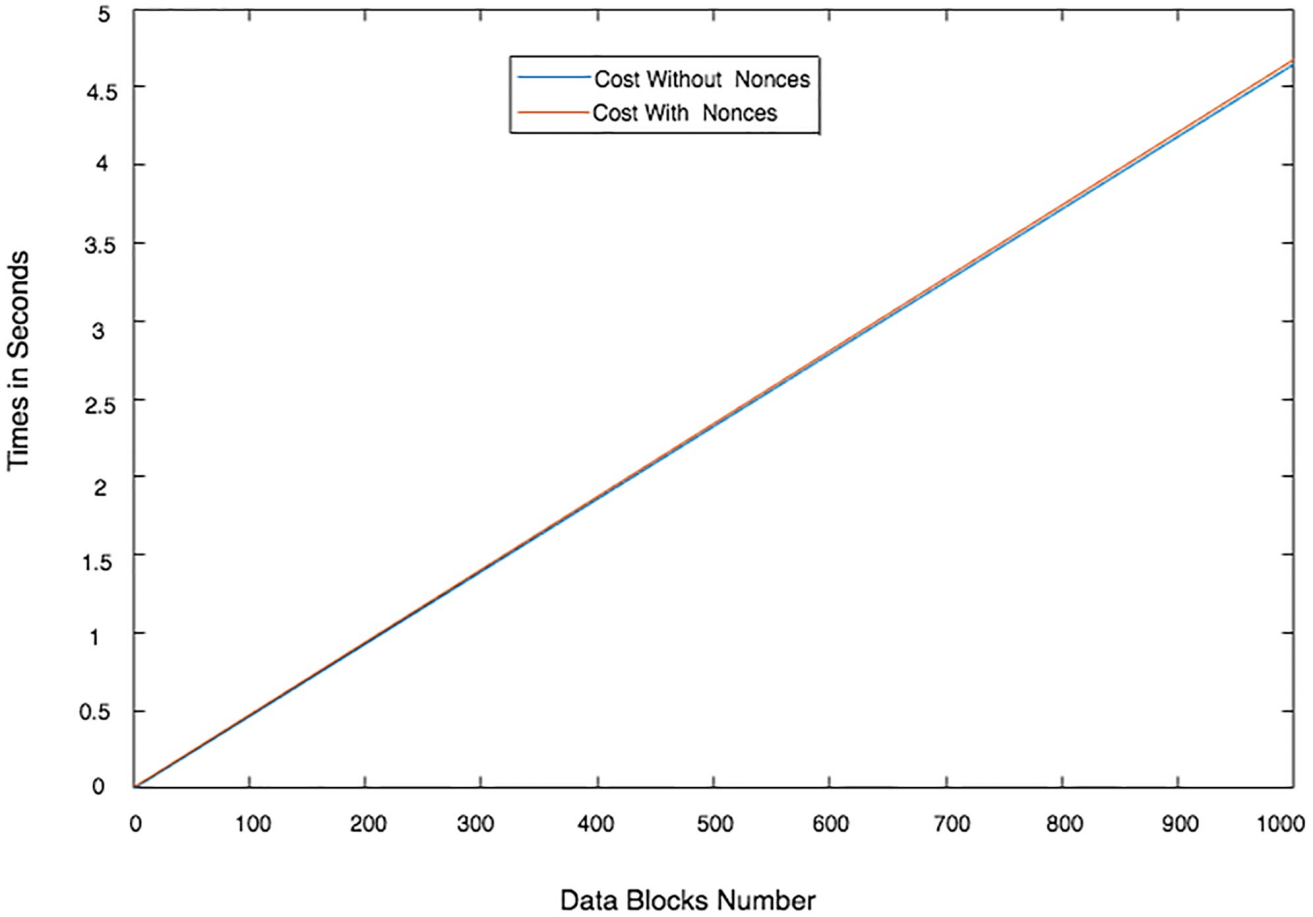
Computational cost of PCS in LoA1DV: With/without nonces.

The leader provider, like the user, as mentioned in LoA1DV Section, is not involved in the LoA1DV protocol and so it has not incurred any cost. Thus, the non leader providers are involved in LoA1DV. Each provider computes public proofs to send them to the associated TPA. Therefore, the computational cost of the provider is the cost of the public proofs generation, *PubProofGenCost*. As mentioned in the PubProofsGen algorithm, three types of the public proofs, *PubDBProof*, *PubDBTagProof* and *PubDBTagTagProof*, are calculated based on the respective items, {*En*_*DB*_*i*_}, {*DBTag*_*i*_} and {*DBTagTag*_*i*_}, where, 0 ≤ *i* < *C*. Furthermore, for replay attack prevention, the provider should use fresh nonces that have been sent by the TPA in the proofs generation. Fig 26 compares the computational cost of PCS in LoA1DV with and without nonces. The figure shows that the costs increase as the number of data blocks increases. Furthermore, an additional cost is introduced when using nonces is negligible.

The public verification in LoA1DV is distributed between the L-TPA and the non leader TPAs. The cost incurred by the L-TPA can be measured using three metrics: PubChallGenCost (it is a cost incurred in generating a public challenge), DBProofTagGenCost (it is a cost incurred in generating a tag for *PubDBProof*) and DBTagTagProofVerCost (it is a cost incurred in verifying *PubDBTagTagProof*). The computational cost of the L-TPA in LoA1DV is PubChallGenCost + DBProofTagGenCost + DBTagTagProofVerCost.

As shown in the PubChalGen algorithm, the L-TPA computes a tag for the aggregated value of {*ProofNonce*_*i*_} as well as the tags for {*PCSNonce*_*j*_} using the AS. The DBProofTagGenCost, as seen in the DBProofTagGen algorithm, is the cost of computing a tag for *PubDBProof* using the algebraic signature. The L-TPA receives the *PubDBProofs* from all TPAs, but it generates a tag for only one copy of *PubDBProof*. DBProofTagGenCost is a constant regardless of the number of receiving *PubDBProofs*, i.e. O(1). Furthermore, the L-TPA should compute an aggregated *En*_*IDTag* value for the set {*En*_*IDTag*_*i*_}. {*En*_*IDTag*_*i*_} are associated with {*DBTag*_*i*_} that are used for computing the *PubDBTagProof*.

As shown in the DBTagTagProofVer algorithm, the *PubDBTagTagProof* verification can be performed in a batch manner. This in order to optimise the cost. This is where the L-TPA can verify all *PubDBTagTagProofs* that are received from all of the TPAs in one operation. The cost is based on the number of pairing operations ($Pair_{G_{1},G_{2}}$). This is which is the most costly operation. In batch verification, the number of the pairing operations is constant regardless of the number of received *DBTagTagProofs* (the total number of TPAs), $2\times Pair_{G_{1},G_{2}}$. In the individual verification, the number of pairing operations increases with the number of TPAs, $2\times n\times Pair_{G_{1},G_{2}}$. By increasing the number of TPAs, the number of $Pair_{G_{1},G_{2}}$ increases and the computational cost of the *PubDBTagTagProof* verification increases, accordingly.

For the non leader TPAs, the incurred cost for each TPA can be measured using two metrics, DBTagProofVerCost (a cost incurred when verifying *PubDBTagProof*) and DBTagMappingCost (a cost incurred when generating a map value for *PubDBTagProof*). The computational cost is incurred on part of the TPA in the LoA1DV is *DBTagVerCost*+ *DBTagMappingCost*. *PubDBTagProof* is a set that consists of *C* items according to Eq(12) where each item is a proof of one *DBTag*. The TPA computes a map value for each item of *PubDBTagProof*. The cost is based on the number of *PubDBTagProof*, i.e. C.

Using the collaborative verification approach can help lessen the cost of the verification protocol. As mentioned above, the L-TPA verifies *PubDBTagTagProofs*, while the other TPAs verify their associated *PubDBTagProofs*. By applying this approach, the total cost of the verification protocol (LoA1DV) incurred at the TPAs including L-TPA is (*n* − 1) × (*PubChallGenCost* + *DBTagProofVerCost* + *DBTagProofMapCost*) + *DBProofTagGenCost* + *DBTagTagProofVerCost*, compared to the cost without the aforementioned approach, i.e., it is *n* × (*PubChallGenCost* + *DBProofTagGenCost* + *DBTagProofVerCost*+ *DBTagProofMapCost* + *DBTagTagProofVerCost*).

The user sends Req.PublicDataVerification, in the LoA1DV protocol, to the L-TPA to instruct it to perform the public verification. This message includes the signature of the *FileTag* which is associated with the file that the user wants to verify its integrity. This is in addition to whichever verification level is required, i.e. LoA1 or LoA2. Thus, the communication cost that is incurred by the user is |*FileTagSig*| + |*LoA*|. The *FileTagSig* can be computed using any public key encryption, e.g. RSA. One bit can be used to represent the verification level; 0 for LoA1 and 1 for LoA2. For example, when using the 1024-RSA algorithm, the cost is 1024 +1 = 1025 bits ≈ 0.13 KB. Thus, the total communication cost for the user in LoA1DV is a negligible constant cost.

In LoA1DV, as mentioned above, only the non leader providers are involved. Each provider sends Res.PublicProofsFromPCS to its associated TPA. This message includes *PubDBProof*, *PubDBTagProof*, and *PubDBTagTagProof*. *PubDBProof* and *PubDBTagTagProof* are aggregated values of C of *En_DBs* and *DBTagTags*, respectively. *PubDBTagProof* consists of C items, {*PubDBProof*_*i*_}, as in Eq(12). Thus, the communication cost of the provider is |*En*_*DB*|+ (*C* × |*DBTag*|) + |*DBTagTag*|. This cost is based on the total number and the size of {*DBTag*_*i*_}. This means that the cost increases as the number of *DBTag*_*i*_ and its size increase.

**Fig 27:**
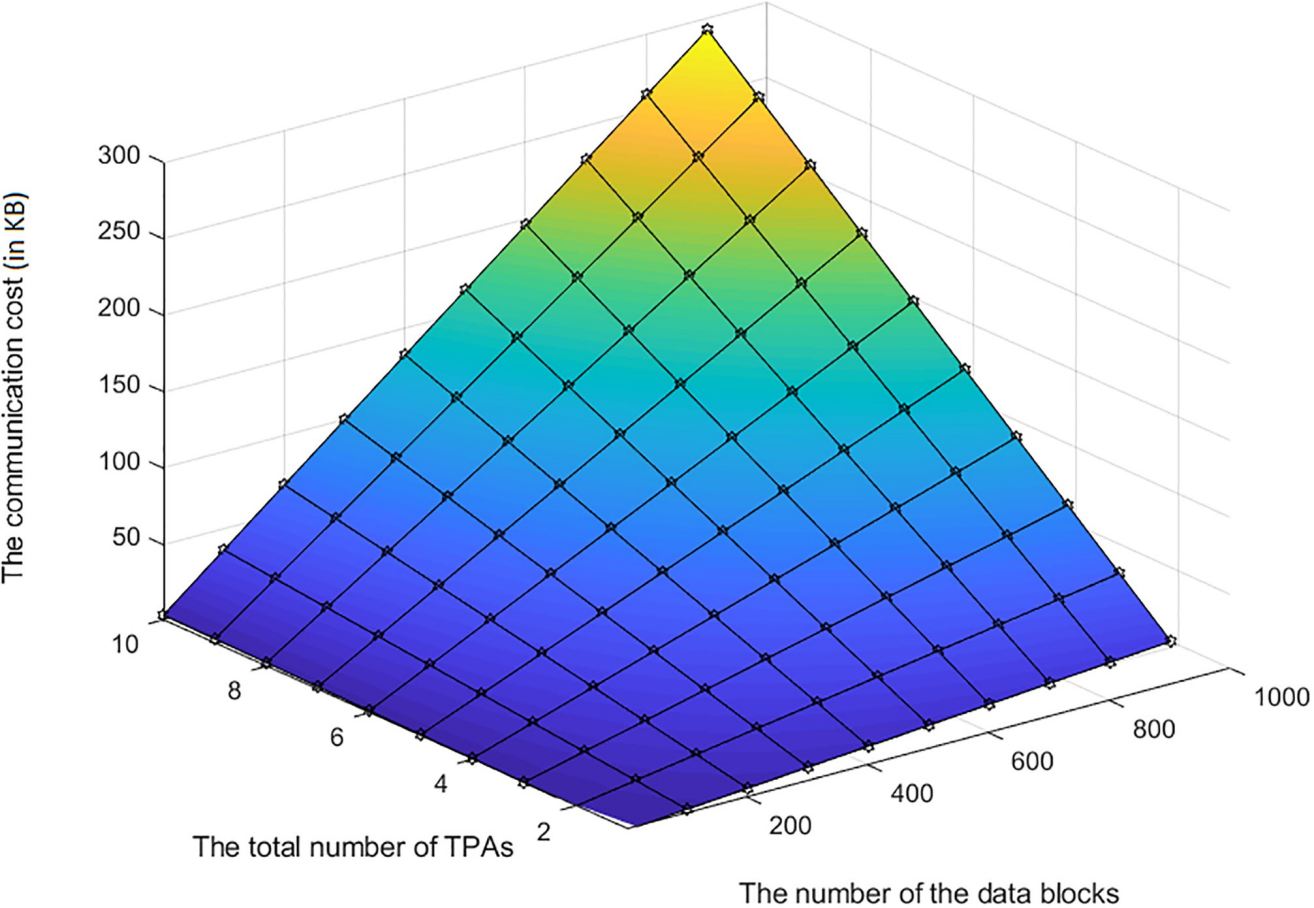
Communication cost of the L-TPA in LoA1DV vs the TPAs and the number of data blocks. (|*En*_*DB*| = 0.025 KB and |*DBTag*| = 0.032 KB).

In LoA1DV, the L-TPA sends the following three messages, Req.PublicProofsFromTPA, Req.DBTagProofVerification, and Res.PublicDataVerification to the non leader TPAs and to the user, respectively. The Req.PublicProofsFromTPA, which is sent to each non leader TPA, includes two sets. The first set is made up of the indexes of the chosen data blocks (their positions). The second set is their associated *ProofNonces*. In addition to these sets, there is *AggProofNonceTag*, *PCSNonce*_*j*_ and *PCSNonceTag*_*j*_. The Req.DBTagProofVerification message includes two values *DBProofTag* and *AggEn_IDTag*. The *DBProofTag* is computed using the algebraic signature, and the *AggEn_IDTag* is aggregated value using C of *En*_*IDTags*. Res.PublicDataVerification in the LoA1DV protocol includes *DBTagTagProofVerResult* (i.e. the public data verification result, 0/1). The communication cost of the L-TPA in the LoA1DV protocol, CommLTPALoA1DVCost, is (*n* − 1) × (Req.PublicProofsFromTPA + Req.DBTagProofVerification) + Res.PublicDataVerification, i.e. it is as follows (*n* − 1) × (*C* × (|*I*_*i*_|+ |*ProofNonce*_*i*_|) + |*AggProofNonceTag*| + |*PCSProof*_*j*_| + |*PCSProofTag*_*j*_| + |*DBProofTag*|+ |*AggEn*_*IDTag*|) + |*DBTagTagProofVerResult*|. Fig 27 compares the communication cost of the L-TPA against the number of TPAs and the data blocks. The cost increases significantly as the number of the TPAs and the chosen data blocks increases. However, this is in order to save the bandwidth cost of the user.

**Fig 28:**
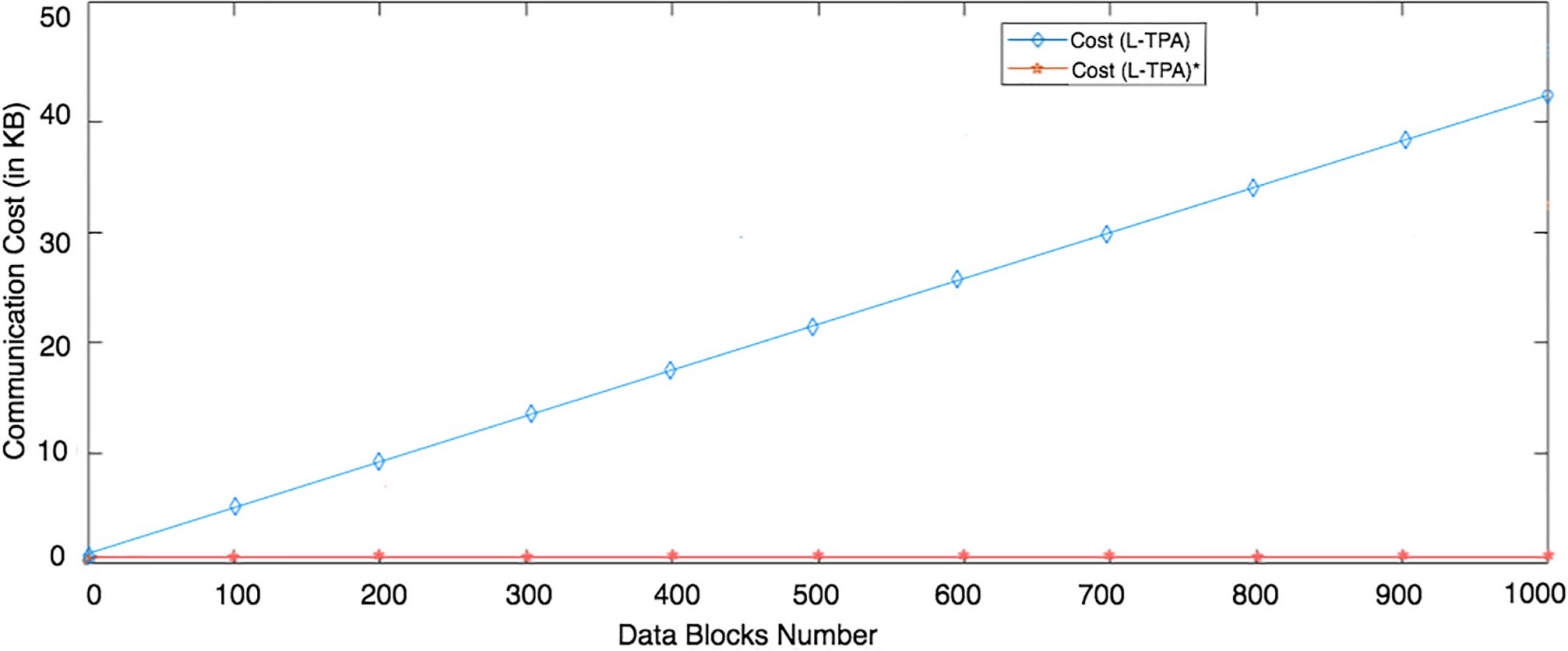
Communication cost of the L-TPA in LoA1DV against the number of data blocks. (* is Key-based approach, n = 2).

To lessen the communication cost of the L-TPA, the L-TPA can send a key to the TPA, which can be used to generate the values of the data block indexes set and their associated *ProofNonces*, which is an alternative to sending the two sets separately. Fig 28 compares the communication cost of the L-TPA in LoA1DV against the number of challenged blocks using two approaches (NonKey-based and Key-based). From the result in the figure, it can be seen that the cost using the NonKey-based approach increases as the data block number increases. The cost using the Key-based approach is constant regardless of the number of data blocks. Furthermore, It incurs less of a cost compared to the NonKey-based approach.

Regarding the communication cost of a non leader TPA in the LoA1DV protocol, the cost comes from sending *Req*.*PublicProofsFromPCS*, *Res*.*PublicProofsFromTPA* and *Res*.*DBTagProofVerification*. The first message, *Req*.*PublicProofsFromPCS*, is similar to *Req*.*PublicProofsFromTPA*. The second message, *Res*.*PublicProofsFromTPA*, includes *PubDBProof* and *PubDBTagTagProof*. These are the aggregated values, as mentioned above. The Res.DBTagProofVerification includes *PubDBTagProofVerResult* and *DBTagProofMapValue*. The *PubDBTagProofVerResult* can be 1 or 0 bit. The *DBTagProofMapValue*, as shown in Eq(19), is an aggregated value of {*DBTagProofMapValue*_*ji*_}. Thus, the cost is the sum of these messages i.e. *Req*.*PublicProofsFromPCS* + *Res*.*PublicProofsFromTPA* + *Res*.*DBTagProofVerification*, it is as follows (C × (|*I*_*i*_| + |*ProofNonce*_*i*_|) + |*PCSNonce*_*j*_| + |*PCSNonceTag*_*j*_| + |*PubDBTagProofVerResult*| + |*DBTagProofMapValue*| + |*PubDBProof*| + |*PubDBTagTagProof*|. Using the Key-based approach, the cost can be constant regardless of the number of data blocks, i.e. |*Key*| + |*PCSNonce*_*j*_| + |*PCSNonceTag*_*j*_| + |*PubDBTagProofVerResult*| + |*DBTagProofMapValue*| + |*PubDBProof*| + |*PubDBTagTagProof*|.

### Introduced cost by LoA2DV

In the following section, the computational and communication costs for each entity in LoA2DV have been presented in detail.

In the LoA2DV protocol, the user is involved. The total cost incurred by the user in LoA2DV is PriChallGenCost + FPriProofVerCost. The PriChallGenCost is incurred as a part of generating a private challenge, and the FPriProofVerCost is incurred as a part of performing a private verification. The private challenge, as shown in the PriChalGen algorithm, depends on the public verification that has been received from L-TPA. The user, in addition to using the public challenge items, chooses a random number for the leader provider and then generates its tag using the AS. As shown in the FPriProofsVer algorithm, the user computes a fresh *FPriDBTagProof* using *FPriDBProof* and aggregated value of the *IDTags* that is associated with the data blocks in the proof (FPriDBProof) in order to compare it with the received *FPriDBTagProof*.

Using the hierarchical approach can help to reduce the cost for the user, where it is not including $Add_{Z_{p}}$. It is a more costly operation when compared with *Add*_*AS*_ and *AS*-*G* as shown in Table 5. The cost in the second approach, i.e. without the hierarchical approach, increases as the number of $Add_{Z_{p}}$ increases by increasing the number of PCSes. Without the hierarchical approach, the user should first retrieve all of the proofs from all providers. He/she can then verify them either collectively or individually.

**Fig 29:**
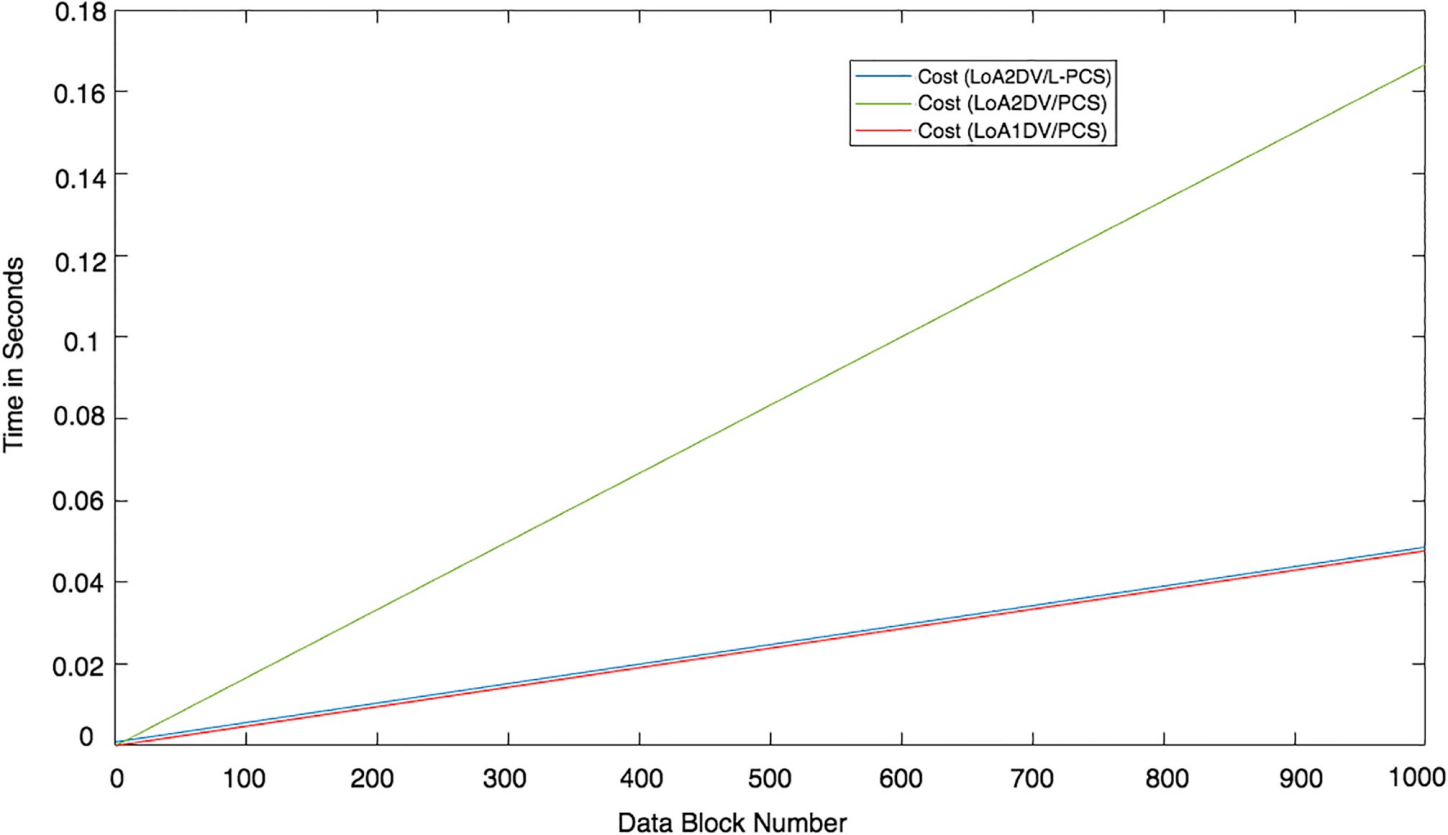
Computational costs for the leader provider and the non leader provider in LoA1DV and LoA2DV vs the number of data blocks. (n = 20).

In LoA2DV, the leader provider is involved in the data verification, unlike in LoA1DV. Thus, the leader provider generates its proofs and then computes the final proofs using its own proofs and the proofs of other providers. Regarding the non leader providers, each provider in the LoA2DV in addition to the public proofs generation, should generate private proofs to send to the leader provider. Fig 29 compares the computational cost of the leader provider and the computational costs incurred by the non leader provider in both LoA1DV and LoA2DV. These costs increase as the number of data blocks increases. Based on the results shown in the figure, we can see that the cost introduced by the non leader provider in LoA1DV is less than the cost introduced in the LoA2DV. Additionally, the cost of generating the final proofs incurred by the leader provider is not a high cost. It is nearly equal to the cost of the non leader provider in LoA1DV.

In the LoA2DV protocol, the public and private verifications are performed where the costs incurred by the L-TPA and non leader TPAs are identical to the cost in the LoA1DV protocol. They have incurred the same cost regardless of which verification protocol is executed, i.e., LoA1DV or LoA2DV.

**Fig 30:**
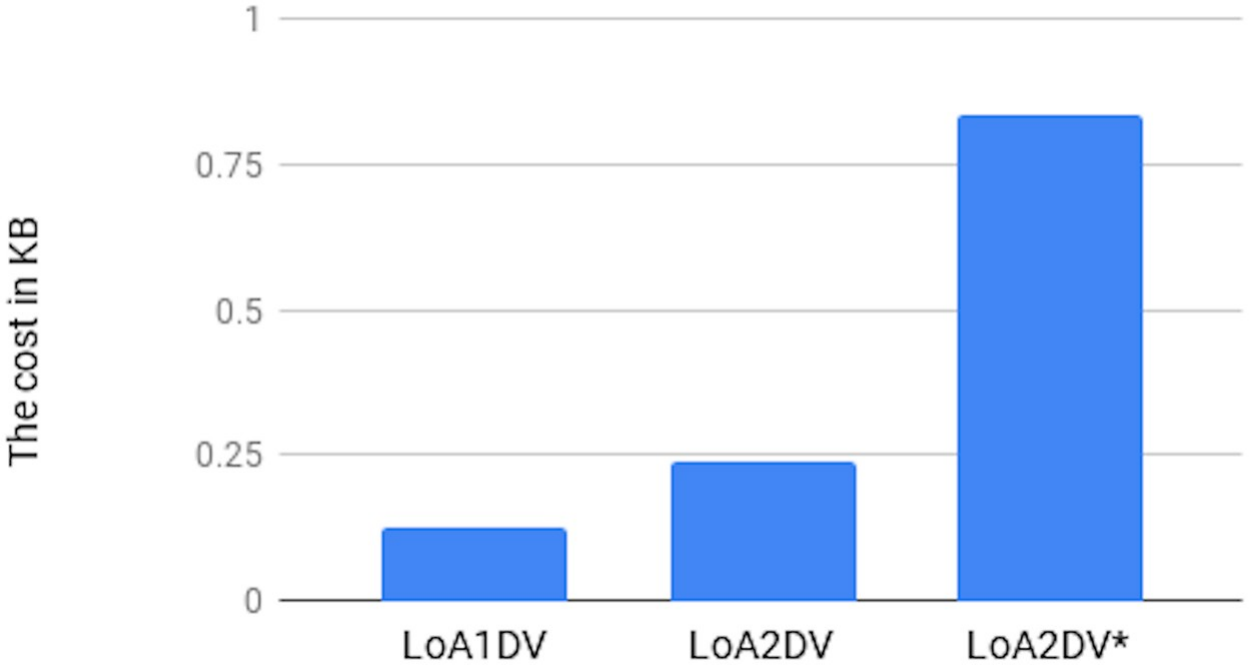
Communication cost for the user in LoA1DV and LoA2DV. (* the NonKey-based approach, and C = 20 data blocks).

The user in LoA2DV, as mentioned above, sends a delegation message to L-TPA as in the LoA1DV. In addition to this message (Req.PublicDataVerification), the user communicates with the leader provider by sending Req.PrivateProofsFromLPCS, the private challenge message, in order to receive the private proofs from all providers. The private challenge message consists of a set of data block identifiers and their associated nonces, *ProofNonces* and a tag of *LPCSNonce* (*LPCSNonceTag*). Fig 30 compares the communication cost for the user in LoA1DV and LoA2DV. From the results shown in the figure, it can be seen that the user incurs a cost in LoA1DV that is about 16% of the cost in LoA2DV using the NonKey-based approach. However, when using the Key-based approach, the cost of LoA2DV on the user is about double its cost in LoA1DV. This means that when using this approach in LoA2DV, it releases the user from being burdened with a high communication cost. When using the hierarchical and Key-based approaches, this cost is constant regardless of the number of PCSes.

In the LoA2DV protocol, before the leader provider responds to the user’s request (Req.PrivateProofsFromLPCS), it first communicates with each provider to get its private proofs by sending Req.PrivateProofsFromPCS. Then it sends Res.PrivateProofsFromLPCS. This message includes the items of the private challenge, except the *LPCSNonceTag*. The Res.PrivateProofsFromLPCS includes the final private proofs. As mentioned in Algorithm 13, the final private proofs consist of *PriDBProof* and *PriDBTagProof*. The private proofs do not include *DBTagTagProof* as the public proofs do. Furthermore, they are also a constant size because they are all aggregated values. Thus the private proofs can be of a smaller size compared with the public proofs.

**Fig 31:**
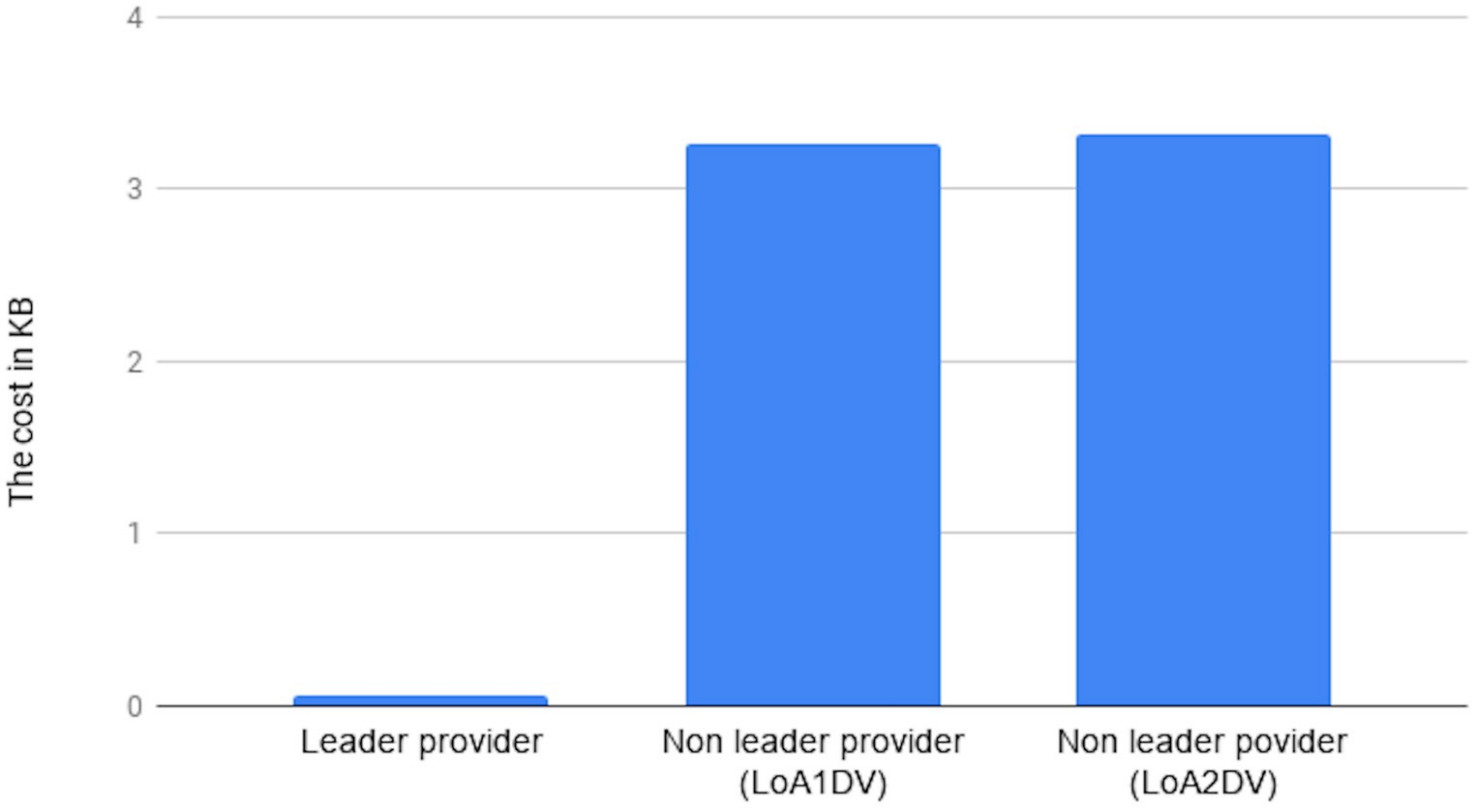
Communication cost for the leader provider and the non leader provider in LoA1DV and LoA2DV. (C = 100, n = 2, |*m*| = |*G*_1_| = 0.032 KB, considering only the proofs cost).

**Fig 32:**
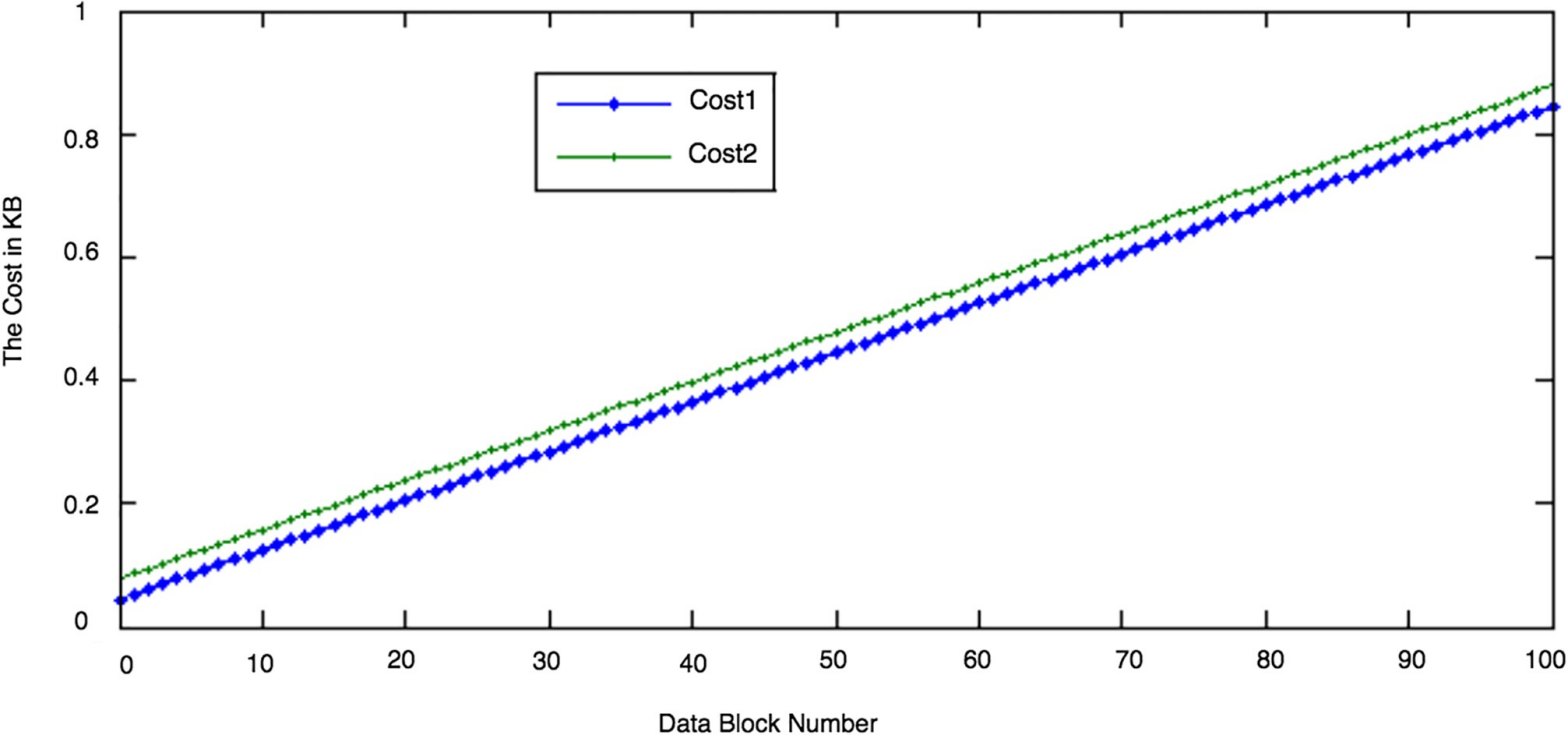
Communication cost of the non leader provider in LoA1DV and LoA2DV vs the data block number.

Fig 31 compares the communication costs of the leader provider and of a non leader provider in both LoA1DV and LoA2DV. From the results in the figure, we can see that the leader provider has incurred a lesser cost compared to the non leader provider. Fig 32 compares the communication cost for the non leader provider in LoA1DV and LoA2DV against the number of data blocks. Both costs increase as the data block number increases. Additionally, the LoA2DV does not incur a high cost on part of the non leader provider, as the cost is nearly equal to the cost in the LoA1DV.

In the LoA2DV protocol, in addition to the verification result that sent in the message (Res.PublicDataVerification), the L-TPA shares the challenge items, i.e. the indexes of the challenging data blocks and their associated *ProofNonces* or use a key to use in generating these values, and the aggregated value, *AggPCSNonceTag*.

**Fig 33:**
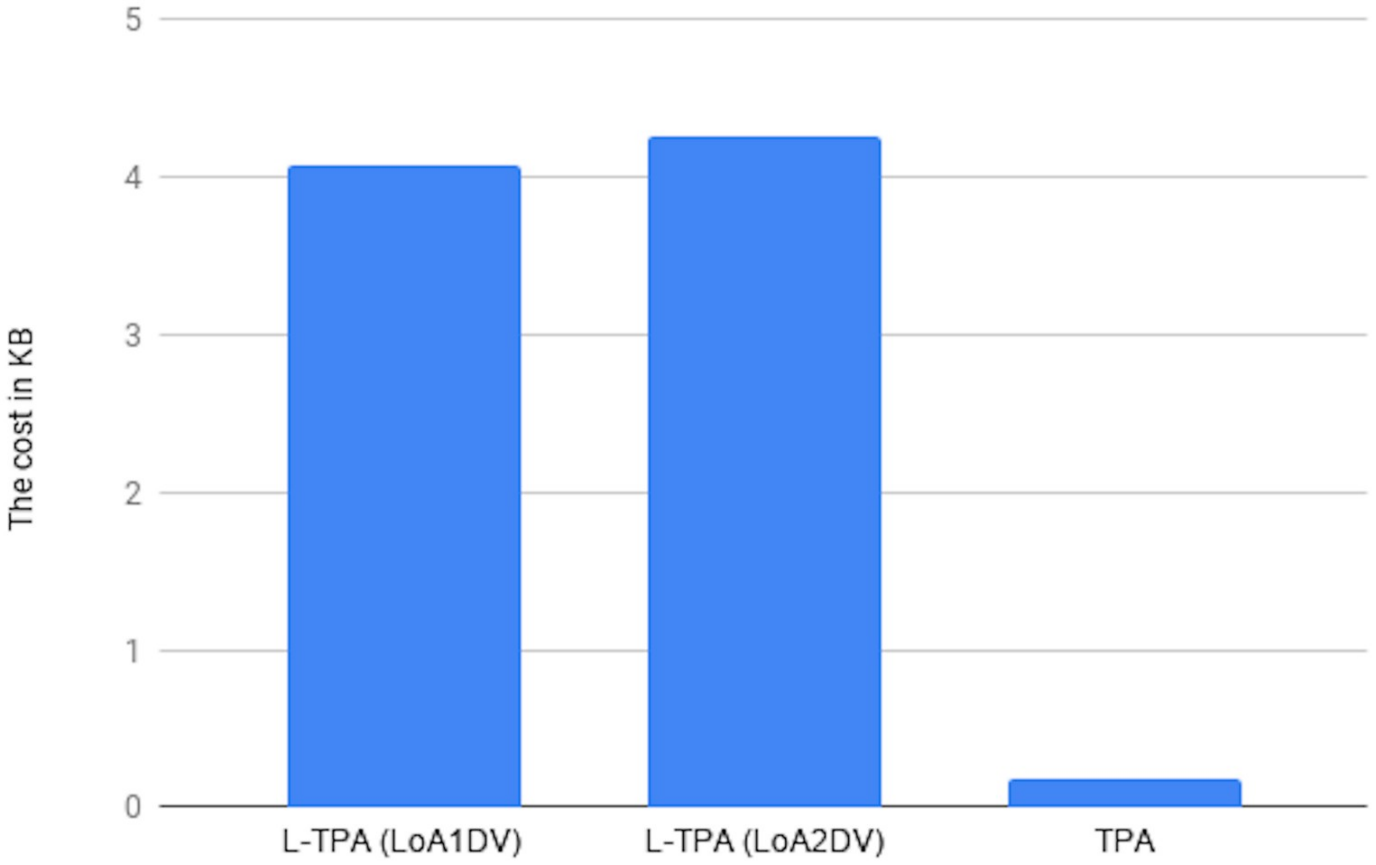
Communication costs of the L-TPA and the non leader TPA in LoA1DV and LoA2DV. (n = 10, |*m*| = |*G*_1_| = 256 bits, |*p*| = 200 bits, using Key-based approach).

Regarding the communication cost of the other TPAs (non leaders), they incurred a cost that is equal to the cost in the LoA1DV. In other words, the LoA1DV and LoA2DV protocols have introduced the same cost as the TPAs in the DIA-MTTP. Table 7 summarises the complexities of the communication costs for the L-TPA and the non leader TPA. Fig 33 compares communication costs of the L-TPA and the TPA in both LoA1DV and LoA2DV. The communication cost incurred in the L-TPA in LoA2DV is a little more compared to the cost in the LoA1DV. Furthermore, the communication cost of the TPA a is less than the communication cost of the L-TPA a in both LoA1DV and LoA2DV.

**Table 7:** Computational cost of tag generation against different sizes of data file and data block (in seconds).

Block Size	2 KB	4 KB	8 KB	16 KB	32 KB	64 KB	128 KB	256 KB	512 KB	1024 KB
**Block numbers (**1 × 10^6^ **KB)**	5.2 × 10^5^	2.6 × 10^5^	1.3 × 10^5^	6.6 × 10^4^	3.3 × 10^4^	1.6 × 10^4^	3.2 × 10^3^	4.1 × 10^3^	2.1 × 10^3^	1 × 10^3^
**Time of** 1 × 10^6^ **KB**	2.8 × 10^4^	1.4 × 10^4^	7 × 10^3^	3.5 × 10^3^	1.7 × 10^3^	8.7 × 10^2^	4.4 × 10^2^	2.2 × 10^2^	1.1 × 10^2^	5.4 × 10
**Block numbers (**1 × 10^7^ **KB)**	5.2 × 10^6^	2.6 × 10^6^	1.3 × 10^6^	6.6 × 10^5^	3.3 × 10^5^	1.6 × 10^5^	8.2 × 10^4^	4.1 × 10^4^	2.1 × 10^4^	1 × 10^4^
**Time of** 1 × 10^7^ **KB**	2.8 × 10^5^	1.4 × 10^5^	7 × 10^4^	3.5 × 10^4^	1.7 × 10^4^	8.7 × 10^3^	4.4 × 10^3^	2.2 × 10^3^	1.1 × 10^3^	5.4 × 10^2^

### Introduced cost by DU

In the following section, the computational and communication costs for each entity in the DU have been presented in details.

In the DU, the user incurs a cost that is similar to the one in the D3U. As shown in Algorithm 15, to insert data block, the user first encrypts the data block using the LiSHE scheme. Then it is sent the data block to the leader provider to store in its M2T and for it to distribute its copies to other providers. Meanwhile, to modify the existing data block, the user first decrypts the data block, modifies, and then encrypts it again. In the data block deletion case, the user does not incur a cost. Furthermore, the user computes a tag for the updated data block as in modification and insertion cases. The DIA-MTTP supports a data update with data deduplication. Therefore, the user does not generate a tag for the updated data block in the case that it is duplicated, as shown in Algorithm 17.

**Fig 34:**
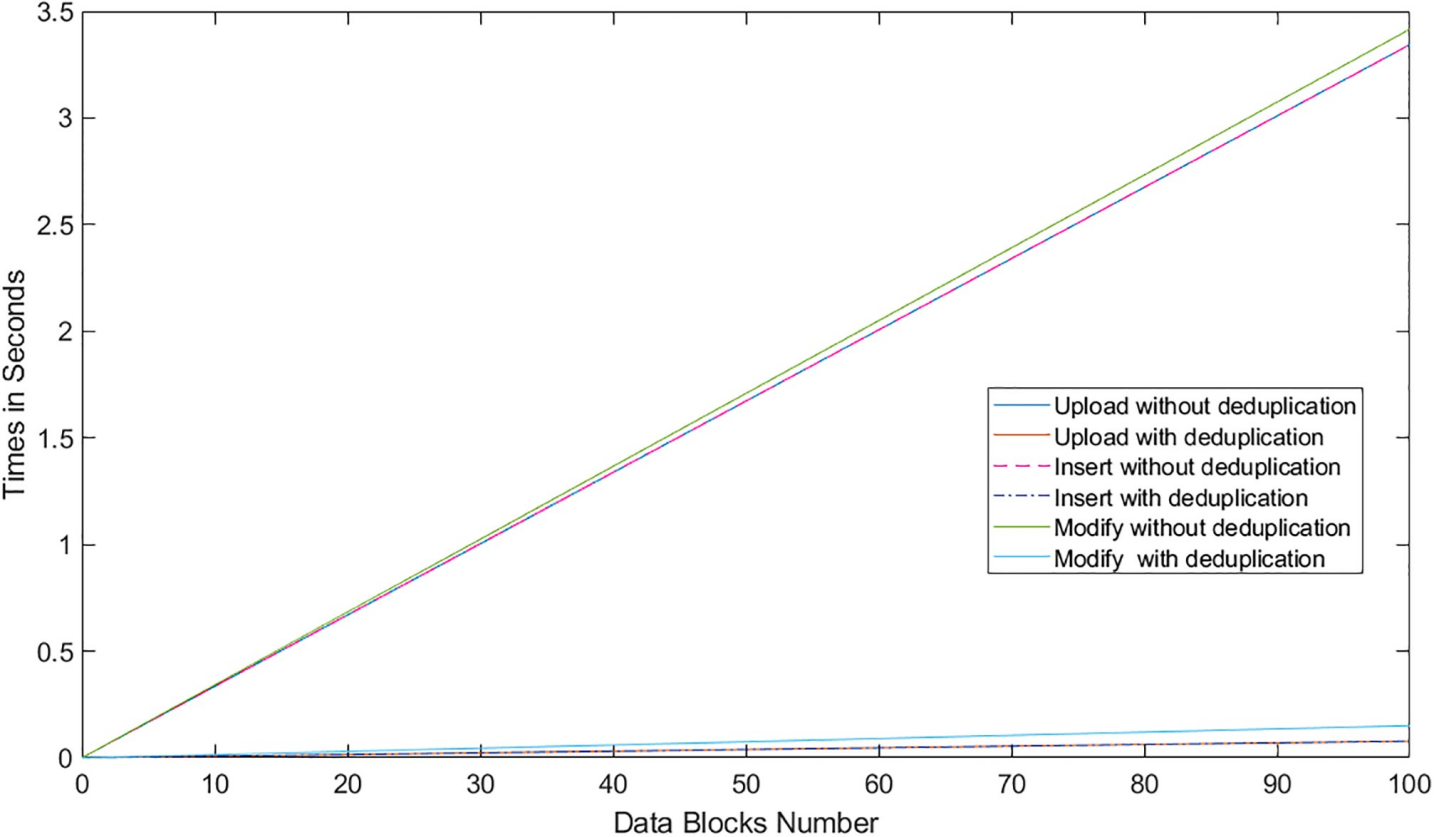
Computational cost of the user in DU vs the number of data blocks.

Fig 34 compares the computational cost of the user in DU against the data block number in three cases: data uploading (the first time a data file is being uploaded), data insertion and data modification. The data blocks are updated individually as a part of non-batch operations. Their costs increase linearly with the data block number. However, the costs in all cases (upload, insert, and modify) can be reduced by applying data deduplication. From the results of the figure, we can see that the data modification without a deduplication case can incur a higher cost compared to the cost of the modification with the deduplication and the data uploading and inserting with/without deduplication. The costs that are introduced in the uploading and insertion of the data block, with and without deduplication, are identical. Furthermore, the cost of the modification with the deduplication (where only the decryption and encryption operations are performed) is much less than the uploading/insertion of the data block without deduplication (where is in addition to the encryption operation, the tag of the data block should be generated). This means that the highest cost for the user comes from the tag generation operation. Thus this shows how to integrate the data update and data deduplication to help to reduce the cost for the user.

The providers in the DU incur a cost that is similar to one in the D3U. The leader provider receives the updated data from the user and checks the duplication. In other words, the leader provider performs data deduplication to eliminate any redundant data blocks by generating hash values for the updated data blocks, comparing them with hash values of the outsourced data. Then, the leader provider sends the result to the user. Therefore, the computational cost for the leader provider in the DU is based on the number of data blocks. The more updated data blocks there are, the higher the cost. The non leader providers, like in the D3U protocol, receive the updated data and their tags from the leader provider. Therefore, the computational cost for the non leader provider is negligible compared with the cost to the leader provider.

The TPAs in the DU perform the same functions as in the D3U protocol. The L-TPA receives the *En*_*IDTag* of the updated data block and stores it in its M2T. Therefore, the L-TPA does not execute any costly operations and computational cost in the DU can be negligible. Regarding the computational cost of the non leader TPAs, they are not involved in the DU so they have not introduced a cost.

Regrading to the communication cost of the user in DU, the user sends three messages, two to the leader provider, Req.DataUpdating and Req.TagsUpdating, and one to the L-TPA, Req.En_IDTagOfUpdatedDataUploading. The three messages are similar to Req.L2DataDeduplication, Req.TagsUploading and En_IDTagsUploading in the D3U protocol. In Req.DataUpdating, the user sends the updated data block to store in the PCSes, while in the Req.TagsUpdating, the associated tags, i.e. *DBTag* and *DBTagTag*, are sent. The *En*_*IDTag* of the updated data block is sent in the Req.En_IDTagOfUpdatedDataUploading. In data block deletion, the user does not need to send the data blocks or their tags, only their positions in a given data file. Furthermore, by applying the data deduplication, it can save the user communication. The user can be released from sending the tags in a case where the updated data blocks are duplicated.

The leader provider sends the Req.DataTagsUpdating to each provider for them to update their storage (M2Ts) while the Res.TagsUpdating is sent to the user. The former message includes the updated data block and its associated tags, while the latter message includes the acknowledgement to confirm that the outsourced data are updated on all PCSes. Therefore, the communication cost incurred by the leader provider in the DU, CommLPCSDUCost, is (n-1) × Req.DataTagsUpdating + Res.TagsUpdating. As the acknowledgement can be respected using one bit, 1, the CommLPCSDUCost is based on the size of the data block and their tags as well as the number of PCSes.

Each non leader provider only sends one message, i.e. Res.DataTagsUpdating to the leader provider. This message is similar to Res.DataTagUploading in the D3U protocol. It includes the acknowledgement in order to confirm that outsourced data have been updated in the PCS.

The communication costs that are introduced to the L-TPA and non leader TPAs are similar to the costs in the D3U. Only the L-TPA stores the *En*_*IDTag* and then sends the Res. En_IDTagOfUpdatedDataUploading as a response to confirm that the *En*_*IDTag* has been updated correctly. One bit is used for its representation. The communication cost at the L-TPA in the DU, the CommLTPADUCost, is negligible and constant regardless of the number of updated data blocks.

### Storage overhead for the entities in the DIA-MTTP

To measure the storage cost incurred by each entity in the DIA-MTTP system, we counted the number of kilobytes (KB) that the entity stored locally.

The user uploads the data along with the associated tags, i.e. *DBTags* and *DBTagTags* to the PCSes and *En*_*IDTags* in the L-TPA for the public verification. He/she keeps *IDTags* locally to use in the private verification. Thus, the storage overhead incurred by the user for each uploaded data file, the UserStorageCost, is dependent on the number and size of the *IDTags* for one data file. The *IDTags* are generated using the algebraic signature scheme. The UserStorageCost is *K* × |*IDTag*| = *K* × |*m*|. The UserStorageCost increases as the number of tags and their length increase.

In the DIA-MTTP, each provider has two sets of tags: *DBTags* and *DBTagTags*. Thus, the provider’s storage cost, PCSStorageCost, which is *K* × (|*DBTag*| + |*DBTagTag*|) = *K* × |*m*| + |*G*_1_|. On the other hand, the leader provider has an additional set, hash values of the data blocks set, to save on its computational cost. This set is used for performing D3L2 in the L2DataDeduplication protocol. Thus, the storage cost of the leader provider, LPCSStorageCost, is *K* × (|*DBTag*| + |*DBTagTag*| + |*DBHash*|). Without the hash values being set, the leader provider should compute the hash values for the outsourced data blocks for each uploading time. This can lead to an increase in the computation cost for the leader provider as the number of blocks increases.

Regarding the storage overheard incurred by the TPAs in the DIA-MTTP, the TPA stores *En*_*IDTags*. The tag set is uploaded to the TPAs to save the computational and communication costs on the user side. Without the *En*_*IDTags*, the user should generate *En*_*IDTags* for each verification and send them to the TPAs or store them locally. In other words, keeping the *En*_*IDTags* with the TPAs can release the user from being involved in the public verification.

By applying the collaborative verification approach in the DIA-MTTP, it can save on the storage cost incurred by the TPAs. The L-TPA only keeps the *En*_*IDTags*. For each verification, an aggregated value of the requested the *En*_*IDTags* that are associated with the challenging data blocks, is sent to each TPA. As it is an aggregated value, it does not burden the L-TPA communication cost regardless of the number of requested *En*_*IDTags*. Thus, the storage cost for the L-TPA, LTPAStorageCost, is *K* × |*En*_*IDTag*|. Without this approach, each TPA, along with the L-TPA, should keep the *En*_*IDTags*. This can lead to increasing the storage cost on the TPAs side, i.e. *K* × *n* × |*En*_*IDTag*|, where *K* is the total data block number, and *n* is the TPAs number in the system.

**Fig 35:**
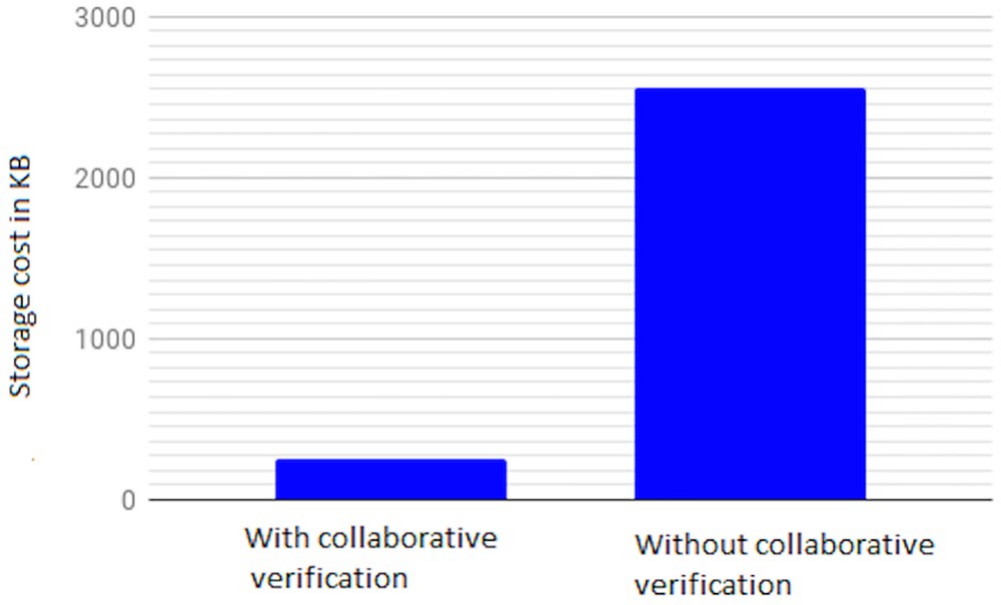
Storage cost at the TPAs: With/without the collaborative verification approach. (10 TPAs, |*n*^2^| = 0.256 KB).

**Fig 36:**
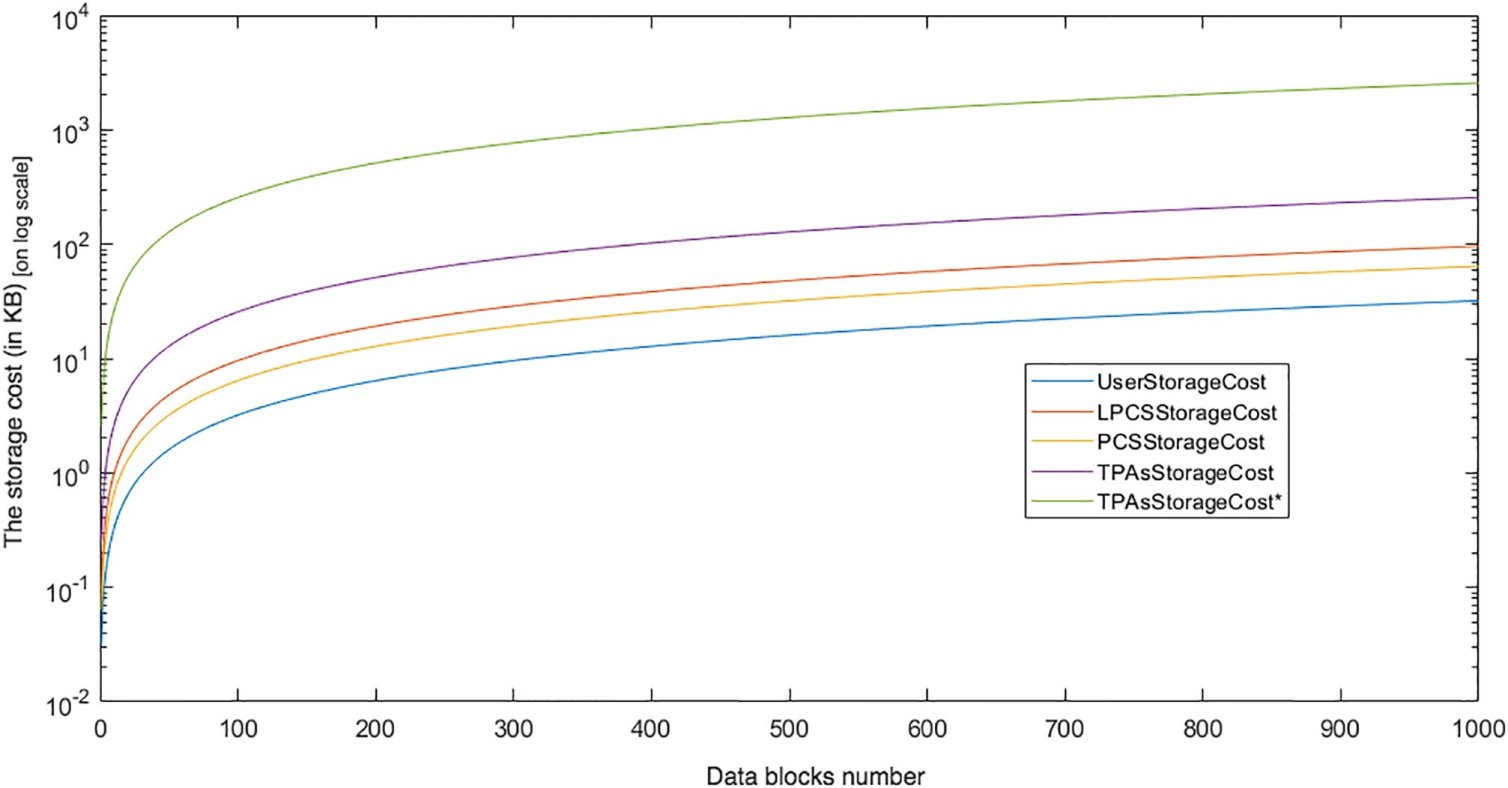
Storage cost for the DIA-MTTP entities with a different number of data blocks. (10 TPAs, |*m*| = |*G*_1_| = 0.032 KB, |*n*^2^| = 0.256 KB, * without the collaborative verification approach).

Fig 35 compares the storage costs at the point of the TPAs (leader and non leaders) with/without the collaborative verification approach. The figure shows how collaborative verification can succeed in saving on the storage cost at TPAs side. With the collaborative verification approach, the storage cost is about 10% of the cost without the approach. The DIA-MTTP can incur of a less storage cost by keeping the *En*_*IDTags* with the L-TPA. Fig 36 compares the storage costs of the DIA-MTTP entities against the number of data blocks. The costs increase as the number of data blocks increases. Based on the results in the figure, we can see that the user incurred the lowest cost.

**Fig 37:**
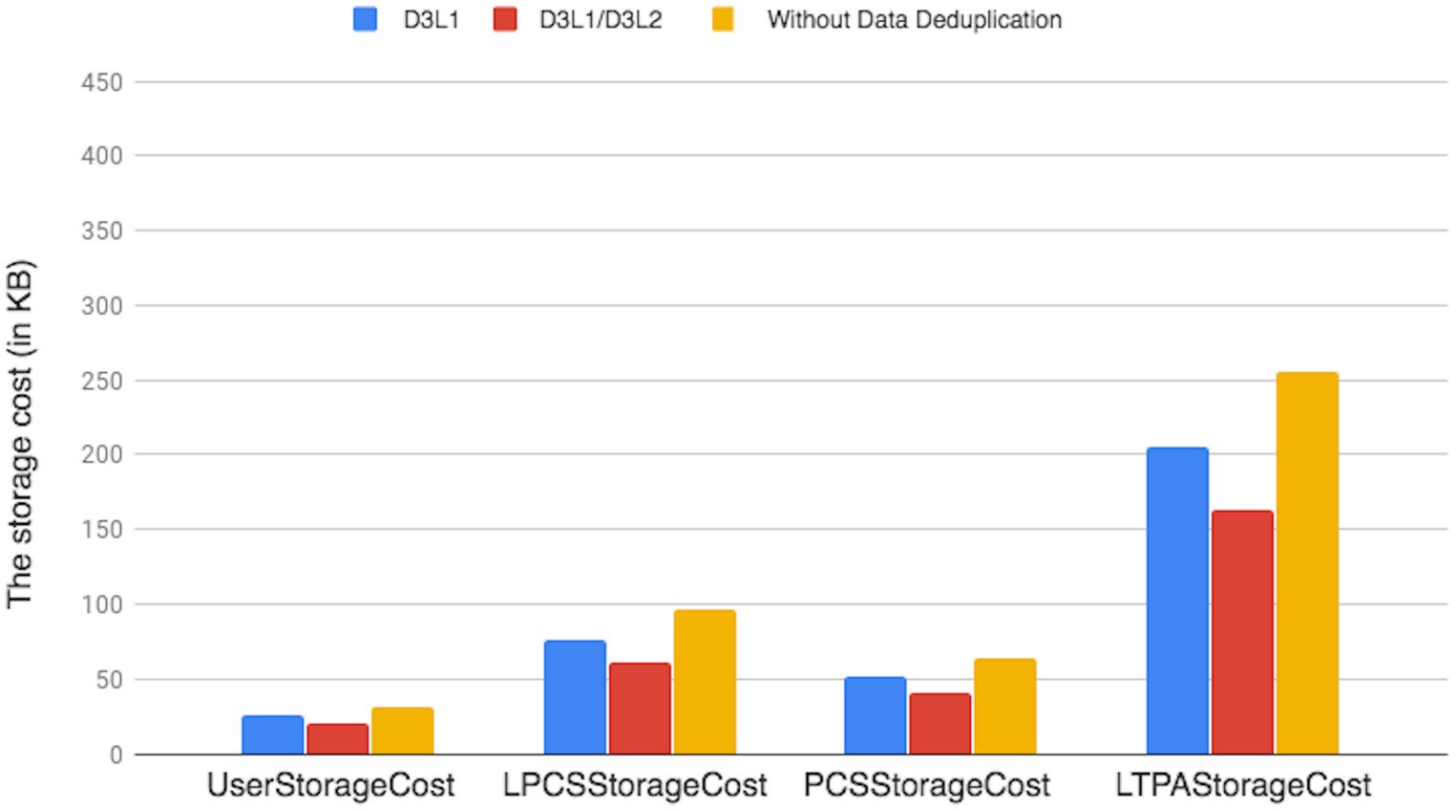
Storage cost for each entity in the DIA-MTTP: With/without data deduplication. (The redundancy data rate 20%).

As the data deduplication is applied in the DIA-MTTP, it can minimise the storage costs. Thus, the UserStorageCost, PCSStorageCost and LTPAStorageCost can be reduced by (*K* − *d*2) × |*IDTag*|, (*K* − *d*2) × (|*DBTag*| + |*DBTagTag*|) and (*K* − *d*2) × |*En*_*IDTag*|, respectively. Fig 37 compares the storage cost for each entity of the DIA-MTTP with and without data deduplication. It shows that when using the two levels of data deduplication (D3L1 and D3L2), it can save more of a cost compared with using only D3L1.

We compared the DIA-MTTP to the related works. Table 8 presents a summary of the functional, security and reliability properties comparison. The table shows that in compression with the existing DIA, the DIA-MTTP can satisfy all of the requirements. In terms of the functional requirements, the DIA-MTTP supports data deduplication at the block-level, while the existing works apply the data deduplication at the file-level. The DIA-MTTP uses data deduplication in data updating cases as well as when data uploading. For the security requirements, the DIA-MTTP can address TPA attacks, in addition to provider attacks. The DIA-MTTP uses multiple PCSes and multiple TPAs to address the data recovery and elasticity requirements.

**Table 8:** Comparing the DIA-MTTP with existing DIAs against the functional, security and reliability requirements in requirement specification section.

Works	F1	F2	S1	S2	S3	R1	R2	Tagging Methods	Data Structure
Ateniese_1 [7]	No	No	Yes^	-	No	Yes+	No	Hashing based Method	-
Chen [8]	No	No	Yes^	-	No	No	No	AS based Method	-
Sookhak [9]	No	Yes	Yes^	-	No	No	No	AS based Method	MIHT
Zhang [10]	No	Yes	Yes^	-	No	No	No	MAC based Method	BUT
Luo [35]	No	Yes	Yes^	No	No	No	No	AS based Method	IHT
Xu [11]	No	No	Yes^	No	No	No	No	HomMAC based Method	-
Krishra et al. [39]	No	No	Yes	No	No	No	No	Symmetric Encryption based Method	-
Ateniese_2 [12]	No	No	Yes	No!!	Yes	Yes+	No	RSA Method	-
Erway [14]	No	Yes	Yes	No!!	Yes	Yes+	No	RSA based Method	RASL
Hanser [15]	No	No	Yes	No	Yes	Yes+	No	ECDSA based Method	-
Li_1 [16]	No	Yes	Yes^	No	Yes	No	No	BLS based Method	IHT
Liu_1 [17]	No	Yes	Yes^	No	Yes	No	No	BLS based Method	MHT
Wang [18–20]	No	Yes	Yes^	No!!	Yes	No	No	BLS based Method	MHT
Tian [40]	No	Yes	Yes^	No!!	Yes	No	No	BLS based Method	DHT
Yang [21]	No	Yes	Yes^	No	Yes	No	No	BLS based Method	IHT
Li_2 [41]	No	Yes	Yes^	No	Yes	No	No	BLS based Method	LBT
Liu_2 [56]	No	No	Yes^	No	Yes	Yes++	No!!	BLS based Method	-
Abo alian_1 A [23]	No	No	Yes	No!!	Yes	Yes++	No	BLS based Method	-
Abo alian_2 [24]	No	Yes	Yes	No!!	Yes	Yes++	No	BLS based Method	RASL
Curtmola [25]	No	No	Yes	No	Yes	Yes++	No	RSA based Method	-
Yuan [26]	Yes!	No	Yes^	No	Yes	No	No	BLS based Method	-
Li_3 [27]	Yes!	No	Yes	No!!	Yes	No	No	BLS based Method	-
Ma [28]	Yes!	Yes	Yes	No!!	Yes	No	No	BLS based Method	MHT
He [29]	Yes!	No	Yes	No!!	Yes	No	No	BLS based Method	-
Liu_3 [22]	Yes!	No	Yes	No!!	Yes	No	No	BLS based Method	-
Abbdal [30]	No	Yes	Yes	No!!	Yes	No	Yes*	ECDSA based Method	MHT
Saxena [31]	No	No	No	No	No	No	Yes*	AS based Method	-
Jin [32]	No	Yes	Yes^	No	Yes	No	No	BLS based Method	Index Switcher
Yang_2 [21]	No	Yes	Yes^	No	Yes	Yes+++	Yes**	BLS based Method	IHT
Ni [13]	No	No	Yes^	No	Yes	Yes+++	Yes**	RSA based Method	-
Zhu [33]	No	No	Yes^	No!!	Yes	Yes+++	Yes**	BLS Method	-
Liu_4 [34]	No	Yes	Yes^	No!!	Yes	Yes+++	Yes**	BLS based Method	MHT
DIA-MTTP	Yes	Yes	Yes	Yes	Yes	Yes+++	Yes	TOD Method	M2T

(^no data confidentiality preservation, *! file-level deduplication*, *!! data confidentiality preservation*, *+ encoding method*, *+ + multiple replicas at one PCS*, *+ + + multiple replicas at multiple PCSes*, * one PCS and Multiple TPAs, ** multiple PCSes and one TPA)

For the elasticity property, the DIA-MTTP can provide it more effectively. The non leader TPAs do not keep any data locally, only the L-TPA send the aggregated value of *En*_*IDTag* each verification time. This means that it is easy to add a new TPA. Furthermore, in the DIA-MTTP, identical data replicas are used. The user does not incur any overheads in terms of computational and communication costs if a new PCS is added. The leader provider can send a copy of the outsourced data and their associated tags to the PCS. This means that unlike existing works, the user in the DIA-MTTP does not need to re-process the data to generate a new replica and its associated tags, nor does it needs to communicate with the new PCS to upload them.

Table 9 presents a summary of the computational, communication and storage complexities of the DIA-MTTP and existing works. For the computational and communication costs that are introduced to the user for uploading, the Curtmola CenPub-DIA, Saxena CenPub-DIA and the DIA-MTTP are most efficient compared with other works. Unfortunately, Curtmola CenPub-DIA and Saxena CenPub-DIA do not address the TPA attacks, as shown in Table 8. The TPA should be entirely honest. Furthermore, they do not support data deduplication and dynamic data like the DIA-MTTP.

**Table 9:** Comparing the DIA-MTTP with existing works against the efficiency requirements in section.

Works	Computational Overhead			Communication Overhead			Storage Overhead (P7)+		
	P1!	P2!!	P3*	P4 +	P5 at Provider	P6 at User and Provider	User	Providers	TPAs
Ateniese_1 [7]	O(*NT* × *T*)	O(1)	-	O(*NT*)	O(1)	-	-	O(*NT*)	-
Chen [8]	O(*NT* × *T*)	O(1)	-	O(*NT*)	O(1)	-	-	O(*NT*)	-
Sookhak [9]	O(*K*)	O(1)	O(*DT*)	O(*K*)	O(1)	O(*DT*)/ O(1)	O(*K*)	O(*K*)	-
Zhang [10]	O(*K*)	O(1)	O(1)	O(*K*)	O(1)	O(1)	-	O(*K*)	-
Luo [35]	O(*K*)	O(1)	O(*DT*)	O(*K*)	O(1)	O(*DT*)/ O(1)	-	-	O(*K*)
Xu [11]	O(*K*)	O(1)	-	O(*K*)	O(1)	-	-	-	O(*K*)
Krishra et al. [39]	O(*K*)	O(1)	-	O(*K*)	O(1)	-	-	-	O(*K*)
Ateniese_2 [12]	O(*K*+ *E*)	O(1)	-	O(*K*+ *E*)	O(1)	-	-	O(*K*+ *E*)	-
Erway [14]	O(*K*+ *E*)	O(*NP* × *C*)	O(*NP*)	O(*K*+ *E*)	O(*NP* × *C*)	O(1)/O(*NP*)	-	O(*K*+ *E*)	-
Hanser [15]	O(*K*+ *E*)	O(1)	-	O(*K*+ *E*)	O(1)	-	-	O(*K*+ *E*)	-
Li_1 [16]	O(*S* × *K*)	O(1)	O(*DT*)	O(*K*)	O(1)	O(*DT*)/ O(1)	-	O(*K*)	-
Liu_1 [17]	O(*S* × *K*)	O(*NP* × *C*)	O(*NP*)	O(*K*)	O(*NP* × *C*)	O(1)/O(*NP*)	-	O(*K*)	-
Wang [18–20]	O(*K*)	O(*NP* × *C*)	O(*NP*)	O(*K*)	O(*NP* × *C*)	O(*NP*)	-	O(*K*)	-
Tian [40]	O(*K*)	O(1)	O(1)	O(*K*)	O(1)	O(1)/O(1)	-	O(*K*)	O(*K*)
Yang_1 [21]	O(*S* × *K*)	O(1)	O(*DT*)	O(*K*)	O(1)	O(*DT*)/ O(1)	-	O(*K*)	O(*K*)
Li_2 [41]	O(*K*)	O(*NP* × *C*)	O(1)	O(*K*)	O(*NP* × *C*)	O(1)	-	O(*K*)	-
Liu_2 [56]	O(*R* × *K*)	O(1)	-	O(*T*)	O(1)	-	-	O(*R* × *K*)	-
Abo alian_1 [23]	O(*R* × *K*)	O(1)	-	O(*K*)	O(1)	-	-	O(*R* × *K*)	-
Abo alian_2 [24]	O(R × K)	O(NP × C)	O(NP)	O(K)	O(NP × C)	O(1)/O(NP)	-	O(*R* × *K*)	-
Curtmola [25]	O(*R* × *K*)	O(1)	-	O(*R* × *K*)	O(1)	-	-	O(*R* × *K*)	-
Yuan [26]	O(*K*)	O(1)	-	O(*R* × *K*)	O(1)	-	-	O(*R* × *K*)	-
Li_3 [27]	O(*K*)	O(1)	-	O(*R* × *K*)	O(1)	-	-	O(*R* × *K*)	-
Ma [28]	O(*K*)	O(*NP* × *C*)	O(*NP*)	O(*K*)	O(*NP* × *C*)	O(1)/O(*NP*)	-	O(*K*)	-
He [29]	O(*K*)	O(1)	-	O(*K*)	O(1)	-	-	O(*K*)	-
Liu_3 [22]	O(1)	O(1)	-	O(1)	O(1)	-	-	O(1)	-
Abbdal [30]	O(*K*)	O(1)	O(*NP*)	O(*K*)	O(*K*)	-	-	O(*K*)	-
Saxena [31]	O(1)	O(1)	-	O(*K*)	O(1)	-	-	O(*K*)	-
Jin [32]	O(*K*)	O(1)	O(1)	O(*K*)	O(1)	O(1)/O(1)	-	O(*K*)	-
Yang_2 [21]	O(*K*)	O(*n*)	O(*DT*)	O(*n* × *K*)	O(*n*)	O(*n* × *DT*)/O(1)	-	O(*n* × *K*)	O(*K*)
Ni [13]	O(*K*)	O(1)	-	O(*K*)	O(*n*)	O(1)/O(*NP*)	-	O(*n* × *K*)	-
Zhu [33]	O(*K*)	O(1)	-	O(*K*)	O(1)	-	-	O(*n* × *K*)	-
Liu_4 [34]	O(*K*+ *n*)	O(*NP* × *C*)	O(*NP*)	O(*K*+ *n*)	O(*NP* × *C*)	O(1)/O(*NP*+ *n*)	-	O(*n* × (*K*+ 1))	-
DIA-MTTP	O(*d*2)	O(1)/O(*C*)	O(1)	O(*d*2)	O(1)/O(*C*)	O(1)	O(*d*2)	O(*n* × *d*2)	O(*d*2)

(*! Cost of tag generation*, *!!Cost incurred at a verifier in private and public, receptively*, * Cost of updating one data block by the user, *+ Only tags of one data file*)

For storage cost complexities, we only considered the number of tags of one data file. As shown in the table, the DIA-MTTP can introduce more storage overheads in comparison with the existing DIAs on a part of the user. The cost is used for enhancing the DIA’s security. However, through the data deduplication, this cost can be reduced.

## Conclusion

This paper has proposed and evaluated a novel DIA framework. The framework deploys the TOD method to support a two-level approach to integrity verification and data deduplication, and uses a multi-entity hierarchical structure, the principle of the separation of duties and collaborative verification to counter collusion attacks and to enhance data availability and an integrated approach to data updating and data deduplication to support dynamic data and to minimise costs imposed on the entities involved. The two-level approach can best balance the trade-off between protection strength and overhead cost and maximise the benefits of data deduplication. Furthermore, the novel data structure, M2T, has been proposed for supporting the data updating and facilitating the data deduplication.

Our future work includes how we can address the storage overhead costs at the user and the L-TPA. Additionally, how we can update the tags efficiently and securely in the case of their key is exposed. The DIA framework could be extended to provide the DIA for sharing data, where multiple users can access and update the same data in the PCS.

## Supporting information

S1 File Requirements of an effective, secure, reliable and efficient DIA. (PDF)

S2 File M2T and its operations. (PDF)

S3 File DIA-MTTP algorithms. (PDF)

S4 File Correctness of LoA1DV and LoA2DV protocols. (PDF)

S5 File Complexities of computational and communication cost in DIA-MTTP. (PDF)
