# Supplementary material for: A novel approach to data integrity auditing in PCS: Minimising any Trust on Third Parties (DIA-MTTP)
Source: PLoS One. 2021 Jan 7;16(1):e0244731. doi: 10.1371/journal.pone.0244731 (PMC7790547; doi:10.1371/journal.pone.0244731)
Supplement: S1 File — (PDF) [file pone.0244731.s001.pdf]

Requirements of an Effective, Secure, Reliable and Efficient DIA

The requirements of an effective, secure, reliable and efficient DIA can be classified into four groups, functional, security, reliability and performance requirements.

(F) Functional Requirements:
(F1) Support Data/Tags Deduplication: It should eliminate redundant data over one file or multiple files and their associated tags. This requirement is intended for reducing computational and storage overheads as well as saving a bandwidth.
(F2) Support Dynamic Data/Tags: It should allow a PCS user (i.e. the data owner) to update (i.e. insert, delete or modify) his data at PCS. The PCS user can perform dynamic operations on his outsourced data such as insert, delete, and modify while assuring the integrity of outsourced data files. Furthermore, it should update data without the need to recompute other unrelated tags as well as keep the data are non-duplicated after each update.

(S) Security Requirements:
(S1) Resistance of PCS providers cheating: It should resist cheating attacks by dishonest PCS providers, i.e., forgery, replace, and replay attacks, and prevent data confidentiality breach.
(S2) Resistance of TPAs cheating: It should resist cheating attacks by dishonest TPAs, i.e., collusion and frame attacks, and prevent data confidentiality breach.
(S3) Resistance of PCS users cheating: It should resist cheating attacks by dishonest PCS users, i.e., a repudiation attack in tag generation or data updating.

(R) Reliability Requirements:
(R1) Data Recovery: It should mitigate risks of loss or modification outsourced data and their tags. In the case of losing the data, the PCS user can recover his data and their tags, and without the need to preprocess data and generate new tags.
(R2) Elasticity: It should give the ability to cope with a dynamic number of PCSes and TPAs in the DIA system. As the number of the PCSes can be variable for some reasons, e.g. shout down or stop, the number of TPAs

can be scaled up or down based on the number of PCSes. In other words, if the service of one PCS outages for any reason, consequently, the number of TPAs can be scaled down. On the other hand, if one TPA goes down, too, a new TPA can be added to handle the situation. It can provide a more resilient system, i.e. available and durable system.

(P) Performance Requirements:
(P1) Minimizing Data Uploading Computational Cost: The computational cost incurred in data processing, e.g. tag generation, should be as low as possible.
(P2) Minimizing Data Verification Computational Cost: The computational cost incurred in data verification, i.e. proofs generation and proofs correctness verification, should be as low as possible.
(P3) Minimizing Data Updating Computational Cost: The computational cost incurred in data updating, i.e. insert, delete or modify, should be as low as possible.
(P4) Minimizing Data Uploading Communication Cost: The communication cost incurred in data uploading should be as low as possible.
(P5) Minimizing Data Updating Communication Cost: The communication cost incurred in data updating should be as low as possible.
(P6) Minimizing Data Verification Communication Cost: The communication cost incurred in a data verification should be as low as possible.
(P7) Minimizing Storage Overhead Cost: The storage cost incurred at DIA-ETTP entities, i.e. PCS users, PCS providers and TPAs, should be as low as possible. Efforts should be made to reduce a PCS user cost as much possible.
