# Supplementary material for: A novel approach to data integrity auditing in PCS: Minimising any Trust on Third Parties (DIA-MTTP)
Source: PLoS One. 2021 Jan 7;16(1):e0244731. doi: 10.1371/journal.pone.0244731 (PMC7790547; doi:10.1371/journal.pone.0244731)
Supplement: S2 File — (PDF) [file pone.0244731.s002.pdf]

Multiple Mapping Tables (M2T) operations

M2T consists of three tables; a UserFile, a NonDuplicatedDB/Tag and a Linker. The UserFile shows all of the data files that are owned by the user. The NonDuplicatedDB/Tag shows all of the non-duplicated data blocks and/or tags present among the data files. The Linker shows the linkages between the data blocks, the data files and the non-duplicated data blocks and their respective tag values. As mentioned above, each entity in the DIA-ETTP has its own M2T, i.e. User-M2T, PCS-M2T and TPA-M2T. The content of the UserFile is identical between User-M2T, PCS-M2T and TPA-M2T. The contents of the NonDuplicatedDB/Tag and the Linker from among the three types are different based on the type of tag and if the data blocks values are stored by an entity that manages the M2T or not. The UserFile consists of two columns; the ID of a data file (*FileID*) and the total DBs number in the data file (*DBsTotalNumber*). The NonDuplicatedDB/Tag in User-M2T consists of two columns, namely the ID of the $IDTag$ (*IDTagID*) and the $IDTag$ value (*IDTagValue*). Regarding TPA-M2T, the NonDuplicatedDB/Tag is similar to the one in User-M2T. However, the IDs of the En_IDTags and their values are stored as an alternative to the IDs of $IDTags$ and their values. The NonDuplicatedDB/Tag in PCS-M2T consists of four columns: (1) an ID of the data block (*DBID*), (2) the DB value (*DBValue*), (3) its associated $DBTag$ value (*DBTagValue*) and (4) its associated $DBTagTag$ value (*DBTagTagValue*). The Linker table consists of three columns: (1) the Index of DB among the data file (*DBIndex*), (2) the ID of the file in which its value is one of the IDs in the UserFile, (*FileID*), and (3) the ID of *IDTag*, *IDDB*, or *En_IDTag* in the NonDuplicatedDB/Tag of User-M2T, PCS-M2T or TPA-M2T, respectively.

To update the data, there are three operation types, i.e. a data block insertion, a data block modification and a data block deletion. Their details have been given below. Figures 1, 2 and 3, show how PCS-M2T is used and updated when the data are uploaded (inserted), modified and deleted.

To insert a new data block into a file, one should first check if the data block is duplicated, i.e. Insertion Case 1 (Ins-Case_1) or not, i.e. Insertion Case 2 (Ins-Case_2) is done using the NonDuplicatedDB/Tag. Figure 1 shows that the PCS-M2T content changes when a new data block is inserted.

1. Ins-Case_1: In the case where the insertion of a data block is duplicated, the operations of the insertion are as follows: (1) insert a new row that is associated with the data block in the Linker, (2) link the data block to its associated data file using $FileID$ in the UserFile, and (3) link it to its

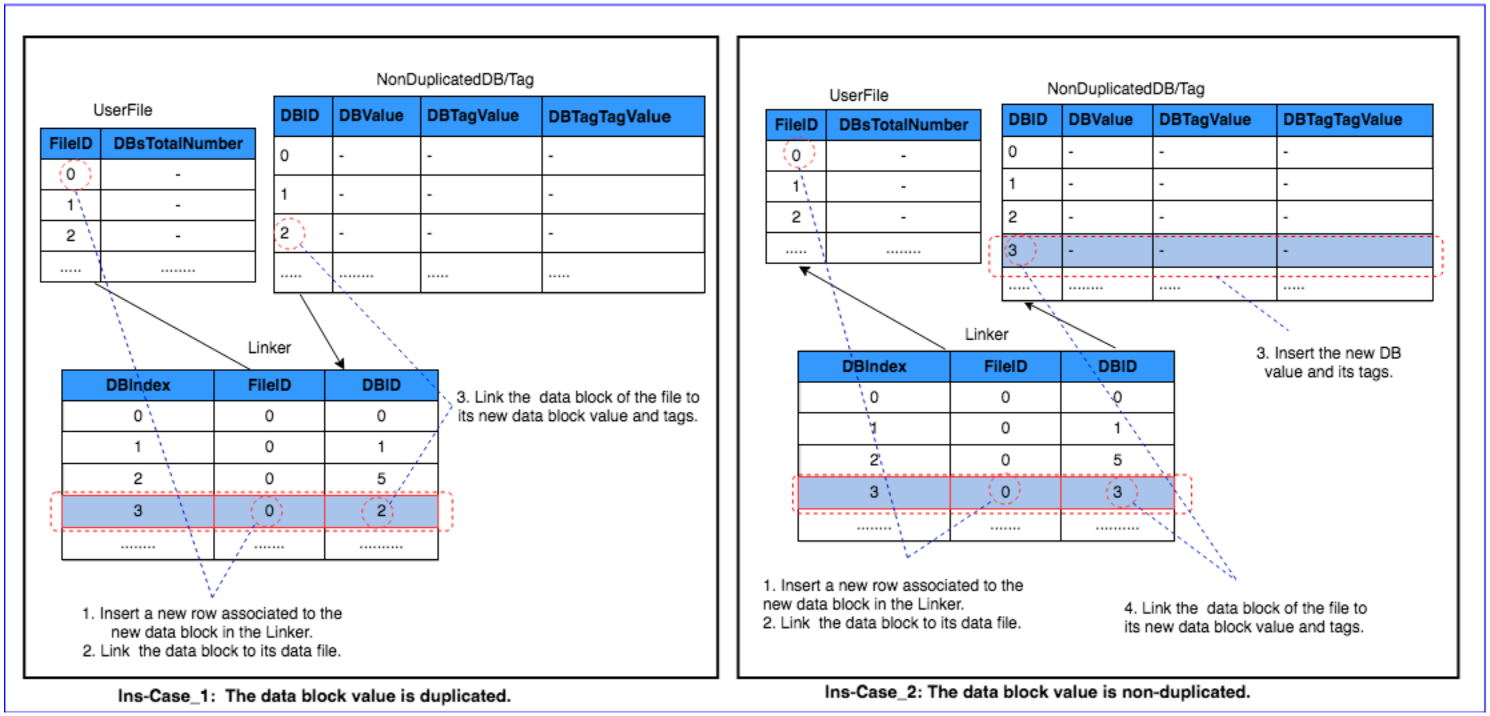

Figure 1: Data Insertion in PCS-M2T.

associated DB or tags values using $IDTagID$, $DBID$ or $En\_IDTagID$ in the NonDuplicatedDB/Tag in the case of using User-M2T, PCS-M2T or TPA-M2T, respectively.

2. Ins-Case_2: In the case of the inserting a data block that is non-duplicated, the operations of the insertion are as follows: (1) insert a new row that is associated with the data block in the Linker, (2) insert the values of its associated data block and/or tags in the NonDuplicatedDB/Tag, (3) link the data block to its associated data file using the $FileID$ of the data file in the UserFile, and (4) link the data block to its associated values regarding the non-duplicated data block or tags using their IDs in the NonDuplicatedDB/Tag.

To modify an existing data block, it should first be checked whether or not the value of the old version of the data block is associated with other data blocks as well as whether its new version value is duplicated or not using the Linker and the NonDuplicatedDB/Tag. There are four cases: (1) the old version of the data block is associated with other existing blocks and its new version is non-duplicated, i.e. Modification case 1 (Mod-Case_1), (2) the old version of the data block is associated with other existing blocks and its new version is duplicated, i.e. Modification case 2 (Mod-Case_2), (3) the old version of the data block is not associated with any existing blocks and its new version is non-duplicated, i.e. Modification case 3 (Mod-Case_3), and (4) the old version of the data block is not associated with any existing blocks and its new version is duplicated, i.e. Modification case 4 (Mod-Case_4). Figure 2 shows the content changes in PCS-M2T before and after an existing data block is modified.

1. Mod-Case_1: In the case where the old version of the data block is associated with other blocks and the new version is non-duplicated, the operations of the modification are as follows: (1) Insert the new version of DB and its tag values in the NonDuplicatedDB/Tag, and (2) Link the data block in the Linker with its new associated values concerning DB and/or the tags using their IDs in the NonDuplicatedDB/Tag.

2. Mod-Case_2: In the case where the old version of the data block is associated with other blocks and its new version is duplicated, the operations of the modification involve: linking the data block with its new associated DB and/or tag values using their ID in the NonDuplicatedDB/Tag.

3. Mod-Case_3: In the case where the old version of the existing data block is not associated with any existing blocks and the new version is non-duplicated, the operations of the modification are as follows: (1) delete the DB and/or tag values that are associated with the old version in the NonDuplicatedDB/Tag, (2) insert the new values for the DB and/or tags in the NonDuplicatedDB/Tag and (3) link the data block in the Linker to the DB and/or the tag values of the new version using their IDs in the NonDuplicatedDB/Tag.

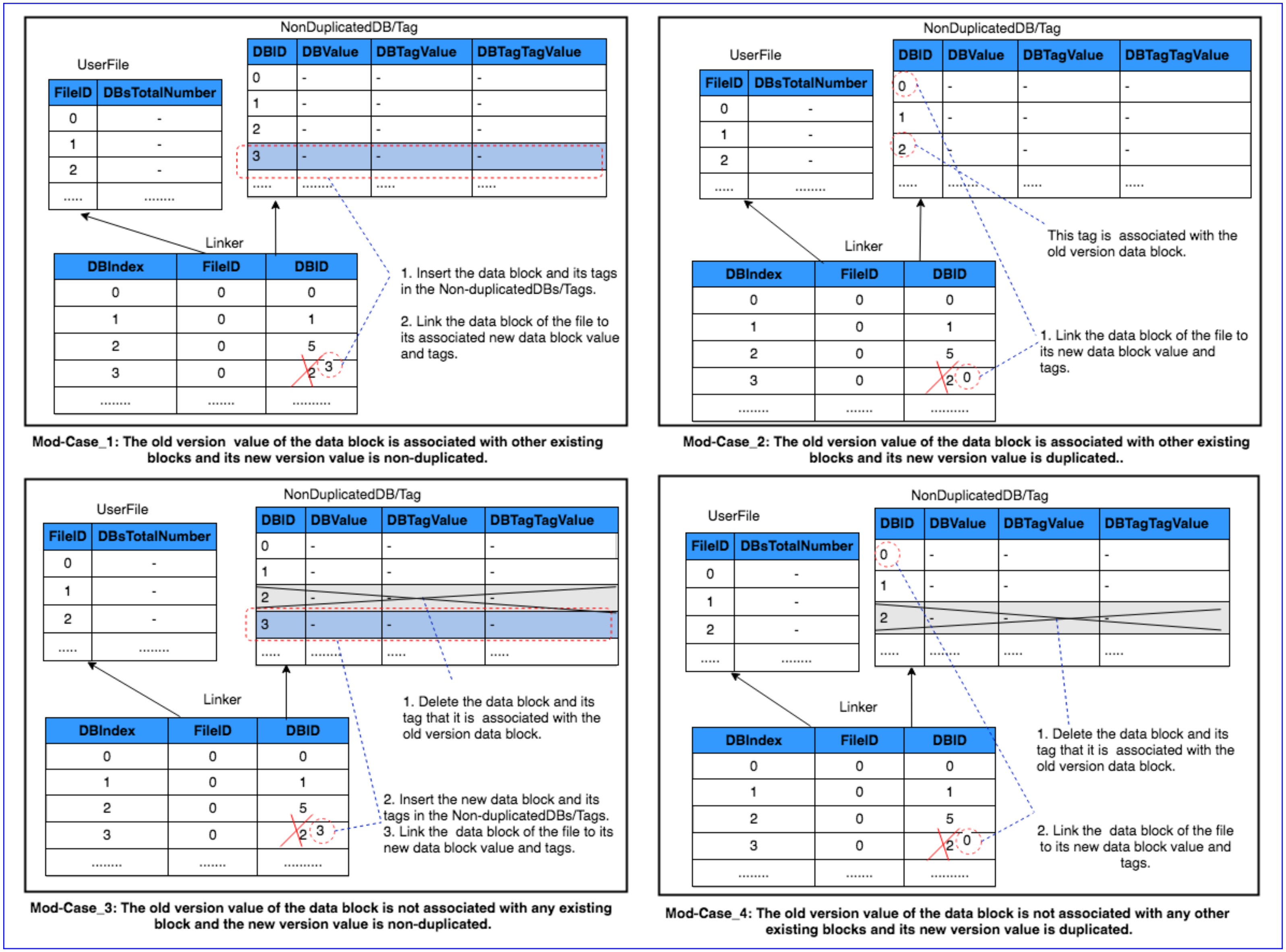

Figure 2: Data Modification in PCS-M2T.

4. Mod-Case_4: In the case where an old version of the data block is not associated with any other blocks and the new version is duplicated, the operations of the modification are as follows: (1) delete the DB and tag values that are associated with the old version in the NonDuplicatedDB/Tag and (2) link the data block in the Linker with its new associated data block and/or the tag values using their IDs in the NonDuplicatedDB/Tag.

To delete an existing data block in the file, it should first be checked using the Linker table to see if its value is associated with any other existing blocks as either in deletion case 1 (Del-Case_1) or deletion case 2 (Del-Case_2). Figure 3 shows the content changes in PCS-M2T before and after an existing data block is deleted.

1. Del-Case_1: To delete a data block that is associated with one of the other blocks, only the row that is associated with the data block in the Linker is deleted.

2. Del-Case_2: To delete a data block that is not associated with any other block, the operations of deletion are as follows: (1) delete the row that is associated with the deleted data block in the Linker, and (2) delete the values of the DB and/or tags that are associated with the data block in the NonDuplicatedDB/Tag.

The value of DBsTotalNumber that is associated with the updated data file should be increased or decreased by one in the case of either an insertion or deletion operation being performed, respectively.

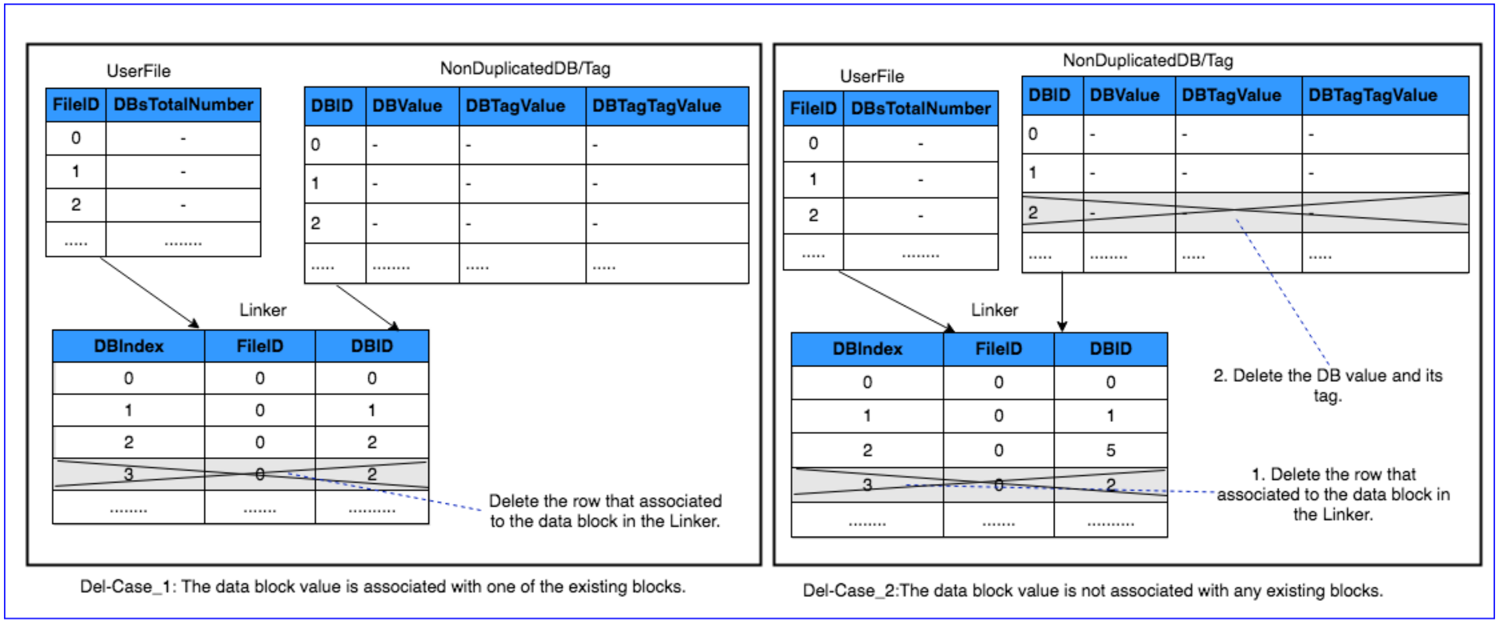

Figure 3: Data Deletion in PCS-M2T.
