# Supplementary material for: A novel approach to data integrity auditing in PCS: Minimising any Trust on Third Parties (DIA-MTTP)
Source: PLoS One. 2021 Jan 7;16(1):e0244731. doi: 10.1371/journal.pone.0244731 (PMC7790547; doi:10.1371/journal.pone.0244731)
Supplement: S3 File — (PDF) [file pone.0244731.s003.pdf]

DIA-MTTP Algorithms

Algorithm 1 FileSetUp

Input : DF, sk
Output: $D3L1R$

1. Divide a data file (DF) into K data blocks, $\{DB_i\}$, $0 \leq i < K$.

2. Set $L1$= $L2$=$D3L1R$=$\{DB_i\}$, $0 \leq i < K$.

3. Eliminate any additional identical data blocks among K data blocks, i.e. only keep one copy of any identical blocks. The output of this step is $d1$ non-duplicated data blocks, $\{DB_i\}$, $0 \leq$ i $< d1$.

   for $i = 0 \rightarrow L1.size\text{-}1$ **do**

       for $j = 1 \rightarrow L2.size\text{-}1$ **do**

           if $L1_i$==$L2_j$ **then**

               Set $D3L1R_{i+j}$= i // i= is an identifier of the data block that is identical to $L1_i$.

           end

       end

       Delete $L2_i$

   end

4. Encrypt each of $d1$ non-duplicated data blocks, $\{DB_i\}$ in $D3L1R$, using the LiSHE-Enc algorithm and key, sk, to produce a set of encrypted data blocks, $\{En\_DB_i\}$, $0 \leq$ i $< d1$.
   for $i = 0 \rightarrow K - 1$ **do**

       if $D3L1R_i$ *not in [0: K-1]* **then**

           Compute: En_DB_i = LiSHE-Enc($D3L1R_i, sk$)

       end

   end

Algorithm 2 L2DataDedup

Input : $D3L1R$, $ODBH$
Output: $D3L2R$, NDB, $NDBH$

1. RDB is a list of the received data blocks in $L1D3R$.
2. $D3L2R$, NDB, and $NDBH$ are empty lists.
3. **for** $i = 0 \rightarrow d1\text{-}1$ **do**

    Compute a hash of RDB_i, i.e. $HEn\text{-}DB_i$.

    for $j = 0 \rightarrow M - 1$ // *M is the total number of the outsourced data blocks in PCS,* **do**

        if $HEn\text{-}DB_i == ODBH_j$ **then**

            Set $D3L2R_i = j$ // j is an ID of data block in M2T that is identical to RDB_i.

            Stop the inner For

        end

    end

    if $D3L2R_i$ *is empty value* **then**

        Add RDB_i to NDB. // It means the data block is non-duplicated

        Add $HEn\text{-}DB_i$ to $NDBH$.

    end

   end

Algorithm 3 BlockTagGen

Input : $\{ En\_DB_i \}$, $0 \leq i < d1$, $User_{ID}$, x, υ, ppk_{En}, $D3L1R$, $D3L2R$
Output: $\{IDTag_i\}$, $\{En\_IDTag_i\}$, $\{DBTag_i\}$, $\{DBTagTag_i\}$, $0 \preceq i \prec d2$, $D3L1R'$
for $i = 0 \rightarrow d1 - 1$ **do**
  if $D3L2R_i$ *is empty value* **then**

1. Computes tags for En_DB_i, i.e. $IDTag_i$, $DBTag_i$, $DBTagTag_i$ and En_IDTag_i using TagGen algorithm with $User_{ID}$, x, υ, ppk_{En}.
2. Remove data block values in $D3L1R$, $D3L1R'$.

  end
end

Algorithm 4 FileTagSigGen

Input : K, $FileID$, $Pkey$
Output : $FileTagSig$

1. Compute a file tag $FileTag = FileID||K$.
2. Compute a hash value for the file tag:

$$FileTagH = H1(FileTag)$$

3. Compete a signature for $FileTag$ as follows:

$$FileTagSig = E_{Pkey}(FileTag||FileTagH) \quad (1)$$

//E is an encryption algorithm of one of public-key cryptography schemes, e.g. RSA, Pkey is a private key and Hash is a hash function, e.g. SHA256.

Algorithm 5 PubChalGen

Input : $FileTagSig$, $PuKey$
Output: $\{PubChall_j\}, 0 \leq j < n-1$, $AggProofNonceTag$

1. Verify $FileTagSig$ to recover FileTag as follows:

   1.1 Decrypt $FileTagSig$ using $PuKey$ and recover two values: $FileTag$ and $FileTagH$.

   1.2 Compute a fresh hash value, i.e. $FileTagH'$, for $FileTag$.

   1.3 **if** $FileTagH == FileTagH'$ **then**

   Set X=1.

   else

   Set X=0.

   end

2. **if** $X == 1$ **then**

   2.1 Choose randomly C-element subset of set K data blocks identifiers, $\{I_i\}$, $0 \leq i < C$.

   2.2 Choose for each I_i, a random value $ProofNonce_i \in Z_p$, $\{ProofNonce_i\}, 0 \leq i < C$.

   2.3 Compute an aggregated value of $\{ProofNonce_i\}$, i.e. $AggProofNonce$, as follows:

$$AggProofNonce = \sum_{i=0}^{C-1} ProofNonce_i \tag{2}$$

   2.4 Compute a tag of $AggProofNonce$ using AS scheme, i.e. $AggProofNonceTag$.

   2.5 Choose randomly value for each PCS as nonce, $\{PCSNonce_j\}$, $PCSNonce_j \in Z_p$.

   2.6 Compute tags for the set $\{PCSNonce_j\}$ using AS scheme, i.e. $\{PCSNonceTag_j\}$.

   2.7 **for** $j = 0 \to n-2$ **do**

   Set $PubChall_j$= $\{$ $\{I_i\}||\{ProofNonce_i\}$ $||PCSNonce_j||PCSNonceTag_j||FileID$ $\}$

   end

   else

   Stop

   end

Algorithm 6 PubProofsGen

Input : $PubChall_j$, $\{En\text{-}DB_i\}$, $\{DBTag_i\}$, $\{DBTagTag_i\}$, $0 \leq$ i <C
Output: $PubDBProof_j, PubDBTagProof_j, PubDBTagTagProof_j$

1. Compute:
$$PubDBProof_j = \sum_{i=0}^{C-1}(En\text{-}DB_i + ProofNonce_i) \quad (3)$$

2. Compute:
$$PubDBTagProof_j = \{PubDBTagProof_{ji}\}, 0 \leq i < C \quad (4)$$
Where $DBTagProof_{ji}$= $DBTag_i$ + $PCSNonceTag_j$

3. Compute:
$$PubDBTagTagProof_j = (\prod_{i=0}^{C-1} DBTagTag_i^{ProofNonce_i})^{PCSNonce_j} \quad (5)$$

Algorithm 7 DBProofTagGen

Input : $\{PubDBProof_j\}$, $0 \leq$ j $< n-1$, $\{En\_IDTag_i\}$, $0 \leq$ i $< C$
Output: $DBProofTag$, $AggEn\_IDTag$

1. Compare all the $PubDBProof$ values, i.e. $\{PubDBProof_j\}$, if they are equal, set Comparison Result= 1, otherwise set Comparison Result= 0.

2. **if** *Comparison Result== 1* **then**

   2.1 Compute a tag for $PubDBProof$, i.e. $DBProofTag$, using AS scheme.

   2.2 Aggregate value of En_IDTags, i.e. $AggEn\_IDTag$, that are associated with the chosen data blocks in $PubChall$ as the following equation:
$$AggEn\_IDTag = \prod_{i=0}^{C-1} En\_IDTag_i$$

   else
Stop

   end

Algorithm 8 DBTagProofVer

Input : $PubDBTagProof_j$, $DBProofTag$, $AggEn\_IDTag$, $PCSNonceTag_j$, ppk_{En}, $AggProofNonceTag$

Output: $DBTagProofVerResult_j$

1. Compute:

$$AggDBTagProof = \sum_{i=0}^{C-1} (PubDBTagProof_{ji}) + AggProofNonceTag \tag{6}$$

2. Encrypt $AggDBTagProof$, i.e. $En\_AggDBTagProof$, using $AggDBTagProof$ and ppk_{En} in the Paillier-En algorithm.

3. Compute:

$$DBProofTag' = DBProofTag_j + (C \times PCSNonceTag_j) \tag{7}$$

4. Encrypt $DBProofTag'$, i.e. $En\_DBProofTag$ using the Paillier-En algorithm as follows:

$$En\_DBProofTag = En(DBProofTag') \tag{8}$$

5. Compute:

$$En\_AggDBTagProof' = AggEn\_IDTag \times En\_DBProofTag \tag{9}$$

6. **if**

$$En\_AggDBTagProof == En\_AggDBTagProof' \tag{10}$$

then

Set $DBTagProofVerResult_j$=1.

else

Set $DBTagProofVerResult_j$=0.

end

Algorithm 9 DBTagProofMap

Input : $PubDBTagProof_j$, $\{ProofNonce_i\}$, $0 \leq$ i $< C$, $MappingSecretkey$, $PCSNonceTag_j$

Output: $DBTagProofMapValue_j$

1. Set $DBTagProofMapValue$=0
2. **for** *0 to C-1* **do**

   Compute: $d_i = PubDBTagProof_{ji} - PCSNonceTag_j$

   Compute:

   $$DBTagMapValue_{ji} = MappingFunction(d_i, MappingSecretkey) \quad (11)$$

   end
3. Compute:

   $$DBTagProofMapValue_j = \sum_{i=0}^{C-1} DBTagMapValue_{ji}^{ProofNonce_i} \quad (12)$$

Algorithm 10 DBTagTagProofVer

Input : $\{DBTagProofVerResult_j\}$, $\{DBTagProofMapValue_j\}$, $\{PubDBTagTagProof_j\}$, $0 \leq i < n-1$, $\{PCSNonce_j\}$, $\{En\_IDTag_i\}$, $\{ProofNonce_i\}$, $0 \leq i < C$, ppk

Output: $DBTagTagProofVerResult$, $AggPCSNonceTag$

1. **if** *(All* $\{DBTagProofVerResult_j\}$ *are equal 1) and (All* $\{DBTagProofMapValue_j\}$ *are equal)* **then**

    1. Aggregate $\{PCSNonce_j\}$ as:

    $$AggPCSNonce = \sum_{j=0}^{n-2} PCSNonce_j \tag{13}$$

    2. Compute a tag of $AggPCSNonce$, i.e. $AggPCSNonceTag$, using AS scheme.
    3. Aggregate $\{DBTagTagProof_j\}$, as follows:

    $$AggDBTagTagProof = (\prod_{j=0}^{n-2} PubDBTagTagProof_j)^{1/AggPCSNonce} \tag{14}$$

    4. **if**

    $$e(AggDBTagTagProof, g_2) == e(\prod_{i=0}^{C-1} H(En\_IDTag_i)^{ProofNonce_i} \times v^{DBTagProofMapValue}, ppk) \tag{15}$$

     then

      Set $DBTagTagProofVerResult$=1

     else

      Set $DBTagTagProofVerResult$=0

     end

 else

  Set $DBTagTagProofVerResult$=0.

 end

Algorithm 11 PriChalGen

Input : $\{I_i\}$, $\{ProofNonce_i\}$, $0 \leq i < C$

Output: $PriChall$, $LPCSNonce$

1. Choose randomly value, i.e. $LPCSNonce \in Z_p$, as a nonce for the leader provider.

2. Compute a tag of $LPCSNonce$, using AS scheme, i.e. $LPCSNonceTag$:

$$LPCSNonceTag = AS(LPCSNonce) \tag{16}$$

3. Set $PriChall$= { $\{I_i\}||\{ProofNonce_i\}$, $0 \leq i < C$ $||LPCSNonceTag$}

Algorithm 12 PriProofsGen

Input : $\{I_i\}$, $\{ProofNonce_i\}$, $\{En\text{-}DB_i\}$, $\{DBTag_i\}$, $0 \leq i < C$, $PCSNonceTag_j$
Output: $PriDBProof_j$, $PriDBTagProof_j$

1. Compute
$$PriDBProof_j = \sum_{i=0}^{C-1} (En\text{-}DB_i + ProofNonce_i) \tag{17}$$

2. Compute
$$PriDBTagProof_j = \sum_{i=0}^{C-1} (DBTag_i + PCSNonceTag_j) \tag{18}$$

Algorithm 13 FPriProofsGen

Input : $\{PriDBProof_j\}$, $\{PriDBTagProof_j\}$, $0 \leq j < n-1$, $PriDBProof_L$, $PriDBTagProof_L$
Output: $FPriDBProof, FPriDBTagProof$

1. Compute a final private $DBProof$ as follows:
$$FPriDBProof = \sum_{j=0}^{n-2} PriDBProof_j + PriDBProof_L \tag{19}$$

2. Compute a final private $DBTagProof$ as follows:
$$FPriDBTagProof = \sum_{j=0}^{n-2} PriDBTagProof_j + PriDBTagProof_L \tag{20}$$

Algorithm 14 FPriProofsVer

Input : $FPriDBProof$, $FPriDBTagProof$, $\{ProofNonce_i\}$, $\{IDTag_i\}$, $LPCSNonceTag$, $AggPCSNonceTag$, $DBTagTagProofVerResult$

Output : $FVerReslt$

1. Compute an aggregated value of $\{IDTag_i\}$, i.e. $AggIDTag$ as follows:

$$AggIDTag = \sum_{i=0}^{C-1} IDTag_i \tag{21}$$

2. Compute:

$$FPriDBTagProof_1 = n \times AggIDTag + AS(FPriDBProof) + C \times (AggPCSNonceTag + LPCSNonceTag) \tag{22}$$

3. Compute an aggregated value of $\{ProofNonce_i\}$ as follows:

$$AggProofNonce = \sum_{i=0}^{C-1} ProofNonce_i \tag{23}$$

4. Compute a tag for $AggProofNonce$, i.e. $AggProofNonceTag$, as follows:

$$AggProofNonceTag = AS(AggProofNonce) \tag{24}$$

5. Compute:

$$FPriDBTagProof_2 = FPriDBTagProof + n \times AggProofNonceTag \tag{25}$$

6. **if**

$$FPriDBTagProof_1 == FPriDBTagProof_2 \tag{26}$$

then

Set FPriProofsVer=1.

else

Set FPriProofsVer=0.

end

8. **if** *FPriProofsVer==DBTagTagProofVerResult==1* **then**

Set FVerResult=1.

else

Set FVerResult=0.

end

Algorithm 15 DataUpdateReqGen

Input : $FileID$, sk, DB, $Index$, $OpType$
Output: $DataUpdateReq$
switch ($OpType$)

case 0: //Data Block Modification

(a) Set $En\_DB_i = DB$ is an old version of the data block, and $Index$ is its ID value in the data file.

(b) Decrypt En_DB_i using the LiSHE-Dec algorithm and sk, i.e. DB_i.

(c) Set DB_i' is an new version of the data block.

(d) Encrypt DB_i' using the LiSHE-Enc algorithm and sk, i.e. En_DB_i'.

(e) Set $DataUpdateReq$= $\{OpType \mid\mid FileID \mid\mid En\_DB_i' \mid\mid Index\}$.

case 1: // Data Block Insertion

(a) Set $DB_{i+1} = DB$ is a new inserted data block which it is inserted after a data block that its position is $Index$, i.e. i.

(b) Encrypt DB_{i+1} using the LiSHE-Enc algorithm and sk, i.e. En_DB_{i+1}.

(c) Set $DataUpdateReq$= $\{OpType \mid\mid FileID \mid\mid En\_DB_{i+1} \mid\mid Index\}$.

case 2: // Data Block Deletion

(a) Set $DataUpdateReq$= $\{OpType \mid\mid FileID \mid\mid Index\}$

end switch

Algorithm 16 DataUpdate

Input : $DataUpdateReq$
Output : $UpdateResult$
switch ($OpType$)

case 0**:** // Data Block Modification

if En_DB_i *is associated to other data files* **then**

if $En\_DB'_i$ *is duplicated* **then**

1. Execute the operations of Mod-Case_2 for updating LPCS-M2T.
2. Set $UpdateResult$= 1—— ID // ID is an identifier of the data block that is identical to $En\_DB'_i$ in NonDuplicatedDB/Tag table.

else

1. Execute the operations of Mod-Case_1.
2. Set $UpdateResult$= 1——0.

end

else

if $En\_DB'_i$ *is duplicated* **then**

1. Execute the operations of Mod-Case_4.
2. Set $UpdateResult$= 0—— ID // ID is an identifier of the data block that is identical to $En\_DB'_i$.

else

1. Execute the operations of Mod-Case_3.
2. Set $UpdateResult$= 0——0.

end

end

case 1**:** // Data Block Insertion

(a) **if** En_DB_{i+1} *is duplicated* **then**

1. Execute the operations of Ins-Case_1.
2. Set $UpdateResult$= ID // ID is an identifier of the data block that is identical to En_DB_{i+1}.

else

1. Execute the operations of Ins-Case_2.
2. Set $UpdateResult$= 0.

end

case 2**:** //Data Block Deletion

(a) **if** *a data block,* En_DB_i*, is associated to other files,* **then**

1. Execute the operations of Del-Case_1
2. Set $UpdateResult$= 1,

else

1. Execute the operations of Del-Case_2.
2. set $UpdateResult$= 0.

end

end switch

Algorithm 17 TagsOfUpdatedDataGen

Input : $DataUpdateReq$, $UpdateResult$, $User_{ID}$, x, υ, ppk_{En}, K
Output: $UpdatedTags$, $En\_IDTagUpdateReq$
switch ($OpType$)

case 0**:** // Data Block Modification

(a) **if** *The first item in* $UpdateResult$= *1* **then**

if *The second item in* $UpdateResult$==*0* **then**

1. Compute new tags for En_DB_i', i.e. $IDTag_i$, $DBTag_i$, $DBTagTag_i$ and En_IDTag_i, using TagGen algorithm and $User_{ID}$, x, υ, ppk_{En}.
2. Execute the operations of Mod-Case_1 for updating User-M2T.
3. Set $UpdatedTags = DBTag_i \mid\mid DBTagTag_i$
4. Set $En\_IDTagUpdateReqt$={$OpType$ || $FileID$ || $UpdateResult$ || En_IDTag_i || $Index$}.

else

1. Execute the operations of Mod-Case_2 for updating User-M2T and using ID value.
2. Set $UpdatedTags = 1$.
3. Set $En\_IDTagUpdateReq$={$OpType$ || $FileID$ || $UpdateResult$ || $Index$ }.

end

else

if *The second item in* $UpdateResult$==*0* **then**

1. Compute new tags for En_DB_i', i.e. $IDTag_i$, $DBTag_i$, $DBTagTag_i$ and En_IDTag_i.
2. Execute the operations of Mod-Case_3.
3. Set $UpdatedTags = DBTag_i \mid\mid DBTagTag_i$
4. Set $En\_IDTagUpdateReqt$={$OpType$ || $FileID$ || $UpdateResult$ || En_IDTag_i || $Index$}.

else

1. Execute the operations of Mod-Case_4.
2. Set $UpdatedTags = 1$.
3. Set $En\_IDTagUpdateReq$={$OpType$ || $FileID$ || $UpdateResult$ || $Index$ }.

end

end

case 1**:** // Data Block Insertion

if $UpdateResult$==*0* **then**

1. Compute new tags for En_DB_i', i.e. $IDTag_i$, $DBTag_i$, $DBTagTag_i$ and En_IDTag_i.
2. Execute the operations of Ins-Case_2.
3. Set $UpdatedTags = DBTag_i \mid\mid DBTagTag_i$
4. Set $En\_IDTagUpdateReqt$={$OpType$ || $FileID$ || $UpdateResult$ || En_IDTag_i || $Index$}.

else

1. Execute the operations of Ins-Case_1.
2. Set $UpdatedTags = 1$.
3. Set $En\_IDTagUpdateReq$={$OpType$ || $FileID$ || $UpdateResult$ || $Index$ }.

end

case 2**:** // Data Block Deletion

(a) **if** *UpdateResult ==1* **then**

1. Execute the operations of Del-Case_1.
2. Set $UpdatedTags$ = 1.
3. Set $En\_IDTagUpdateReq$={$OpType$ || $FileID$ || $UpdateResult$ || $Index$ }.

else

1. Execute the operations of Del-Case_2.
2. Set $UpdatedTags$ = 1.
3. Set $En\_IDTagUpdateReq$={$OpType$ || $FileID$ || $UpdateResult$ || $Index$ }.

end

end switch

2. Increase the value of K by one in the case of an insert operation or decrease it by one in the case of a delete operation to update $FileTagSig$ of the data file using Algorithm 4.

Algorithm 18 PCSUpdateReqGen

Input : $DataUpdateReq$, $UpdateResult$, $UpdatedTags$
Output: $PCSUpdateReq$

1. **switch** ($OpType$)

   case 0**:** // Data Block Modification

   if *The second item in* $UpdateResult$==0 **then**

   Set $PCSUpdateReq$=$\{OpType \mid\mid FileID \mid\mid UpdateResult \mid\mid En\_DB'_i \mid\mid Index \mid\mid UpdatedTags\}$

   else

   Set $PCSUpdateReq$=$\{OpType \mid\mid FileID \mid\mid UpdateResult \mid\mid \mid\mid Index \mid\mid UpdatedTags\}$

   end

   case 1**:** // Data Block Insertion

   if $UpdateResult$==0 **then**

   Set $PCSUpdateReq$=$\{OpType \mid\mid FileID \mid\mid UpdateResult \mid\mid En\_DB_{i+1} \mid\mid Index \mid\mid UpdatedTags\}$

   else

   Set $PCSUpdateReq$=$\{OpType \mid\mid FileID \mid\mid UpdateResult \mid\mid \mid\mid Index \mid\mid UpdatedTags\ \}$

   end

   case 2**:** // Data Block Deletion

   if $UpdateResult$ ==0 **then**

   Set $PCSUpdateReq$=$\{OpType \mid\mid FileID \mid\mid UpdateResult \mid\mid \mid\mid Index \mid\mid UpdatedTags\ \}$

   else

   Set $PCSUpdateReq$=$\{OpType \mid\mid FileID \mid\mid UpdateResult \mid\mid \mid\mid Index \mid\mid UpdatedTags\}$

   end

   end switch

Algorithm 19 DataTagsUpdate

Input : $PCSUpdateReq$
Output: ACK

1. **switch** ($OpType$)

   case 0 **:** // Data Block Modification

   if *The first item in* $UpdateResult$*==1* **then**

   if *The second item in* $UpdateResult$*==0* **then**
   Execute the operations of Mod-Case_1.
   else
   Execute the operations of Mod-Case_2.
   end

   else

   if *The second item in* $UpdateResult$*==0* **then**
   Execute the operations of Mod-Case_3.
   else
   Execute the operations of Mod-Case_4.
   end

   end

   case 1**:** // Data Block Insertion

   if $UpdateResult$*=0* **then**

   Execute the operations of Ins-Case_2.

   else

   Execute the operations of Ins-Case_1.

   end

   case 2**:** // Data Block Deletion

   if $UpdateResult$*=0* **then**

   Execute the operations of Del-Case_2.

   else

   Execute the operations of Del-Case_1.

   end

   end switch

2. Set ACK= 1

Algorithm 20 En_IDTagUpdate

Input : $En\_IDTagUpdateReq$
Output: ACK

1. **switch** ($OpType$)

   case 0 **:** // Data Block Modification

   if *The first item in* $UpdateResult$*==1* **then**

   if *The second item in* $UpdateResult$*==0* **then**
   Execute the operations of Mod-Case_1.
   else
   Execute the operations of Mod-Case_2.
   end

   else

   if *The second item in* $UpdateResult$*==0* **then**
   Execute the operations of Mod-Case_3.
   else
   Execute the operations of Mod-Case_4.
   end

   end

   case 1**:** // Data Block Insertion

   if $UpdateResult$*=0* **then**
   Execute the operations of Ins-Case_2.
   else
   Execute the operations of Ins-Case_1.
   end

   case 2**:** // Data Block Deletion

   if $UpdateResult$*=0* **then**
   Execute the operations of Del-Case_2.
   else
   Execute the operations of Del-Case_1.
   end

   end switch

2. Set ACK= 1
