# Supplementary material for: A novel approach to data integrity auditing in PCS: Minimising any Trust on Third Parties (DIA-MTTP)
Source: PLoS One. 2021 Jan 7;16(1):e0244731. doi: 10.1371/journal.pone.0244731 (PMC7790547; doi:10.1371/journal.pone.0244731)
Supplement: S4 File — (PDF) [file pone.0244731.s004.pdf]

Correctness of LoA1DV and LoA2DV Protocols

DBTagProof Public Verification

The Public verification of $DBTagProof$ is performed using EQ(22), i.e.,

$$En\_AggDBTagProof' = En\_AggDBTagProof$$

The proof of the above equation as follows:

Left side: $En\_AggDBTagProof'$

$$
\begin{aligned}
&= AggEn\_IDTag \times En\_DBProofTag, \text{(based EQ(21))}\\
&= AggEn\_IDTag \times E(DBProofTag'), \text{(based EQ(20))}\\
&= \prod_{i=0}^{C-1} En\_IDTag \times E(DBProofTag_j + (C \times PCSNonceTag_j)), \text{(EQ(19))}\\
&= E(\sum_{i=0}^{C-1} IDTag + DBProofTag_j + C \times PCSNonceTag_j), \text{(based EQ(37))}\\
&= E(\sum_{i=0}^{C-1} IDTag + AS(DBProof) + C \times PCSNonceTag_j)\\
&= E(\sum_{i=0}^{C-1} IDTag + AS(\sum_{i=0}^{C-1} (En\_DB_i + ProofNonce_i)) + C \times PCSNonceTag_j), \text{(based EQ(15))}\\
&= E(\sum_{i=0}^{C-1} IDTag + \sum_{i=0}^{C-1} (AS(En\_DB_i) + AS(ProofNonce_i)) + C \times PCSNonceTag_j)\\
&= E(\sum_{i=0}^{C-1} IDTag + \sum_{i=0}^{C-1} DataTag_i + \sum_{i=0}^{C-1} AS(ProofNonce_i) + C \times PCSNonceTag_j)\\
&= E(\sum_{i=0}^{C-1} (IDTag + DataTag_i) + \sum_{i=0}^{C-1} AS(ProofNonce_i) + C \times PCSNonceTag_j)\\
&= E(\sum_{i=0}^{C-1} DBTag_i + \sum_{i=0}^{C-1} AS(ProofNonce_i) + C \times PCSNonceTag_j), \text{(based EQ(2))}\\
&= E(\sum_{i=0}^{C-1} DBTag_i + C \times PCSNonceTag_j + \sum_{i=0}^{C-1} AS(ProofNonce_i)), \text{(based EQ(16))}\\
&= E(\sum_{i=0}^{C-1} DBTagProof_{ji} + \sum_{i=0}^{C-1} AS(ProofNonce_i))\\
&= E(\sum_{i=0}^{C-1} (DBTagProof_{ji}) + AggProofNonceTag)\\
&= E(AggDBTagProof), \text{(based EQ(17))}\\
&= En\_AggDBTagProof
\end{aligned}
$$

DBTagTagProof Verification

The verifaction of $DBTagTagProof$ is performed using EQ(26), i.e.,

$$e(AggDBTagTagProof, g_2) = e(\prod_{i=0}^{C-1} H(En\_IDTag_i)^{ProofNonce_i} \times \upsilon^{DBTagProofMapValue}, ppk)$$

The proof of the above equation as follows:

$$
\begin{aligned}
AggDBTagTagProof &= (\prod_{j=0}^{n-2}(\prod_{i=0}^{C-1} DBTagTag_i^{ProofNonce_i})^{PCSNonce_j})^{1/AggPCSNonce} \\
&= ((\prod_{i=0}^{C-1} DBTagTag_i^{ProofNonce_i})^{\sum_{j=0}^{n-2} PCSNonce_j})^{1/AggPCSNonce} \\
&= ((\prod_{i=0}^{C-1} DBTagTag_i^{ProofNonce_i})^{AggPCSNonce})^{1/AggPCSNonce}, \text{(based on EQ(41))}
\end{aligned}
$$

$$
= \prod_{i=0}^{C-1} DBTagTag_i^{ProofNonce_i} \tag{39}
$$

Left Side: $e(AggDBTagTagProof, g_2) = e(\prod_{i=0}^{C-1} DBTagTag_i^{ProofNonce_i}, g_2)$

Right Side: $e(\prod_{i=0}^{C-1} H(En\_IDTag_i)^{ProofNonce_i} \times \upsilon^{DBTagProofMapValue}, ppk)$

$= e(\prod_{i=0}^{C-1} H(En\_IDTag_i)^{ProofNonce_i} \times \upsilon^{\sum_{i=0}^{C-1} DBTagMapValue_i^{ProofNonce_i}}, ppk)$, (based on EQ(23))

$= e(\prod_{i=0}^{C-1} H(En\_IDTag_i)^{ProofNonce_i} \times \prod_{i=0}^{C-1} \upsilon^{DBTagMapValue_i^{ProofNonce_i}}, g_2^x)$, (Based on $ppk = g_2^x$)

$= e(\prod_{i=0}^{C-1} [H(En\_IDTag_i) \times \upsilon^{DBTagMapValue_i}]^{ProofNonce_i}, g_2^x)$

$= e(\prod_{i=0}^{C-1} [H(En\_IDTag_i) \times \upsilon^{DBTagMapValue_i}]^{ProofNonce_i \times x}, g_2)$, based on property (P1) of the bilinear pairing

$= e(\prod_{i=0}^{C-1} [H(En\_IDTag_i) \times \upsilon^{DBTagMapValue_i}]^{x\, ProofNonce_i}, g_2)$

$= e(\prod_{i=0}^{C-1} DBTagTag_i^{ProofNonce_i}, g_2)$, (based on EQ(23))

$= e(AggDBTagTagProof, g_2)$, (based on EQ(39)), Left-side.

DBTagProof Private Verification

The private verification is based on EQ(35), i.e. $FPriDBTagProof_1 == FPriDBTagProof_2$. The proof of the above equation as follows:

Left side: $FPriDBTagProof_1 = n \times AggIDTag + AS(FPriDBProof) + C \times (AggPCSNonceTag + LPCSNonceTag)$

Right side: $FPriDBTagProof_2 = FPriDBTagProof + n \times AggProofNonceTag$

$$\text{Left Side} = n \times (\sum_{i=0}^{C-1} IDTag_i) + AS(\sum_{j=0}^{n-2} PriDBProof_j + PriDBProof_L) + C \times (AggPCSNonceTag + LPCSNonceTag)$$

$$, \text{(EQ(30))}$$

$$= n \times (\sum_{i=0}^{C-1} IDTag_i) + AS(\sum_{j=0}^{n-2} PriDBProof_j) + AS(PriDBProof_L) + C \times (AggPCSNonceTag + LPCSNonceTag)$$

$$, \text{(based EQ(27))}$$

$$= n \times (\sum_{i=0}^{C-1} IDTag_i) + AS(\sum_{j=0}^{n-2} \sum_{i=0}^{C-1} (En\text{-}DB_{ji} + ProofNonce_i)) + AS(\sum_{i=0}^{C-1} (En\text{-}DB_{Li} + ProofNonce_i))$$

$$+ C \times (AggPCSNonceTag + LPCSNonceTag), \text{(based EQ(15))}$$

$$= n \times (\sum_{i=0}^{C-1} IDTag_i) + \sum_{j=0}^{n-2} \sum_{i=0}^{C-1} (AS(En\text{-}DB_{ji}) + AS(ProofNonce_i)) + \sum_{i=0}^{C-1} (AS(En\text{-}DB_{Li}) + AS(ProofNonce_i))$$

$$+ C \times (AggPCSNonceTag + LPCSNonceTag)$$

$$= n \times (\sum_{i=0}^{C-1} IDTag_i) + \sum_{j=0}^{n-2} \sum_{i=0}^{C-1} (DataTag_{ji} + ProofNonceTag_i) + \sum_{i=0}^{C-1} (DataTag_{Li} + ProofNonceTag_i)$$

$$+ C \times (AggPCSNonceTag + LPCSNonceTag), \text{(based EQ(3))}$$

$$= \sum_{j=0}^{n-2} (\sum_{i=0}^{C-1} (IDTag_i + DataTag_{ji}) + n - 2 \times \sum_{i=0}^{C-1} ProofNonceTag_i$$

$$+ \sum_{i=0}^{C-1} (IDTag_i + DataTag_{Li}) + \sum_{i=0}^{C-1} ProofNonceTag_i) + C \times (AggPCSNonceTag + LPCSNonceTag)$$

$$= \sum_{j=0}^{n-2} (\sum_{i=0}^{C-1} (DBTag_{ji}) + n - 2 \times \sum_{i=0}^{C-1} ProofNonceTag_i + \sum_{i=0}^{C-1} (DBTag_{Li}) + \sum_{i=0}^{C-1} ProofNonceTag_i)$$

$$+ C \times (AggPCSNonceTag + LPCSNonceTag), \text{(based EQ(2))}$$

$$= \sum_{j=0}^{n-2} (\sum_{i=0}^{C-1} (DBTag_{ji}) + C \times (\sum_{j=0}^{n-2} PCSNonceTag_j) + n - 2 \times \sum_{i=0}^{C-1} ProofNonceTag_i + \sum_{i=0}^{C-1} (DBTag_{Li})$$

$$+ \sum_{i=0}^{C-1} ProofNonceTag_i) + C \times LPCSNonceTag, \text{(based EQ(15))}$$

$$= \sum_{j=0}^{n-2} (\sum_{i=0}^{C-1} (DBTag_{ji} + PCSNonceTag_j) + \sum_{i=0}^{C-1} (DBTag_{Li} + LPCSNonceTag) + n \times \sum_{i=0}^{C-1} ProofNonceTag_i$$

$$, \text{(based EQ(15))}$$

$$= \sum_{j=0}^{n-2} PriDBTagProof_j + PriDBTagProof_L + n \times AggProofNonceTag, \text{(based EQ(29))}$$

$$= FPriDBTagProof + n \times AggProofNonceTag, \text{(based EQ(31)), Right side, it holds true.}$$
