# Supplementary material for: A novel approach to data integrity auditing in PCS: Minimising any Trust on Third Parties (DIA-MTTP)
Source: PLoS One. 2021 Jan 7;16(1):e0244731. doi: 10.1371/journal.pone.0244731 (PMC7790547; doi:10.1371/journal.pone.0244731)
Supplement: S5 File — (PDF) [file pone.0244731.s005.pdf]

Complexities of Computational and Communication cost in DIA-MTTP

Table 1: Complexities of computational cost incurred at the user in D3U.

	DFProCost	DFTagGenCost
Without Data Deduplication based	$K \times (2 \times Multi_{Z_p} + Add_{Z_p} + Exp_{Z_p})$	$K \times (2 \times AS{-}G + Add_{AS} + 2 \times Exp_{Z_{n^2}} + Multi_{Z_{n^2}} + H1 + H_{G_1} + Multi_{G_1} + 2 \times Exp_{G_1}) + H1 + Exp_{Z_n}$
D3L1 based	$d1 \times (2 \times Multi_{Z_p} + Add_{Z_p} + Exp_{Z_p})$	$d1 \times (2 \times AS{-}G + Add_{AS} + 2 \times Exp_{Z_{n^2}} + Multi_{Z_{n^2}} + H1 + H_{G_1} + Multi_{G_1} + 2 \times Exp_{G_1}) + H1 + Exp_{Z_n}$
D3L2 based	$K \times (2 \times Multi_{Z_p} + Add_{Z_p} + Exp_{Z_p})$	$d2 \times (2 \times AS{-}G + Add_{AS} + 2 \times Exp_{Z_{n^2}} + Multi_{Z_{n^2}} + H1 + H_{G_1} + Multi_{G_1} + 2 \times Exp_{G_1}) + H1 + Exp_{Z_n}$
D3L1/D3L2 based	$d1 \times (2 \times Multi_{Z_p} + Add_{Z_p} + Exp_{Z_p})$	$d2 \times (2 \times AS{-}G + Add_{AS} + 2 \times Exp_{Z_{n^2}} + Multi_{Z_{n^2}} + H1 + H_{G_1} + Multi_{G_1} + 2 \times Exp_{G_1}) + H1 + Exp_{Z_n}$

Table 2: Complexities on communication cost incurred at the user in D3U.

Non-deduplication and Hierarchical based	$K \times (\lvert En\_DB \rvert + \lvert DBTag \rvert + \lvert DBTagTag \rvert + \lvert En\_IDTag \rvert) = K \times (\lvert p \rvert + \lvert m \rvert + \lvert G_1 \rvert + \lvert n^2 \rvert)$
Deduplication and Hierarchical based	$d1 \times (\lvert En\_DB \rvert) + d2 \times (\lvert DBTag \rvert + \lvert DBTagTag \rvert + \lvert En\_IDTag \rvert) = d1 \times (\lvert p \rvert) + d2 \times (\lvert m \rvert + \lvert G_1 \rvert + \lvert n^2 \rvert)$
Non-Deduplication and Non-Hierarchical based	$n \times K \times (\lvert En\_DB \rvert + \lvert DBTag \rvert + \lvert DBTagTag \rvert + \lvert En\_IDTag \rvert) = n \times K \times (\lvert p \rvert + \lvert m \rvert + \lvert G_1 \rvert + \lvert n^2 \rvert)$

Table 3: Complexities of the communication cost of the leader provider and the non leader provider in D3U.

	The leader provider	The non leader provider
Non-Deduplication and Hierarchical based	$K \times (\lvert En\_DB \rvert + \lvert DBTag \rvert + \lvert DBTagTag \rvert) +$ —ACK—$= K \times (\lvert p \rvert + \lvert m \rvert + \lvert G_1 \rvert) +$ —ACK—	$\lvert ACK \rvert$
Deduplication and Hierarchical based	$n \times (d1 - d2) \times L_{DBID} + (n-1) \times d2 \times (\lvert p \rvert + \lvert m \rvert + \lvert G_1 \rvert) + \lvert ACK \rvert$	$\lvert ACK \rvert$
Non-Deduplication and Non-Hierarchical based	$\lvert ACK \rvert$	$\lvert ACK \rvert$

($(d1 - d2)$ *is the total number of duplicated data blocks and* L_{DBID} *is the bit-length of the ID of the data block*)

Table 4: Complexities of the computational cost incurred by the non leader provider in LoA1DV: With/without nonces.

	With nonces	Without nonces
The non leader provider	$(2 \times C - 1) \times Add_{Z_p} + C \times Add_{AS} + (C - 1) \times Multi_{G_1} + (C + 1) \times Exp_{G_1}$	$(C - 1) \times (Add_{Z_p} + Add_{AS} + Multi_{G_1})$

Table 5: Complexities in the computational cost incurred by the TPAs in LoA1DV (using nonces).

The L-TPA	$(C + n - 3) \times Add_{Z_p} + (n + 2) \times AS\text{–}G + (C - 1) \times Multi_{Z_{n^2}} + (C + n - 2) \times Multi_{G_1} + 2 \times Pair_{G_1,G_2} + 3 \times Exp_{G_1} + C \times H_{G_1}$
The non leader TPA	$2 \times C \times Add_{AS} + 4 \times Exp_{Z_{n^2}} + 3 \times Multi_{Z_{n^2}} + C \times (H1 + Exp_{Zp}) + (C - 1) \times Add_{Z_p}$

Table 6: Complexities of the computational cost incurred by the TPAs end in LoA1DV: With/without the collaborative and nonces approaches.

	With nonces	Without nonces
With collaborative verification	$(C + n - 3) \times Add_{Z_p} + (n + 2) \times AS\text{–}G + (C - 1) \times Multi_{Z_{n^2}} + (C + n - 2) \times Multi_{G_1} + 2 \times Pair_{G_1,G_2} + 3 \times Exp_{G_1} + C \times H_{G_1} + (n - 1) \times (2 \times C \times Add_{AS} + 4 \times Exp_{Z_{n^2}} + 3 \times Multi_{Z_{n^2}} + C \times H1 + C \times Exp_{Zp} + (C - 1) \times Add_{Z_p})$	$AS\text{–}G + (C - 1) \times Multi_{Z_{n^2}} + (C + n - 2) \times Multi_{G_1} + 2 \times Pair_{G_1,G_2} + Exp_{G_1} + C \times H_{G_1} + (n - 1) \times ((C - 1) \times Add_{AS} + 4 \times Exp_{Z_{n^2}} + 3 \times Multi_{Z_{n^2}} + C \times H1 + (C - 1) \times Add_{Z_p})$
Without collaborative verification	$n \times ((2 \times C + n - 4) \times Add_{Z_p} + (n + 2) \times AS\text{–}G + 2 \times C \times Add_{AS} + 4 \times Exp_{Z_{n^2}} + (C + 3) \times Multi_{Z_{n^2}} + C \times H1 + C \times Exp_{Zp} + (C + n - 2) \times Multi_{G_1} + 2 \times Pair_{G_1,G_2} + 3 \times Exp_{G_1} + C \times H_{G_1})$	$n \times (AS\text{–}G + (C - 1) \times Add_{AS} + 4 \times Exp_{Z_{n^2}} + (C + 3) \times Multi_{Z_{n^2}} + C \times H1 + (C - 1) \times Add_{Z_p} + (C + n - 2) \times Multi_{G_1} + 2 \times Pair_{G_1,G_2} + Exp_{G_1} + C \times H_{G_1})$

Table 7: Complexities of computational cost incurred at the user in LoA2DV : With/without nonces.

	With nonces	Without nonces
With hierarchical approach	$(3 \times C + 2 \times n - 1) \times Add_{AS} + AS{-}G$	$(C + n - 1) \times Add_{AS} + AS{-}G$
Without hierarchical approach	$(3 \times (C + n) - 2 \times Add_{AS} + AS{-}G + (n-1) \times Add_{Z_p}$	$(C + 2 \times n - 1) Add_{AS} + AS{-}G + (n-1) \times Add_{Z_p}$

Table 8: Complexities of the computational cost incurred by providers in the LoA2DV: With/without nonces.

	With nonces	Without nonces
The leader provider	$2 \times C + n - 2 \times (Add_{Z_p} + Add_{AS})$	$2 \times (C + n - 2) \times (Add_{Z_p} + Add_{AS})$
The non leader provider	$(4 \times C - 2) \times Add_{Z_p} + (3 \times C - 1) \times Add_{AS} + (C-1) \times Multi_{G_1} + (C+1) \times Exp_{G_1}$	$(2 \times C - 2) \times (Add_{Z_p} + Add_{AS} + Multi_{G_1})$

Table 9: Complexities of the communication cost for providers in LoA1DV, and LoA2DV.

	LoA1DV	LoA2DV
The leader provider	-	$(n\text{-}1) \times C \times (\lvert I_i \rvert + \lvert ProofNonce_i \rvert) + \lvert FPriDBProof \rvert + \lvert FPriDBTagProof \rvert = (n\text{-}1) \times C \times (\lvert I_i \rvert + \lvert p \rvert) + \lvert p \rvert + \lvert m \rvert$
The non leader provider	$\lvert PubDBProof \rvert + \lvert PubDBTagProof \rvert + \lvert PubDBTagTagProof \rvert = \lvert p \rvert + C \times (\lvert m \rvert) + \lvert G_1 \rvert$	$\lvert PriDBProof \rvert + \lvert PriDBTagProof \rvert + \lvert PubDBProof \rvert + \lvert PubDBTagProof \rvert + \lvert PubDBTagTagProof \rvert = 2 \times \lvert p \rvert + (C+1) \times \lvert m \rvert + \lvert G_1 \rvert$

Table 10: Complexities of the communication cost for the L-TPA and TPAs in LoA1DV and LoA2DV.

	With nonces	Without nonces
L-TPA (LoA1DV)	$(n\text{-}1) \times (C \times (\lvert I_i \rvert + \lvert ProofNonce_i \rvert) + \lvert AggProofNonceTag \rvert + \lvert DBProofTag \rvert + \lvert AggEn\_IDTag \rvert + \lvert PCSNonce \rvert + \lvert PCSNonceTag \rvert) = (n\text{-}1) \times (C \times (\lvert I_i \rvert + \lvert p \rvert) + 3 \times \lvert m \rvert + \lvert n^2 \rvert + \lvert P \rvert)$	$(n\text{-}1) \times (C \times \lvert I_i \rvert + \lvert DBProofTag \rvert + \lvert AggEn\_IDTag \rvert) = (n\text{-}1) \times (C \times \lvert I_i \rvert + \lvert m \rvert + \lvert n^2 \rvert)$
L-TPA (LoA2DV)	$(n\text{-}1) \times (C \times (\lvert I_i \rvert + \lvert ProofNonce_i \rvert) + \lvert AggProofNonceTag \rvert + \lvert DBProofTag \rvert + \lvert AggEn\_IDTag \rvert + \lvert PCSNonce \rvert + \lvert PCSNonceTag \rvert) + C \times (\lvert I_i \rvert + \lvert ProofNonce_i \rvert) + \lvert AggPCSNonceTag \rvert = (n\text{-}1) \times (C \times (\lvert I_i \rvert + \lvert p \rvert) + 3 \times \lvert m \rvert + \lvert n^2 \rvert + \lvert p \rvert) + C \times (\lvert I_i \rvert + \lvert p \rvert) + \lvert m \rvert$	$(n\text{-}1) \times (C \times \lvert I_i \rvert + \lvert DBProofTag \rvert + \lvert AggEn\_IDTag \rvert) + C \times \lvert I_i \rvert = (n\text{-}1) \times (C \times \lvert I_i \rvert + \lvert m \rvert + \lvert n^2) + C \times \lvert I_i \rvert$
The non leader TPA (LoA1DV/LoA2DV)	$C \times (\lvert I_i \rvert + \lvert ProofNonce_i \rvert) + \lvert PCSNonce \rvert + \lvert PCSNonceTag \rvert + \lvert PubDBProof \rvert + \lvert PubDBTagTagProof \rvert + \lvert PubDBTagProofVerResult \rvert + \lvert DBTagTagMapValue \rvert = C \times \lvert I_i \rvert + (C+3) \times \lvert p \rvert + \lvert m \rvert + \lvert G_1 \rvert + 1$	$C \times \lvert I_i \rvert + \lvert PubDBProof \rvert + \lvert PubDBTagTagProof \rvert + \lvert PubDBTagProofVerResult \rvert + \lvert DBTagTagMapValue \rvert = C \times \lvert I_i \rvert + 2 \times \lvert p \rvert + \lvert G_1 \rvert + 1$

(using NonKey-based approach)

Table 11: Complexities of the computational cost incurred by the user in D3U and DU: With/without data deduplication.

Uploading Non-duplicated data block	$(2 \times Multi_{Z_p} + Add_{Z_p} + Exp_{Z_p}) + (2 \times AS{-}G + Add_{AS} + 2 \times Exp_{Z_{n^2}} + Multi_{Z_{n^2}} + H1 + H_{G_1} + Multi_{G_1} + 2 \times Exp_{G_1})$
Uploading duplicated data block	$(2 \times Multi_{Z_p} + Add_{Z_p} + Exp_{Z_p})$
Inserting Non-duplicated data block	$(2 \times Multi_{Z_p} + Add_{Z_p} + Exp_{Z_p}) + (2 \times AS{-}G + Add_{AS} + 2 \times Exp_{Z_{n^2}} + Multi_{Z_{n^2}} + H1 + H_{G_1} + Multi_{G_1} + 2 \times Exp_{G_1})$
Inserting duplicated data block	$(2 \times Multi_{Z_p} + Add_{Z_p} + Exp_{Z_p})$
Modifying Non-duplicated data block	$3 \times Multi_{Z_p} + Add_{Z_p} + 2 \times Exp_{Z_p} + (2 \times AS{-}G + Add_{AS} + 2 \times Exp_{Z_{n^2}} + Multi_{Z_{n^2}} + H1 + H_{G_1} + Multi_{G_1} + 2 \times Exp_{G_1})$
Modifying duplicated data block	$3 \times Multi_{Z_p} + Add_{Z_p} + 2 \times Exp_{Z_p}$
